# Supplementary figures and images for: Time-resolved, integrated analysis of clonally evolving genomes
Source: PLoS Genet. 2023 Dec 14;19(12):e1011085. doi: 10.1371/journal.pgen.1011085 (PMC10754456; doi:10.1371/journal.pgen.1011085)

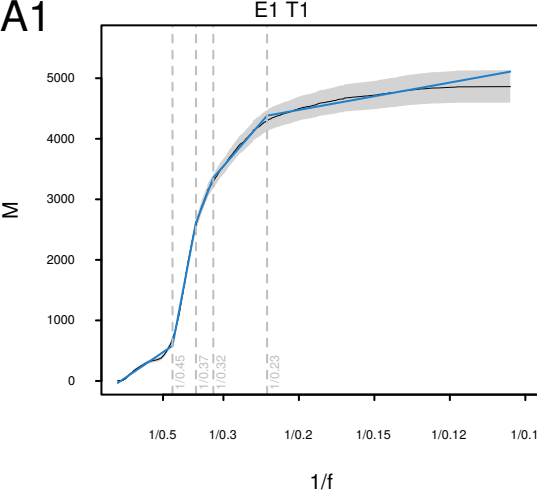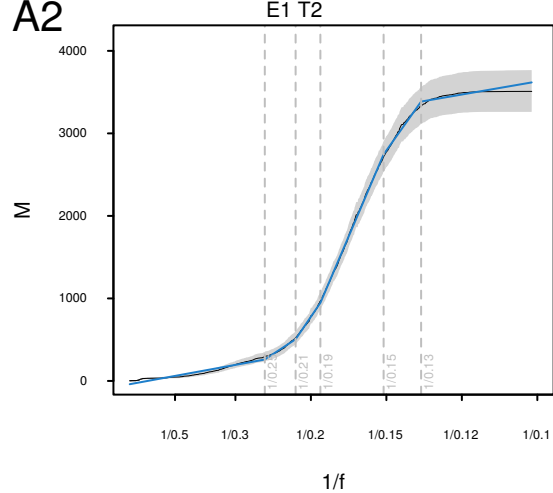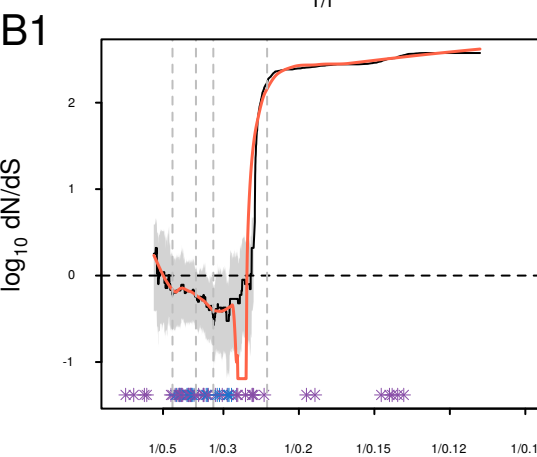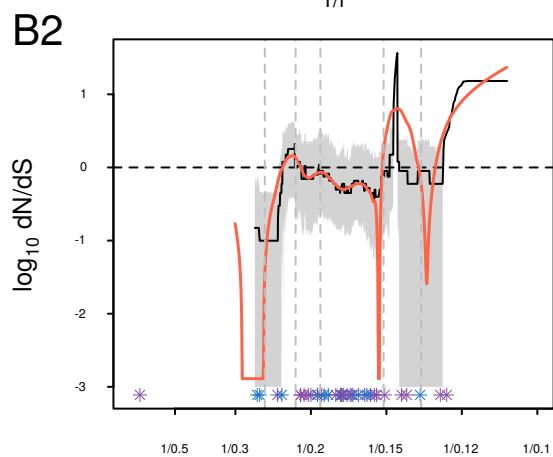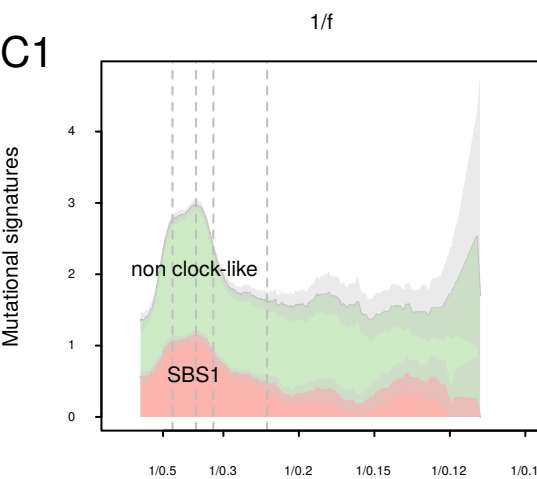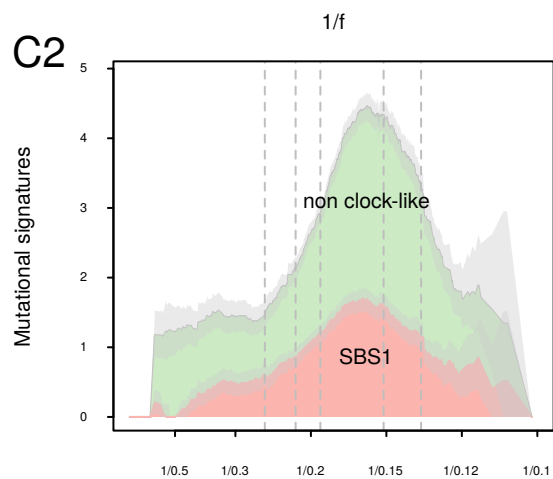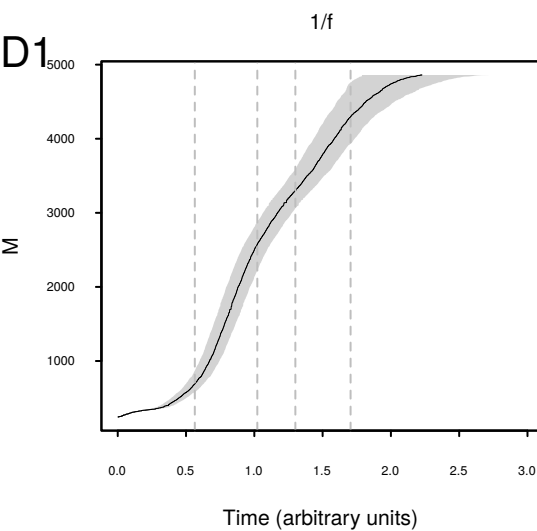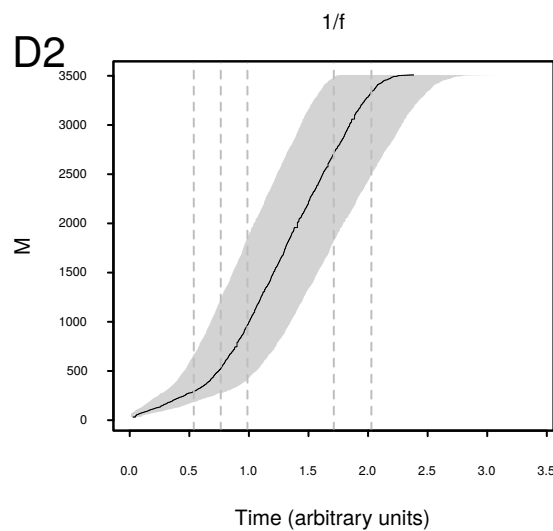

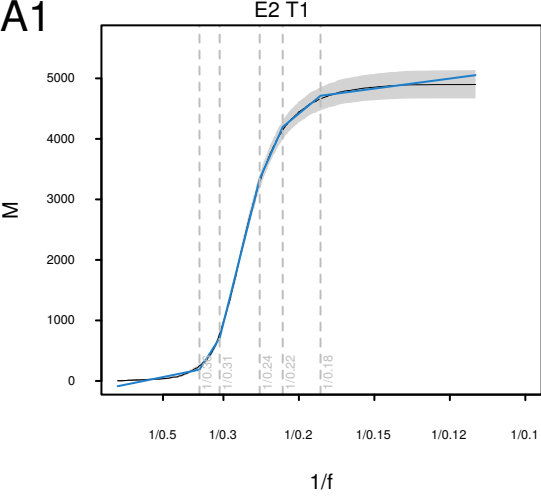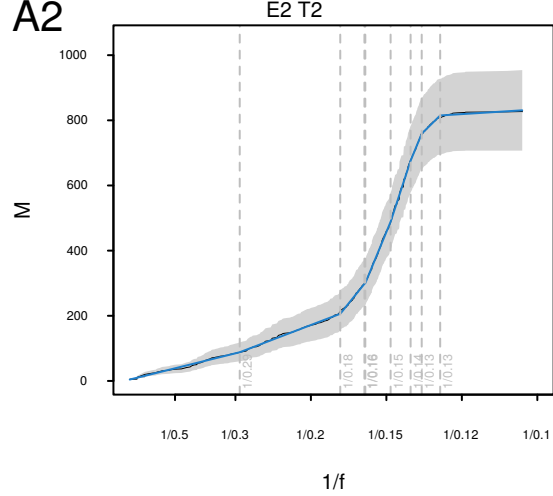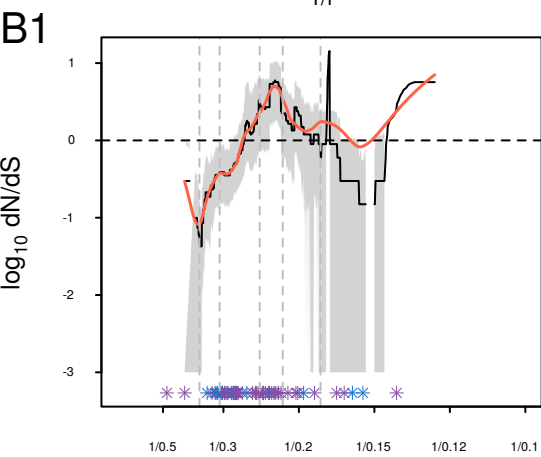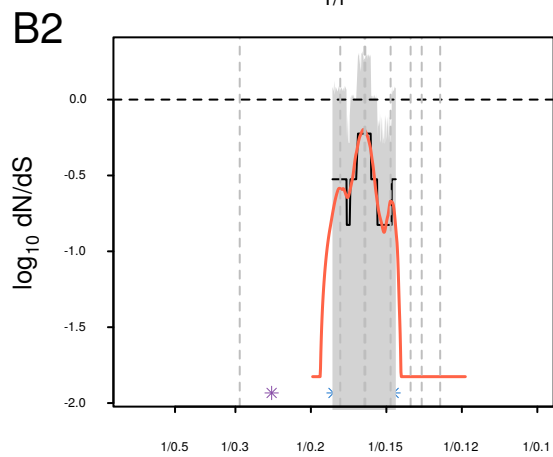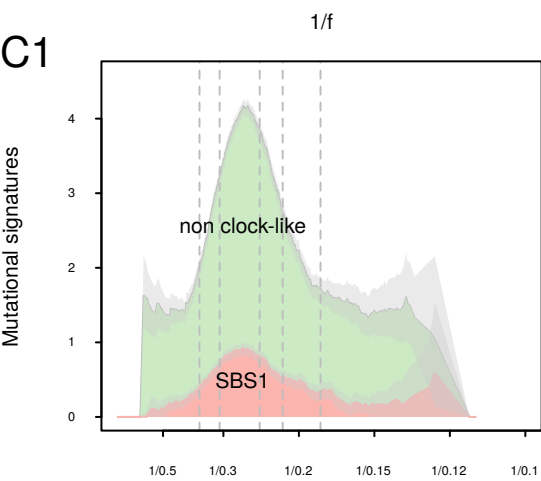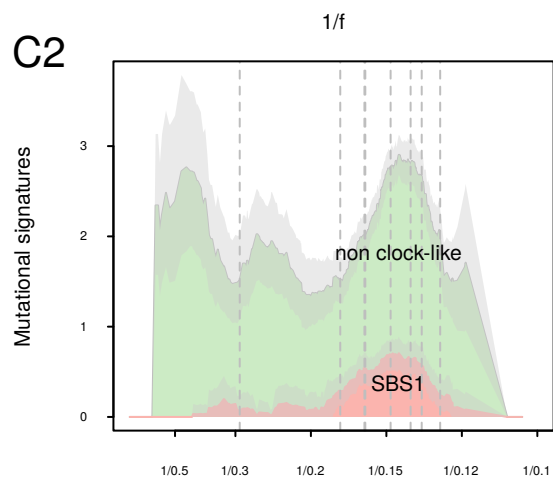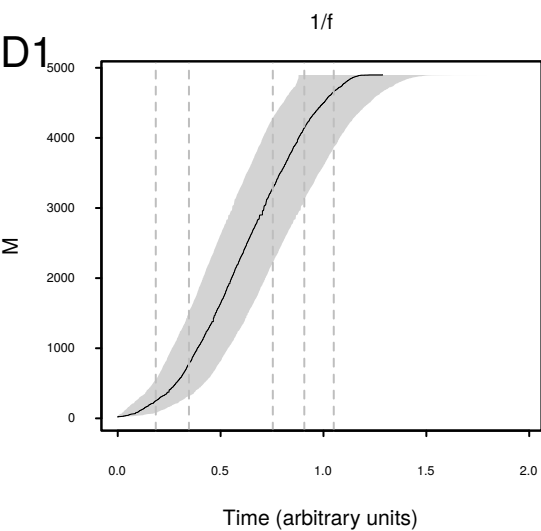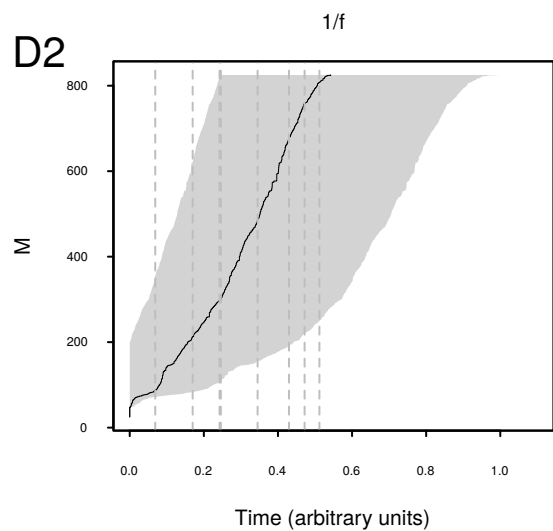

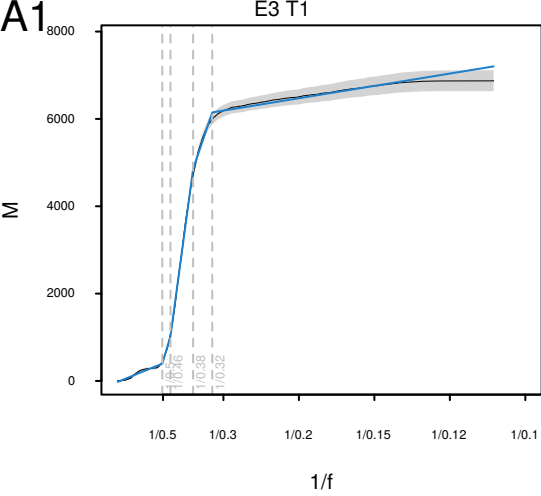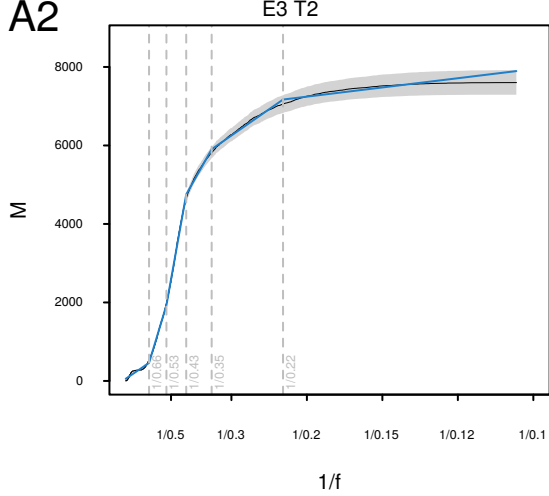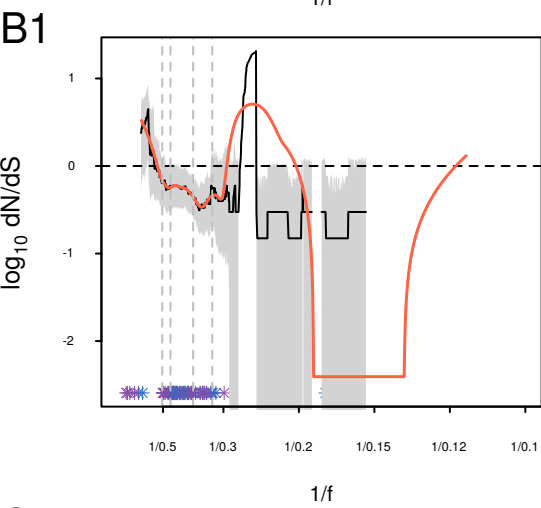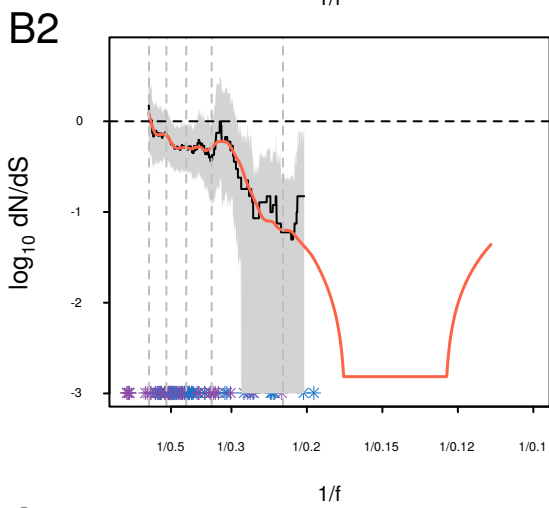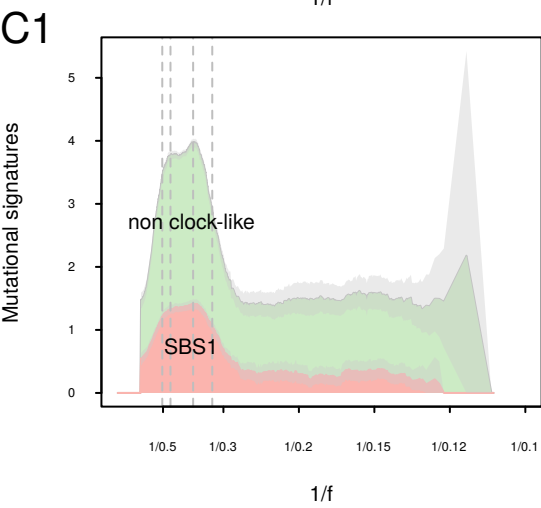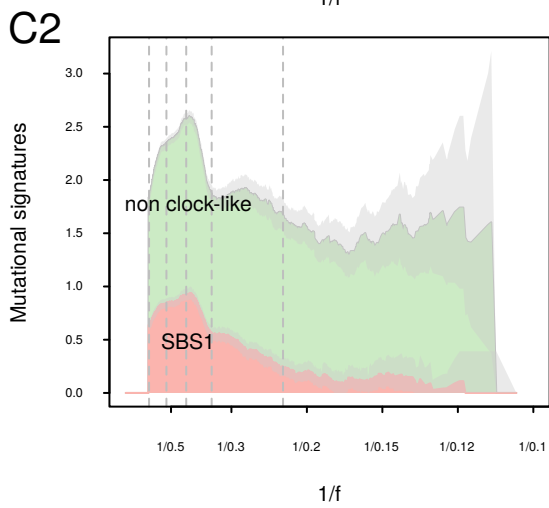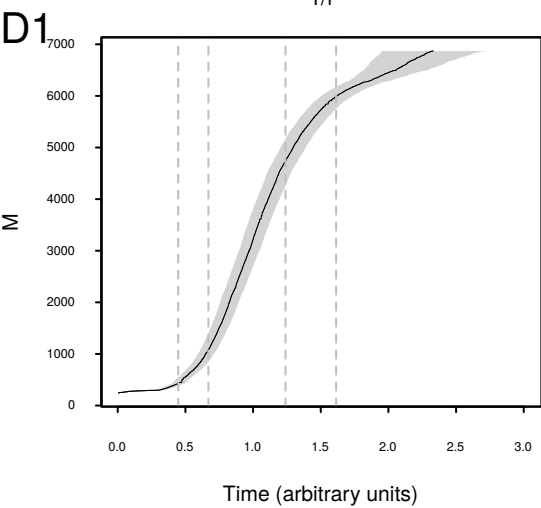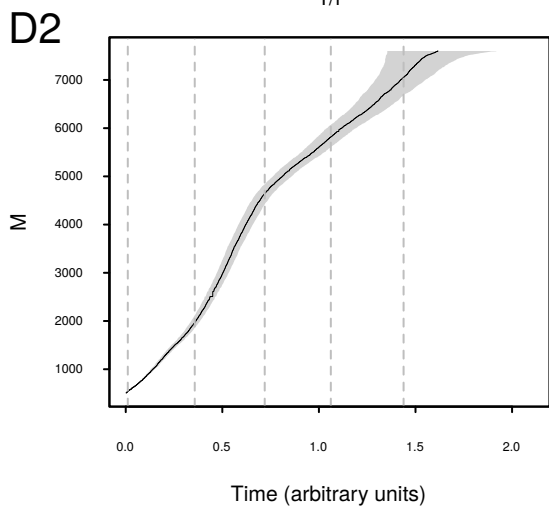

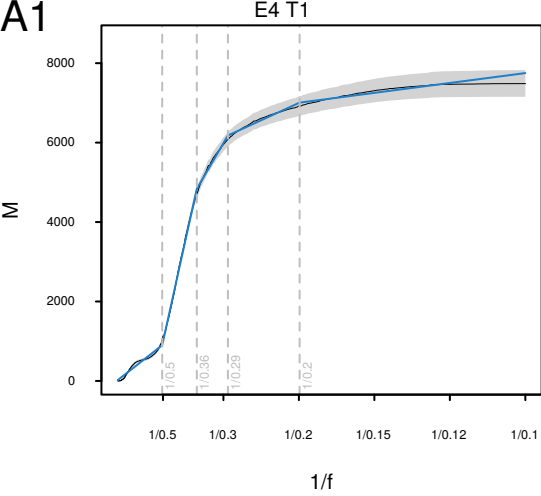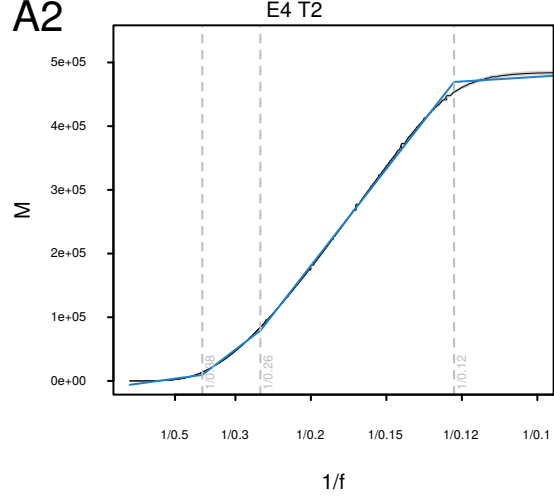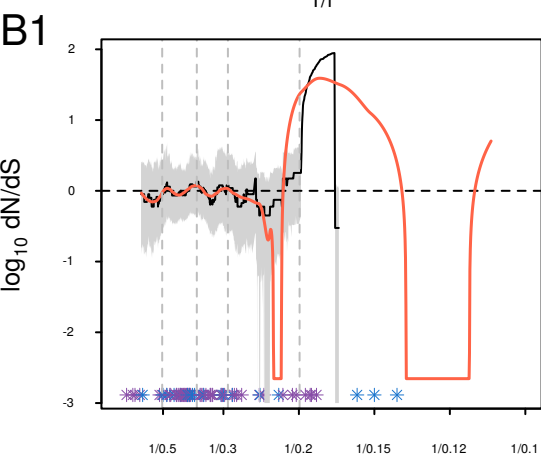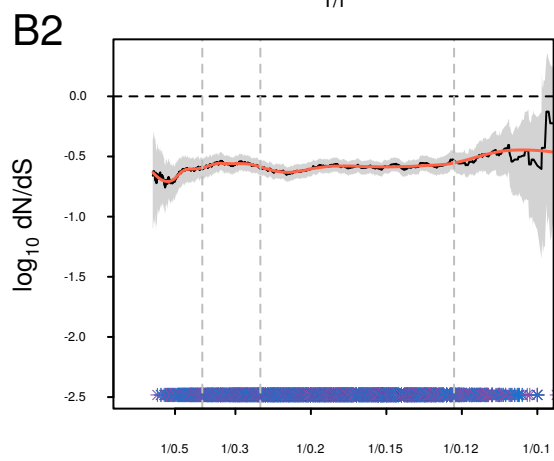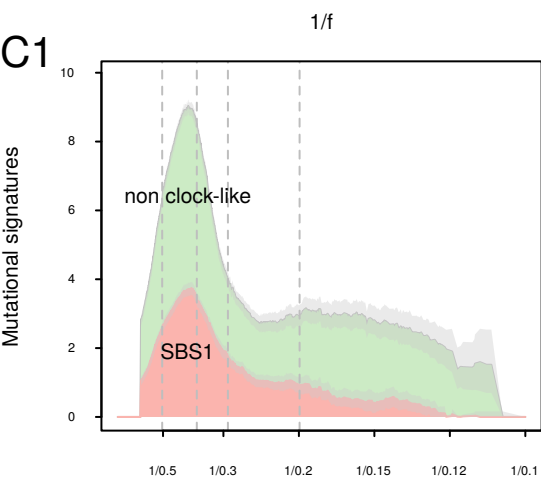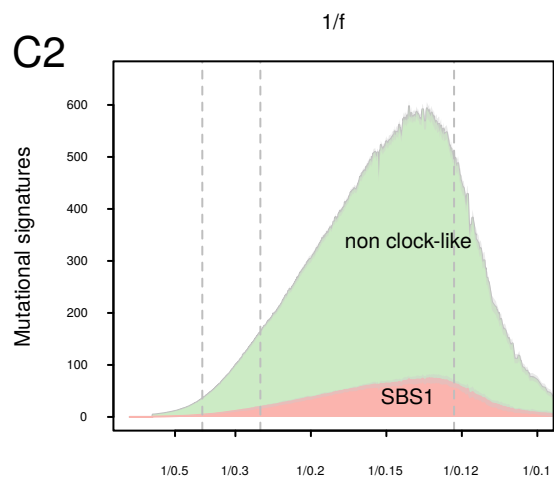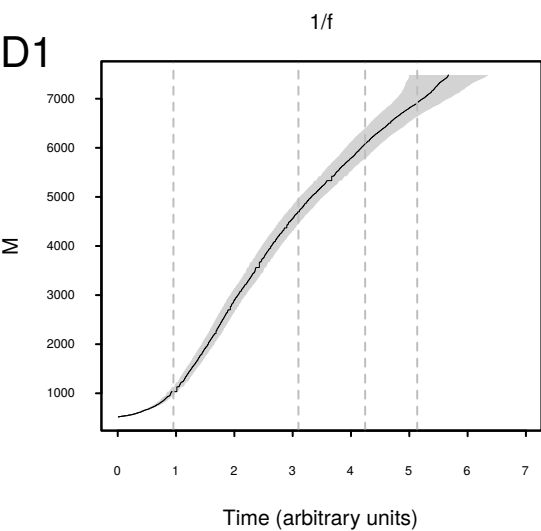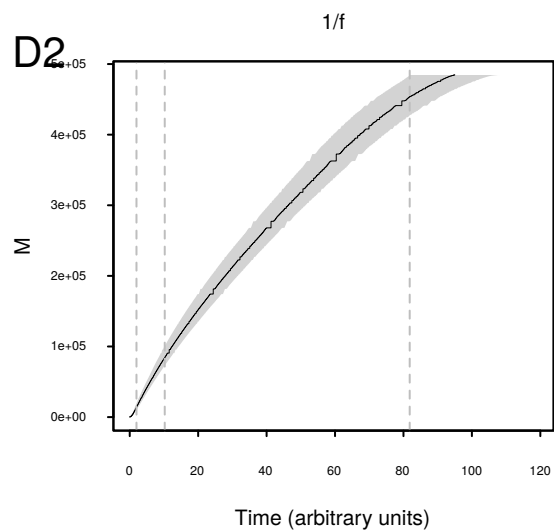

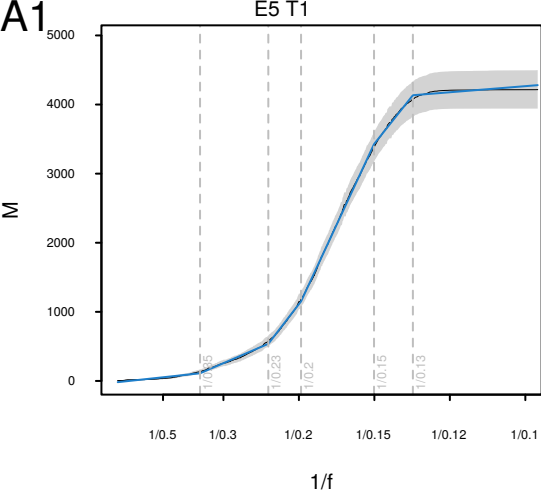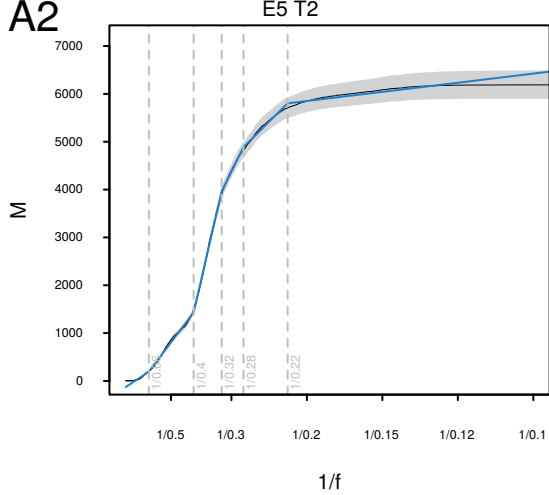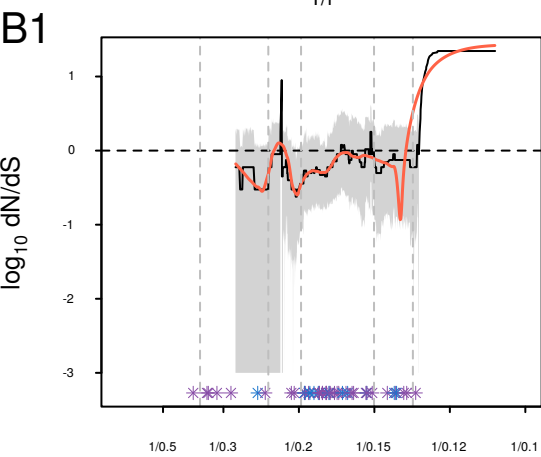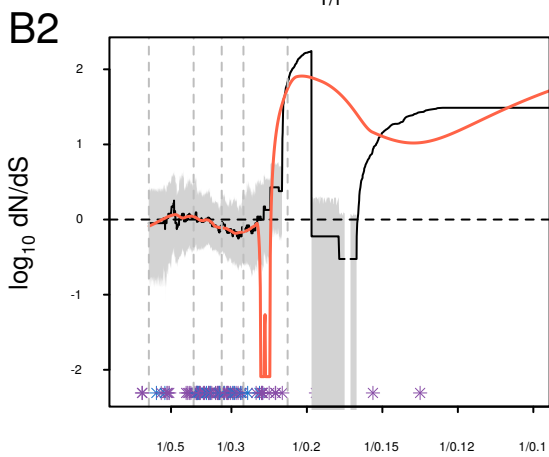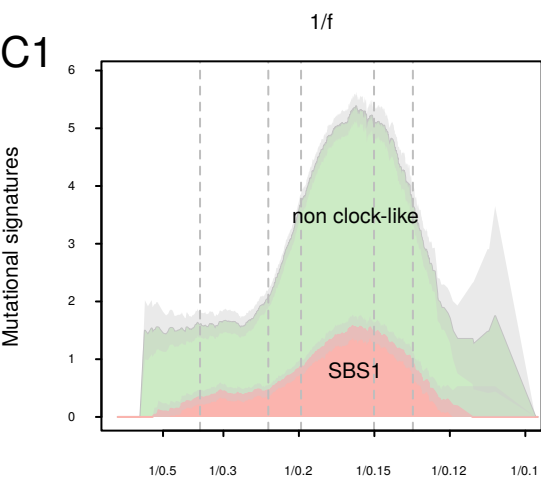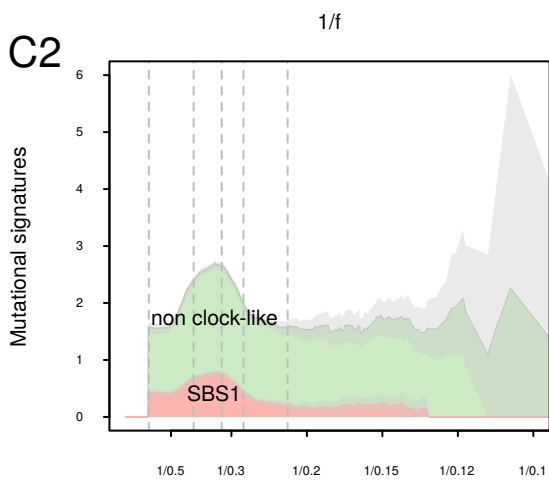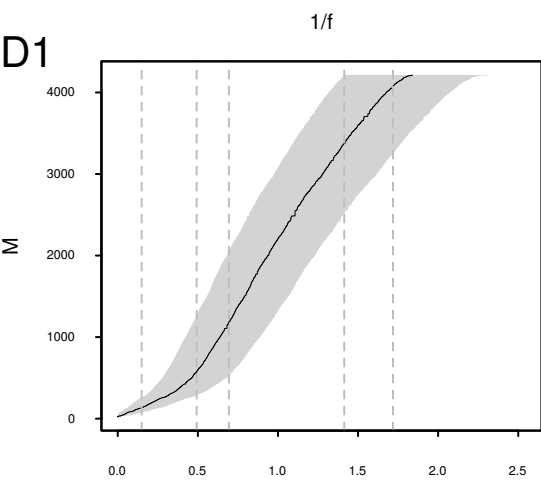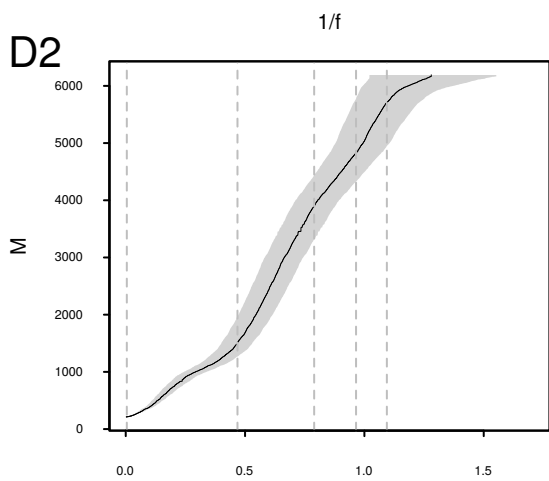

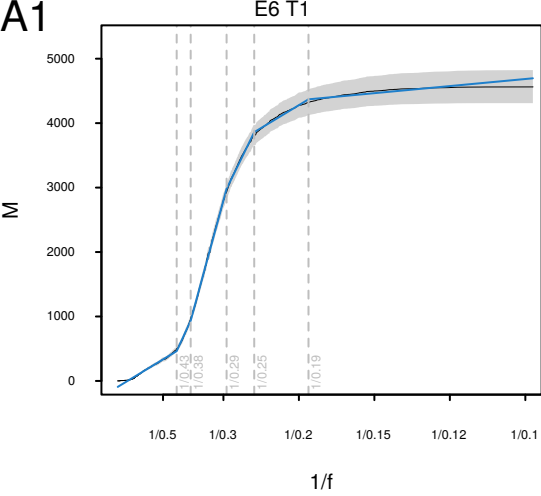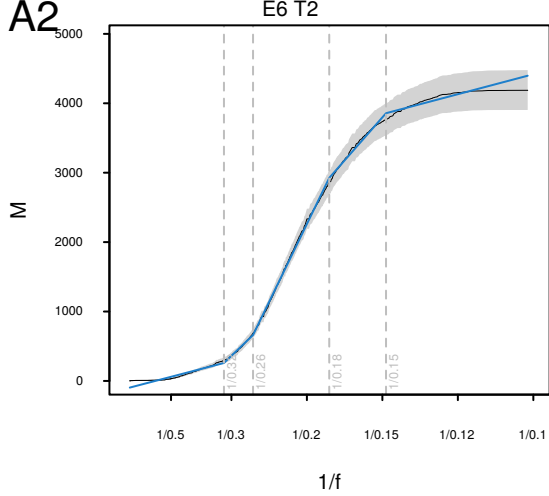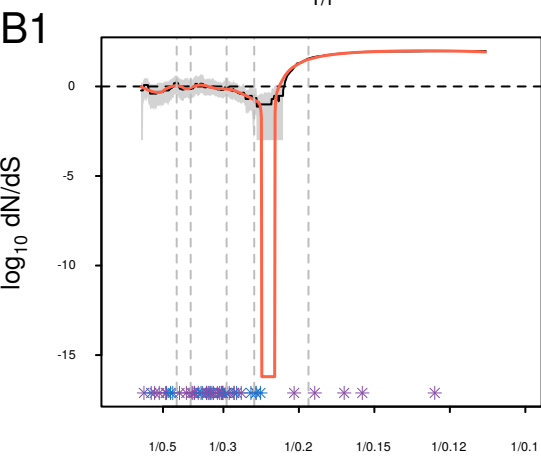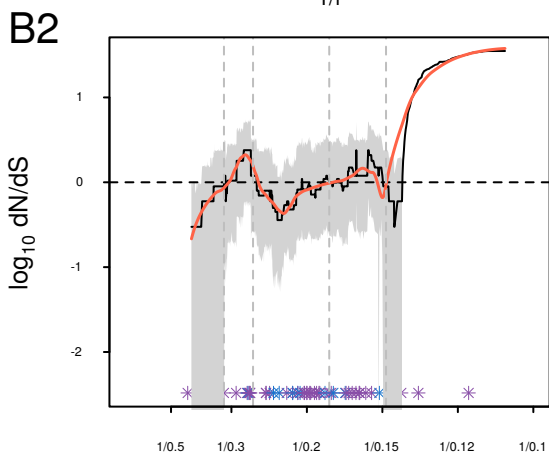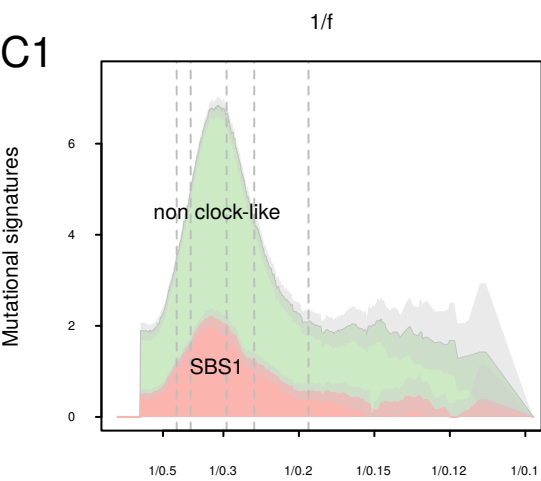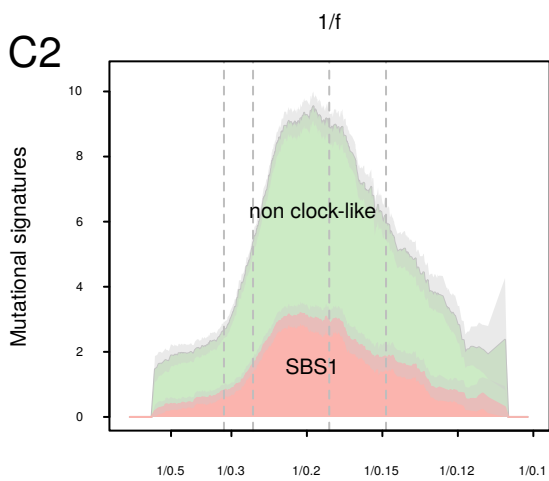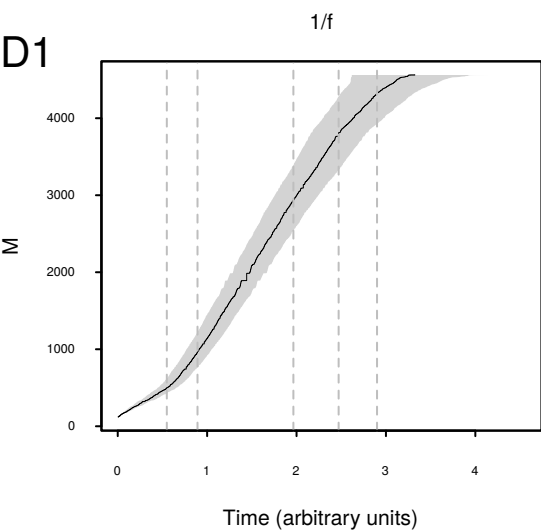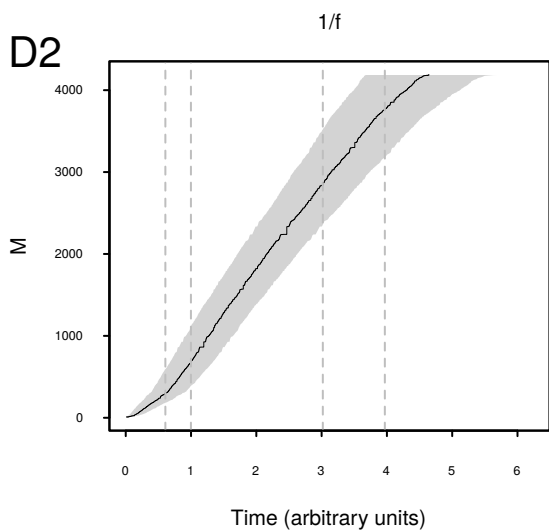

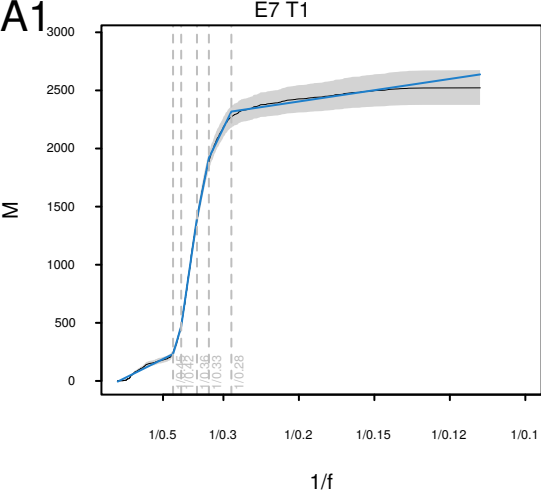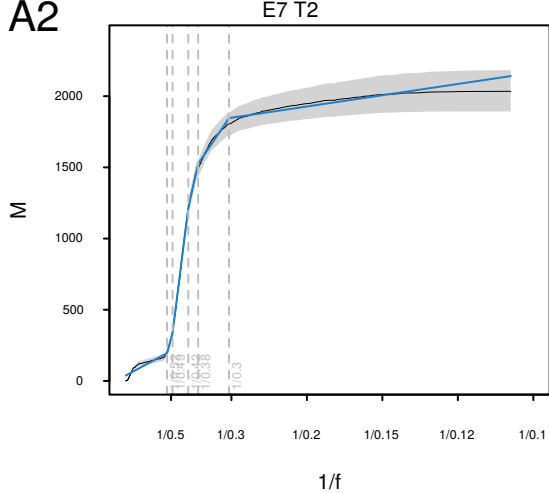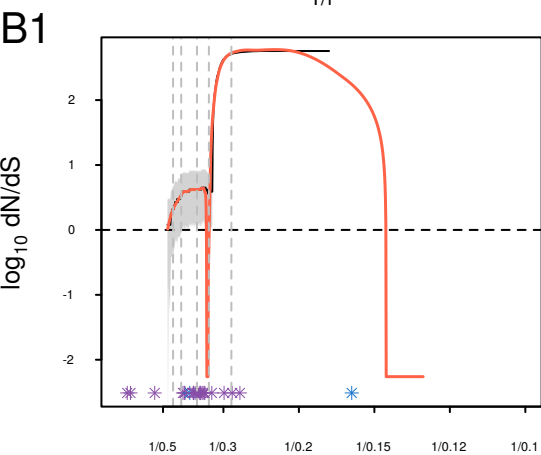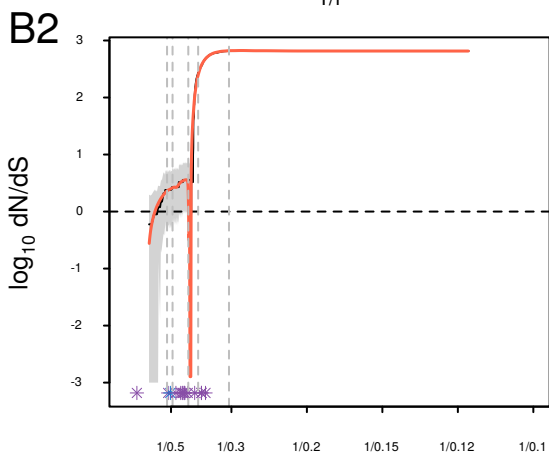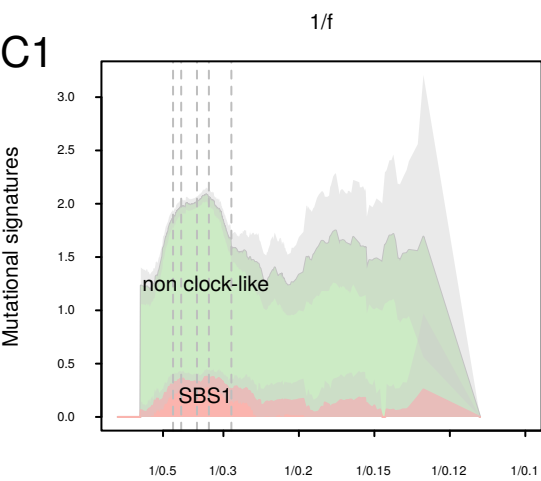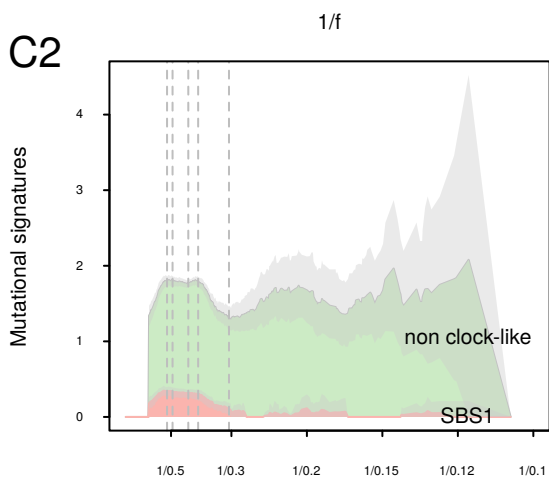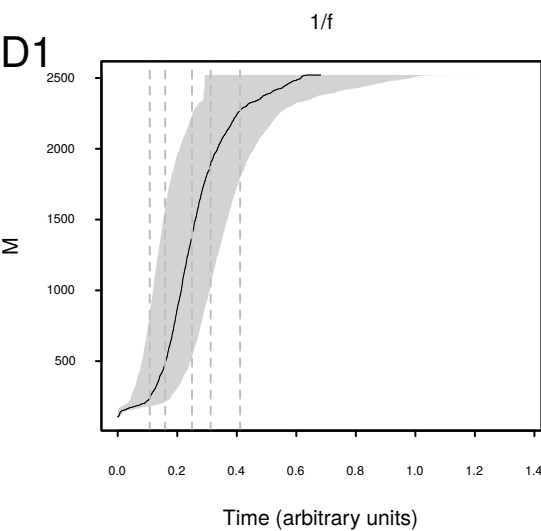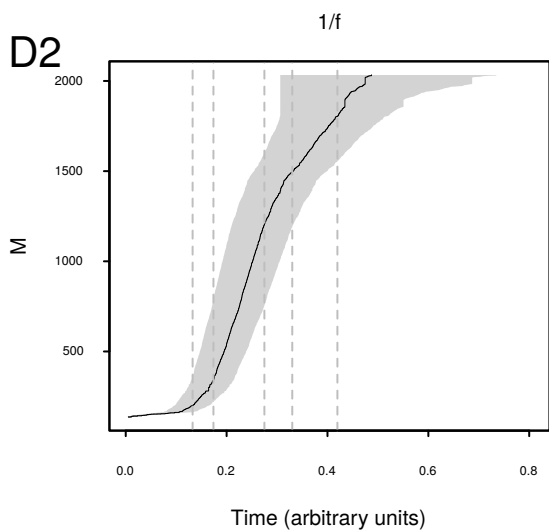

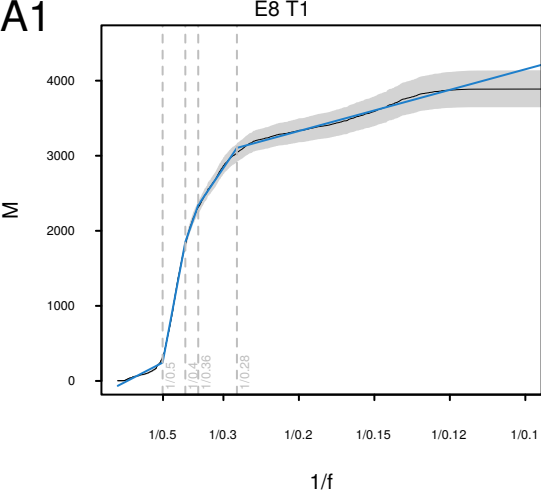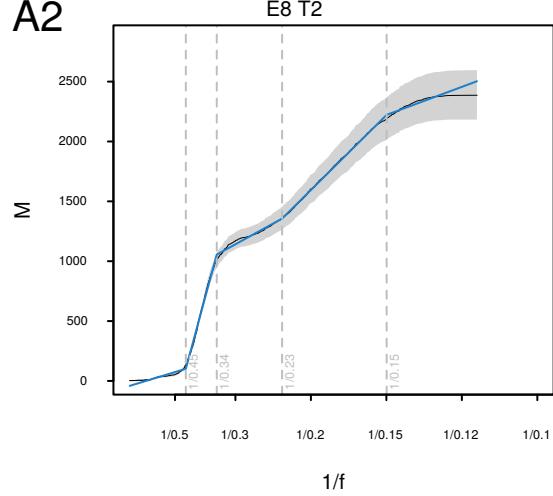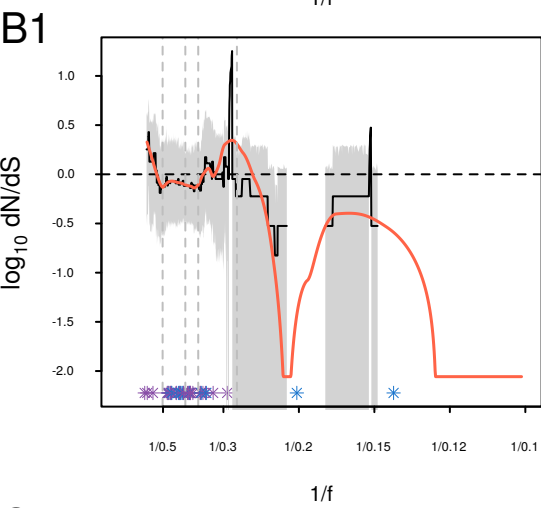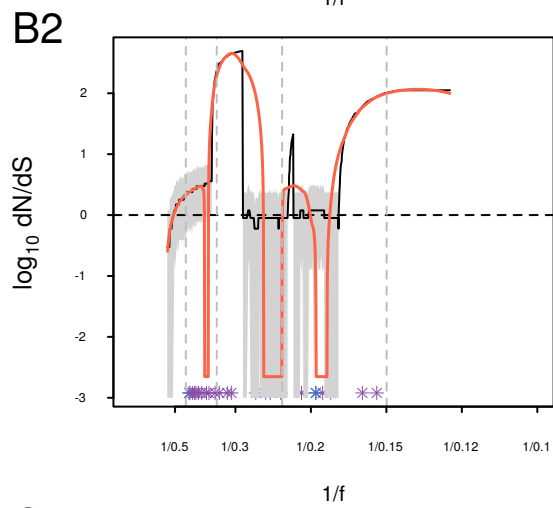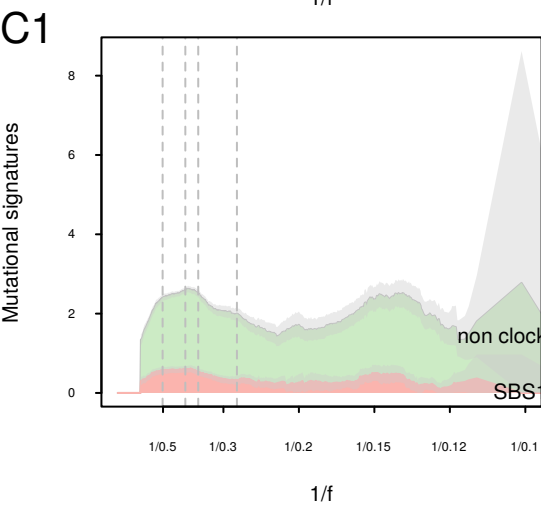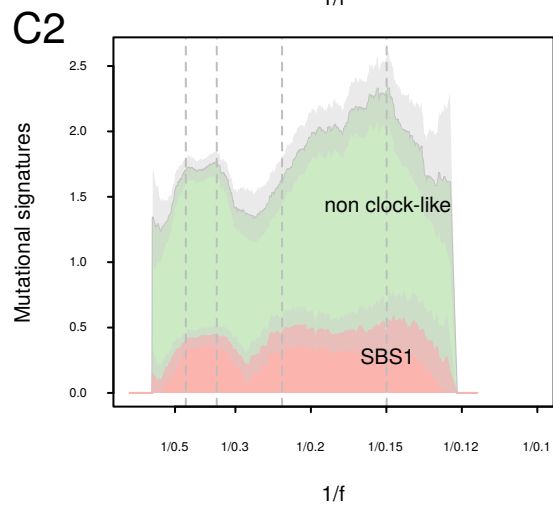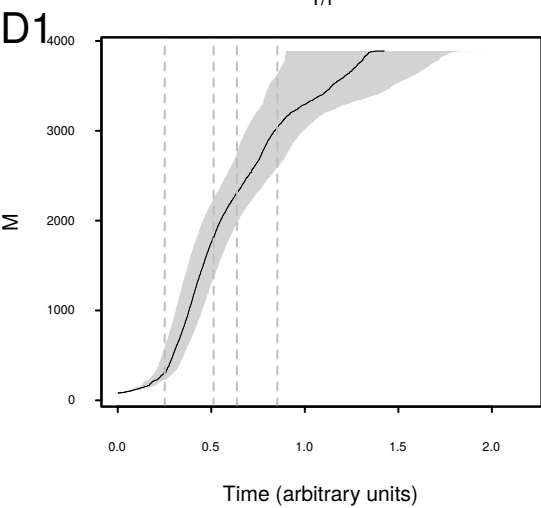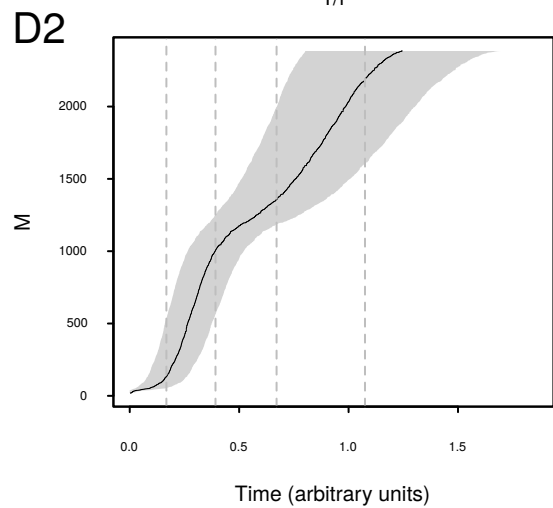

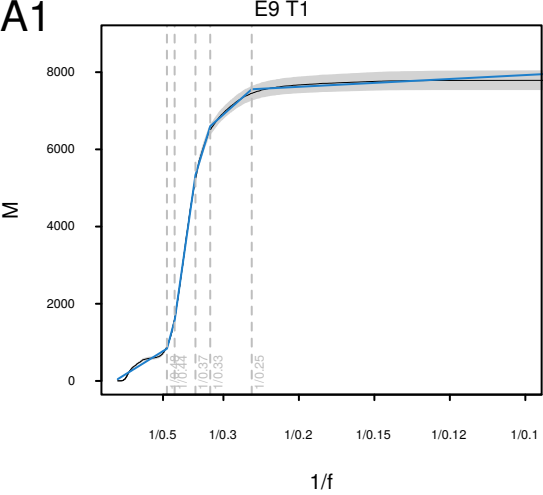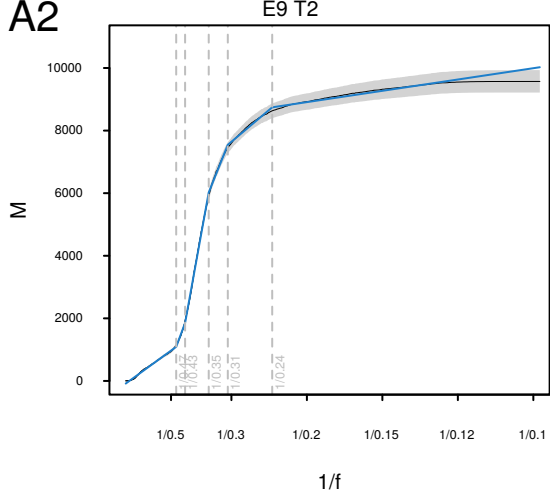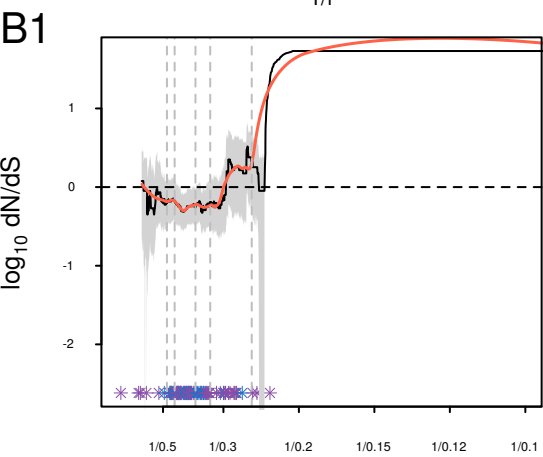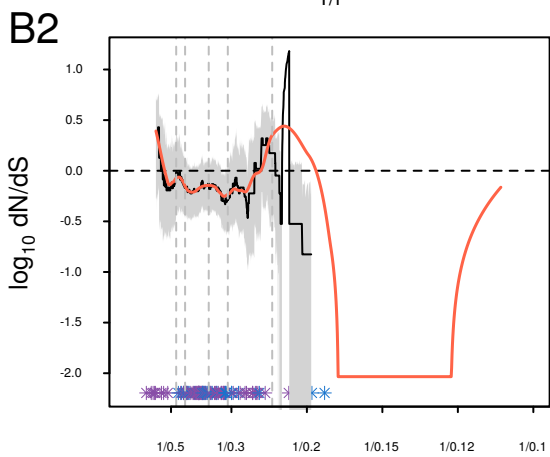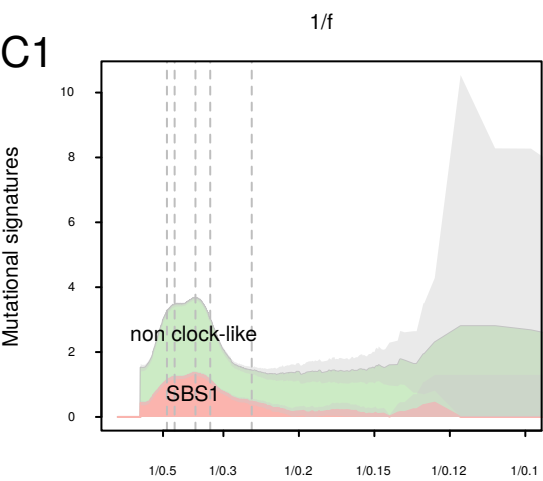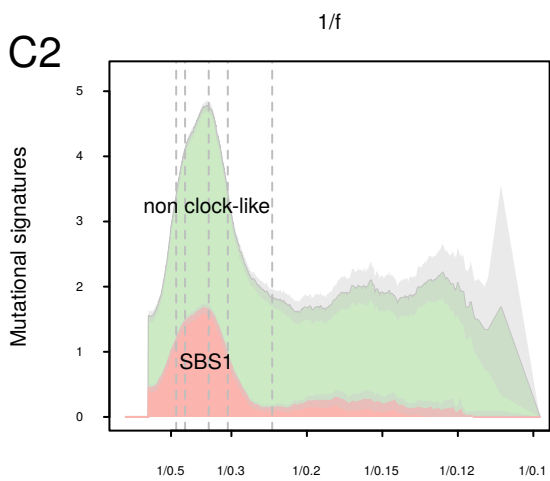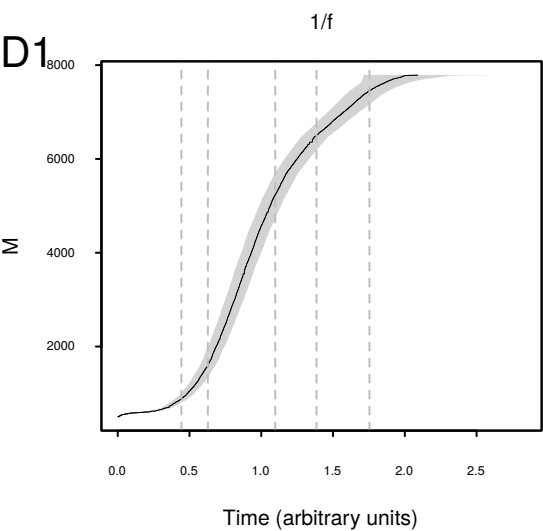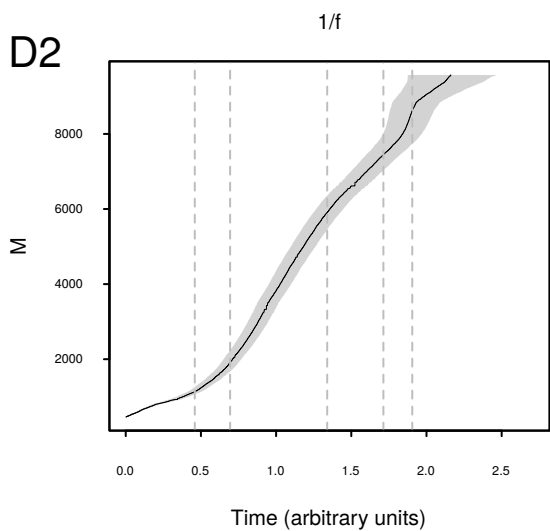

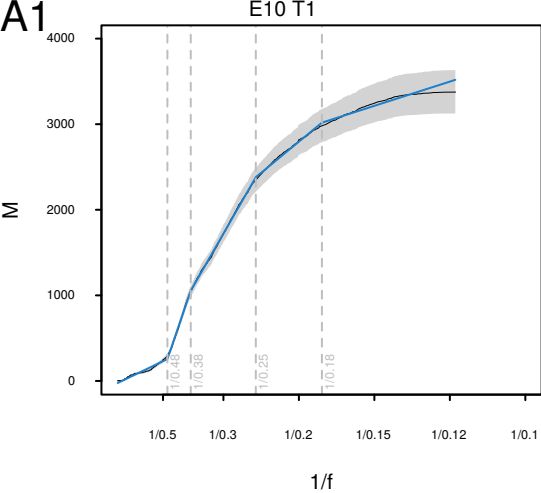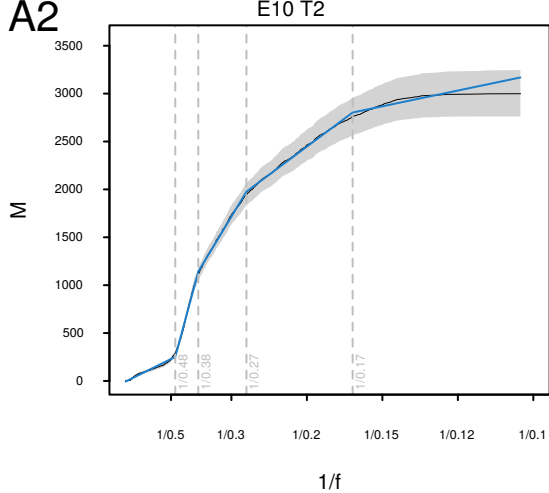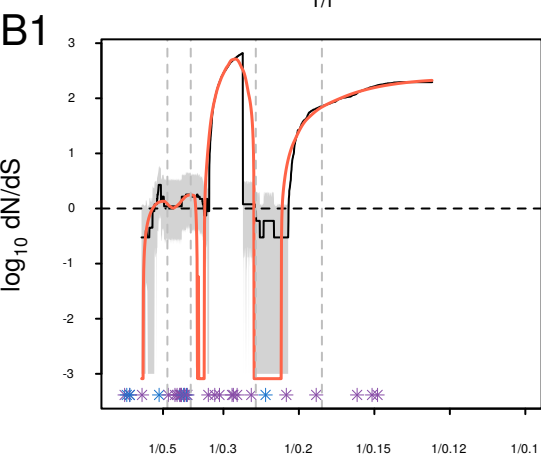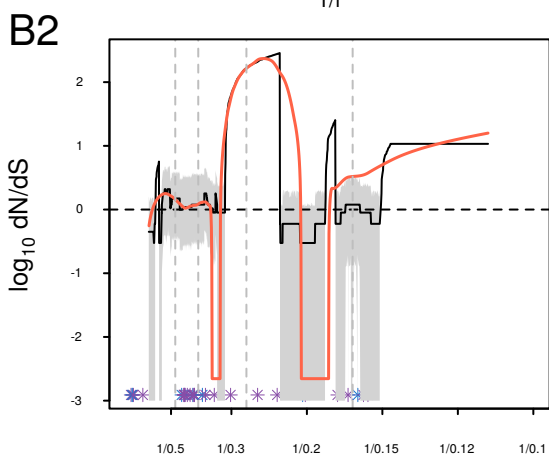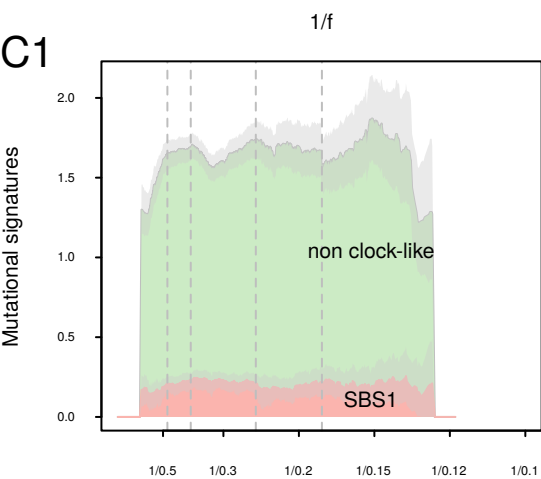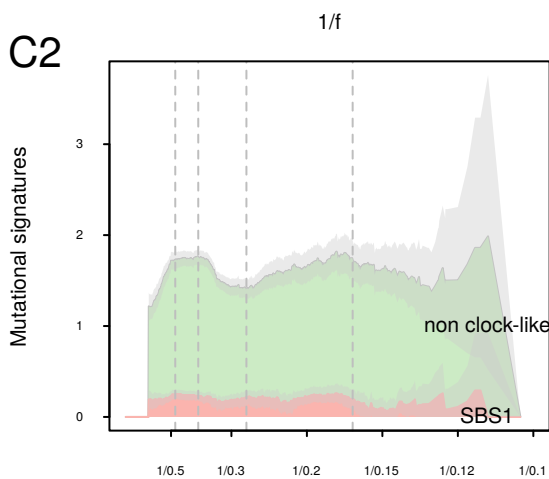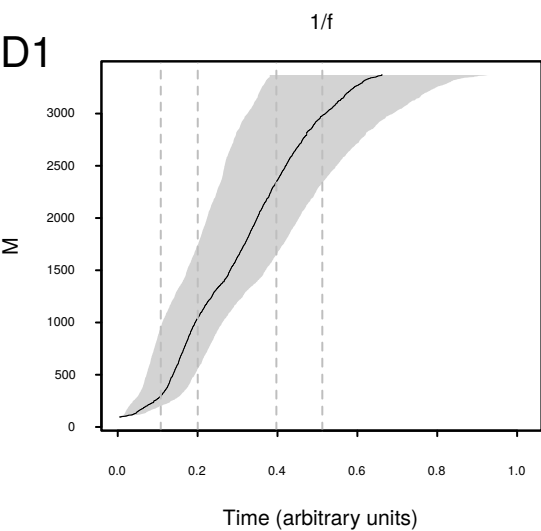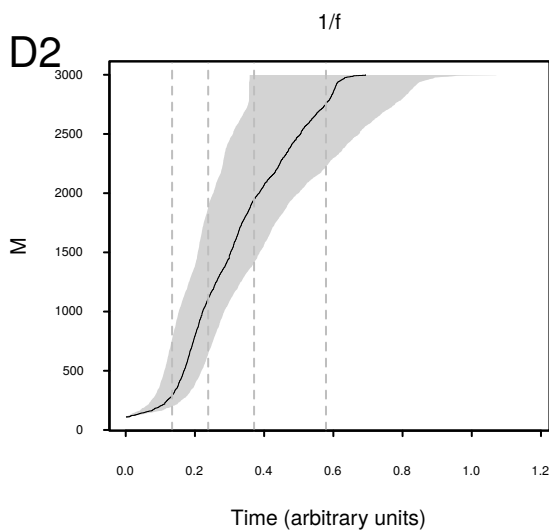

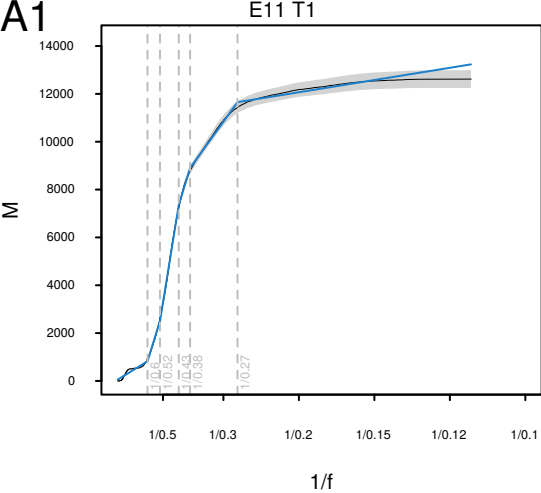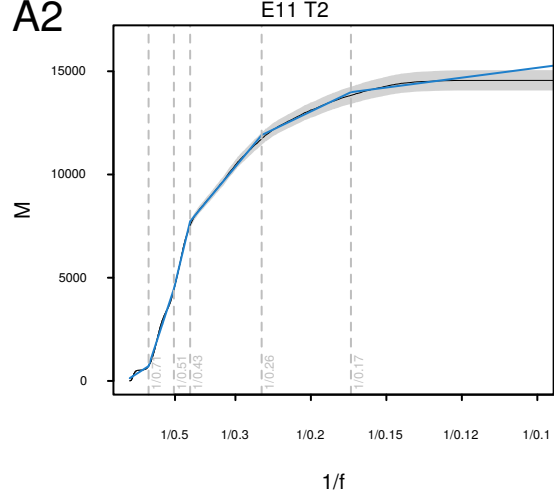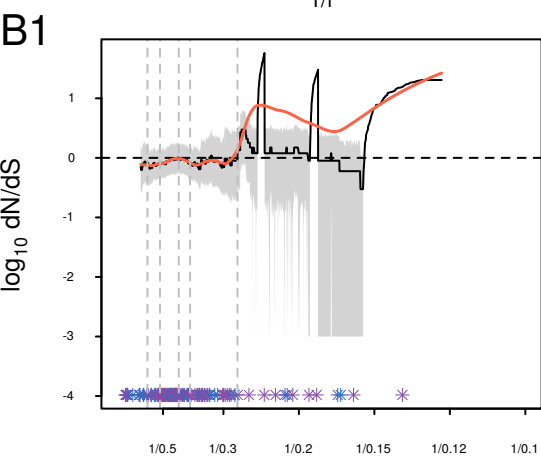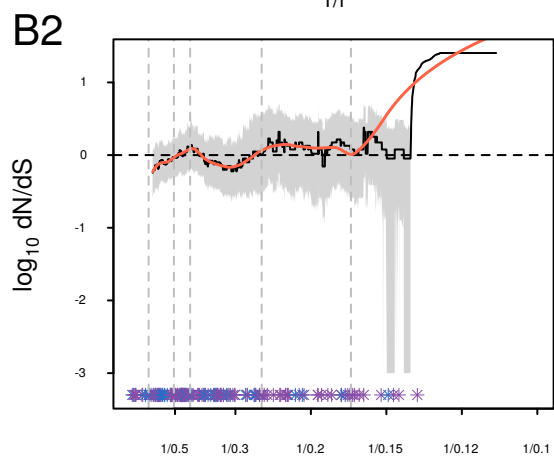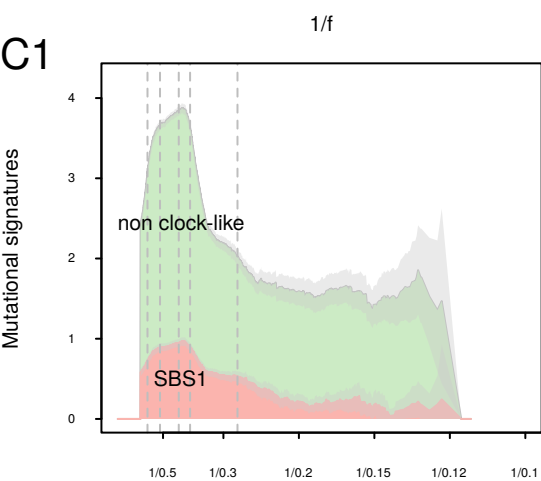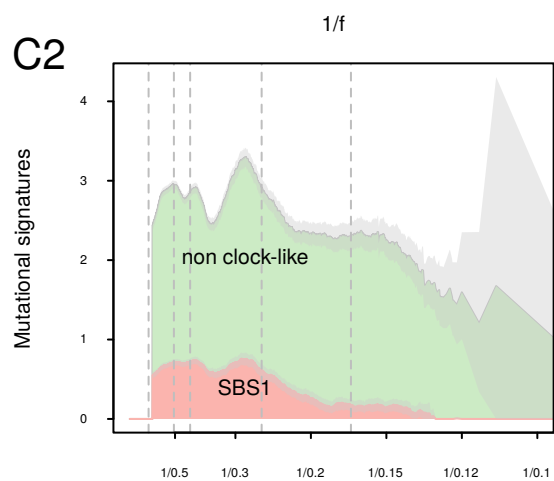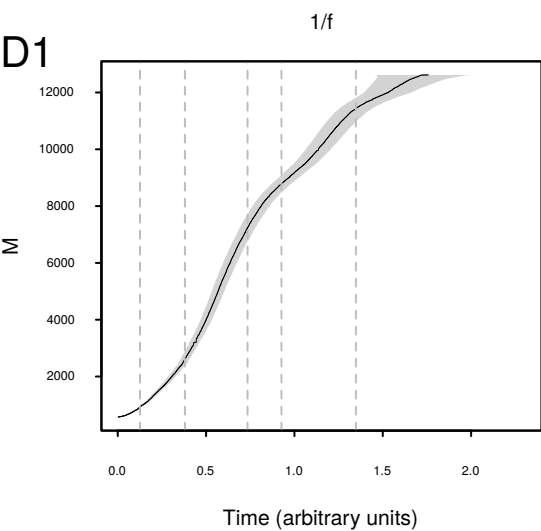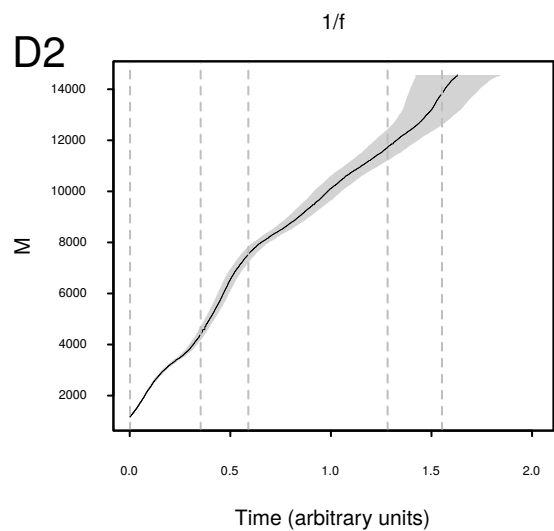

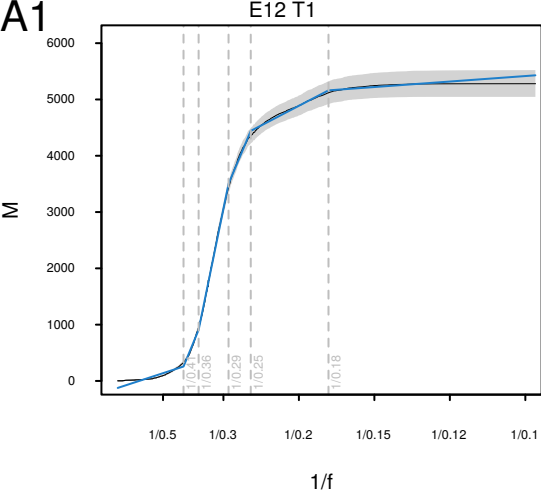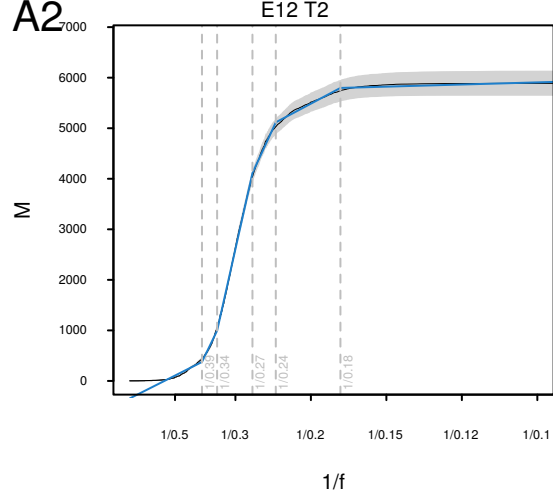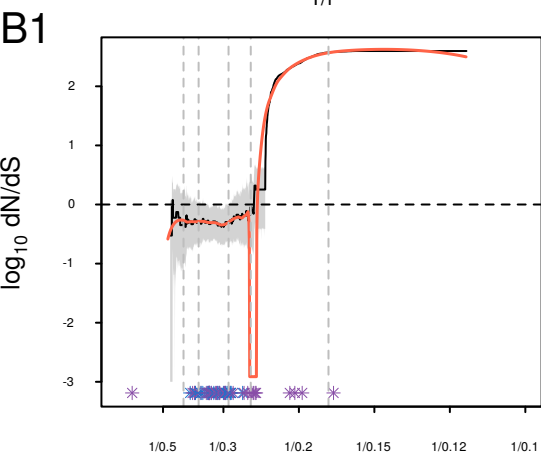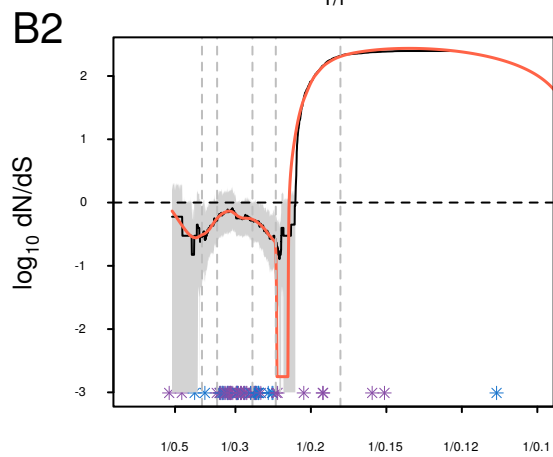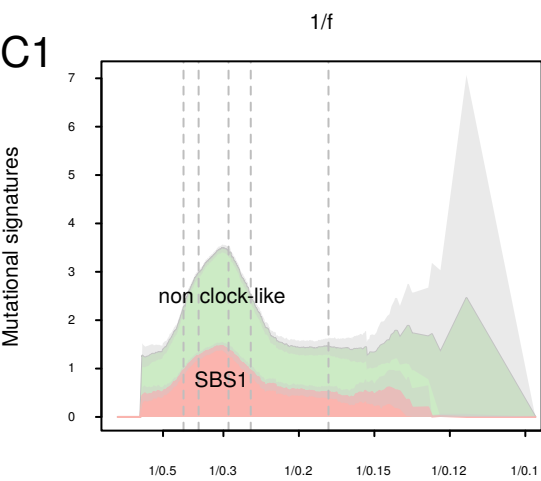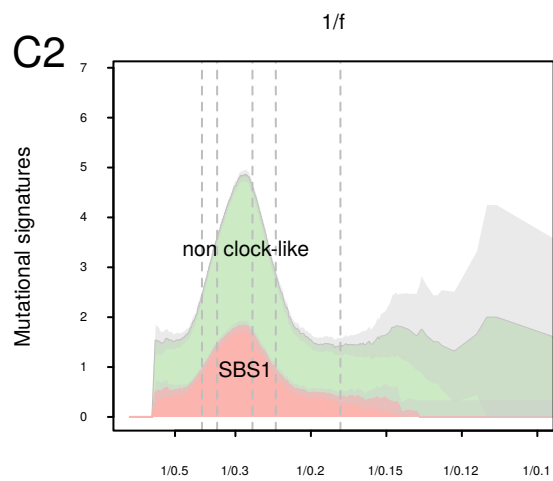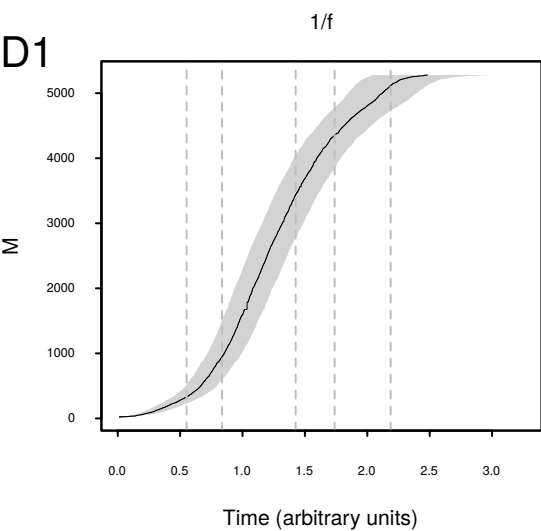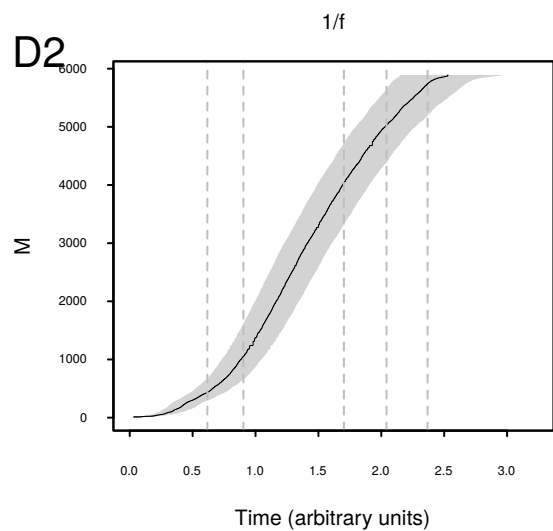

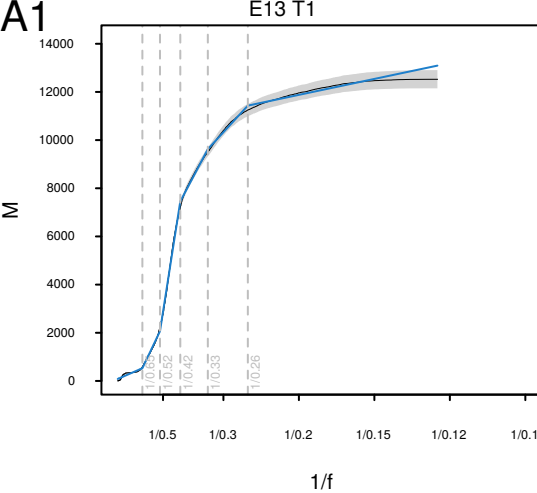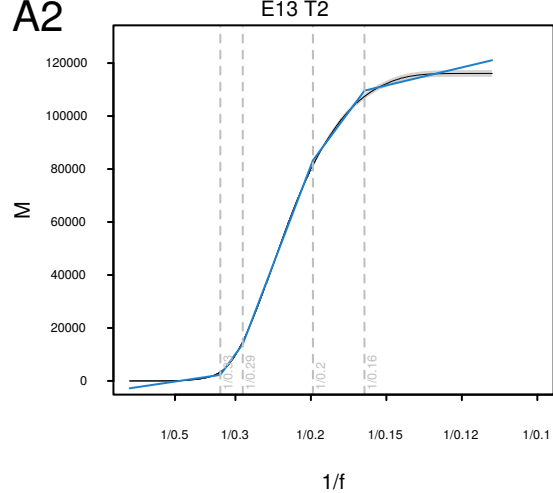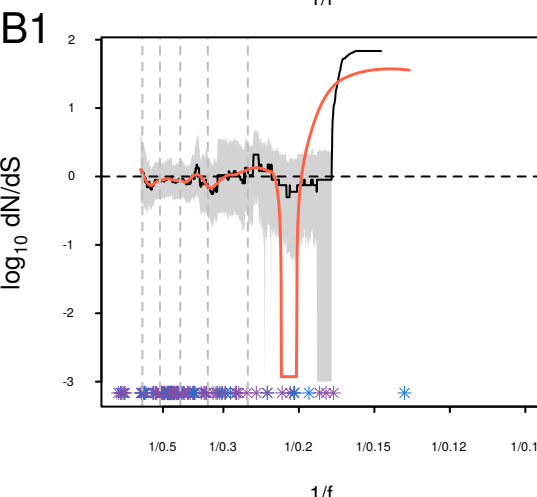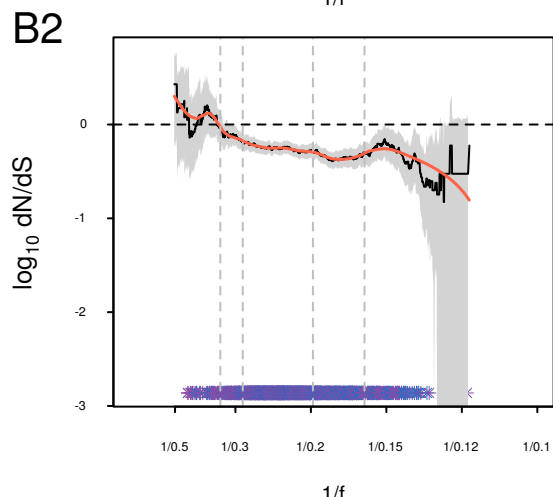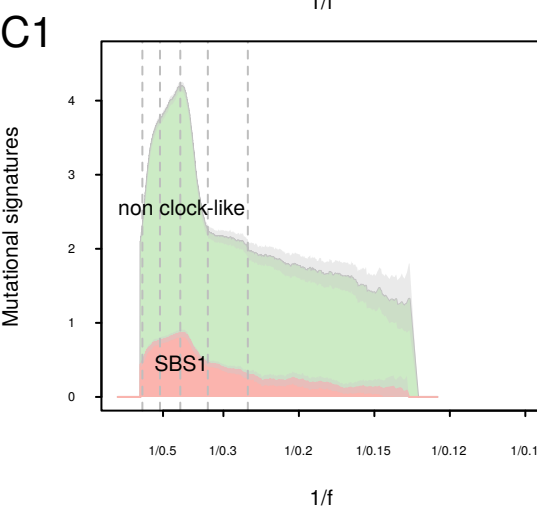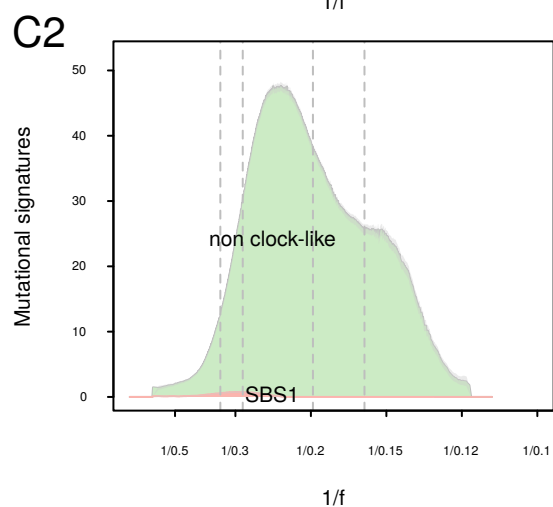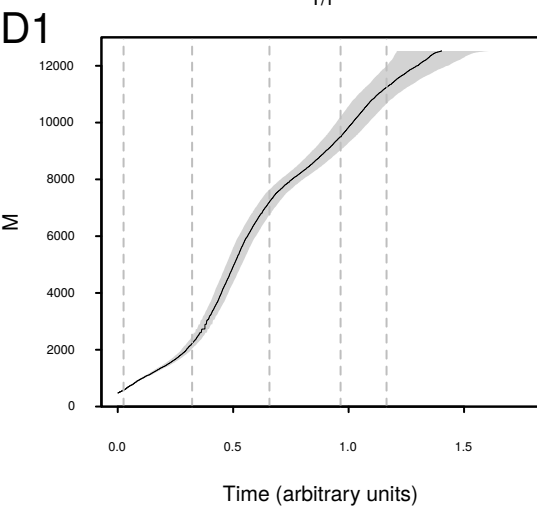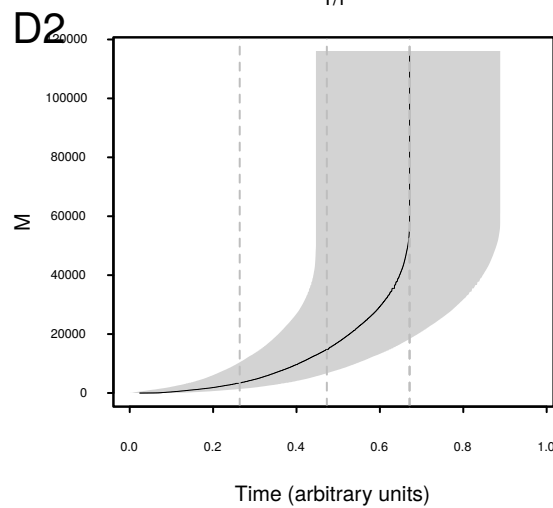

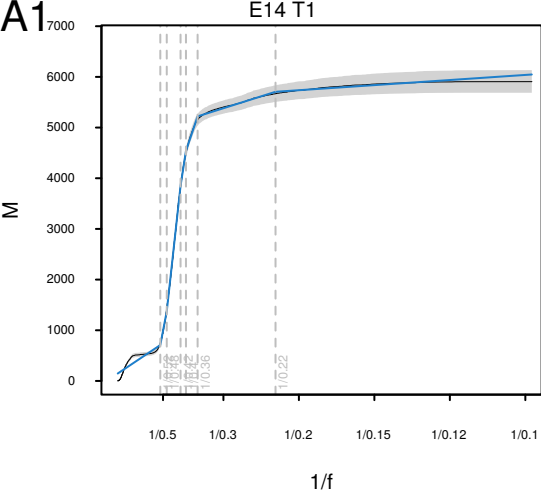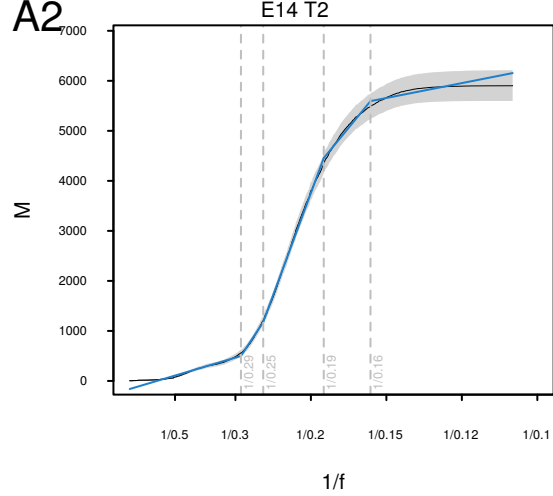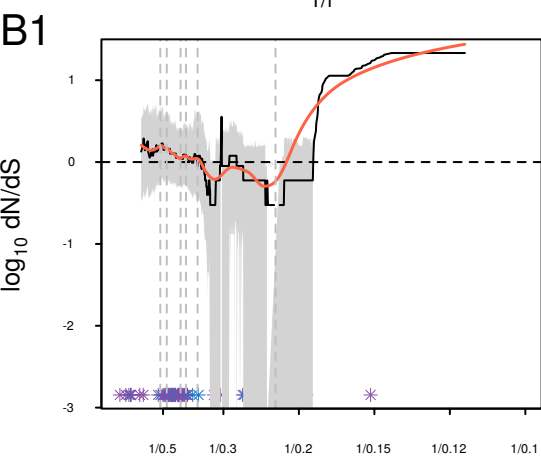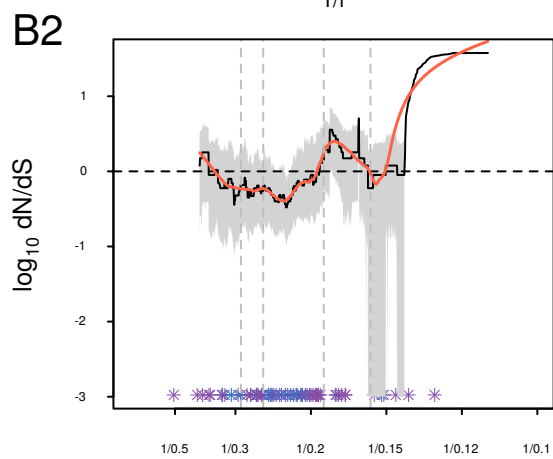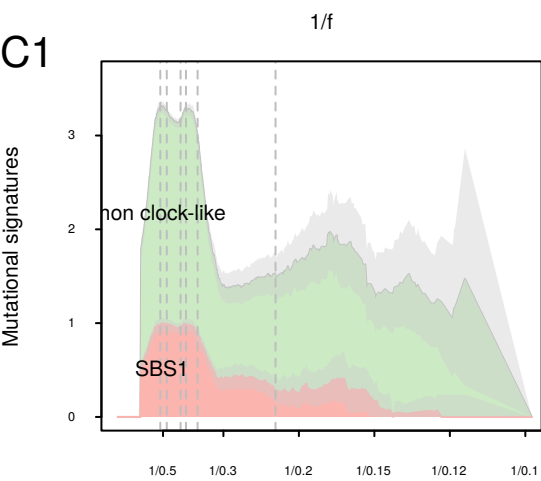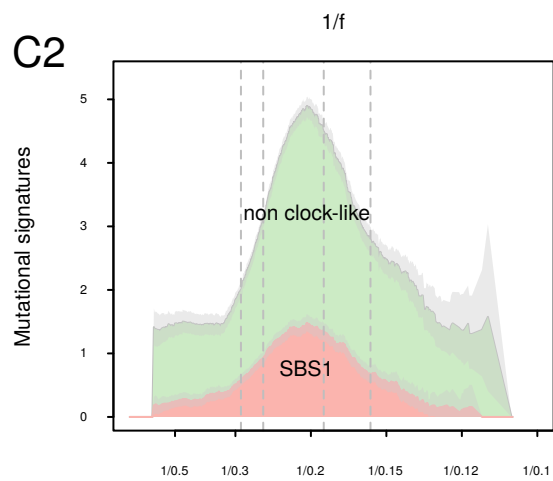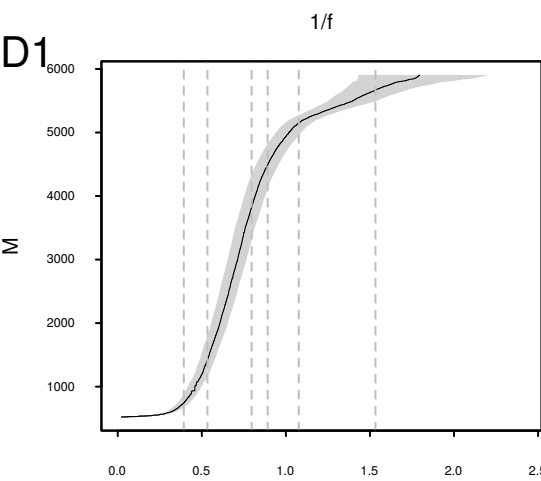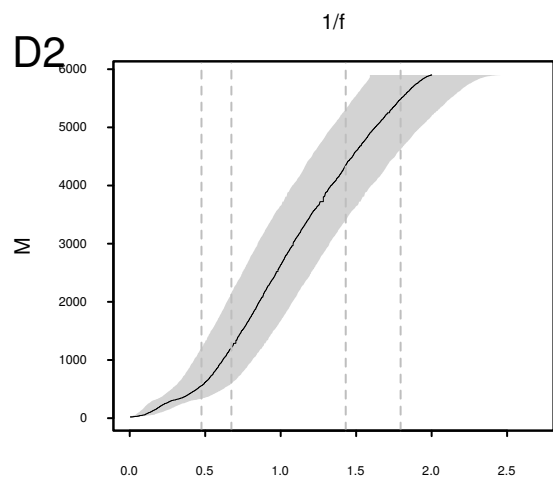

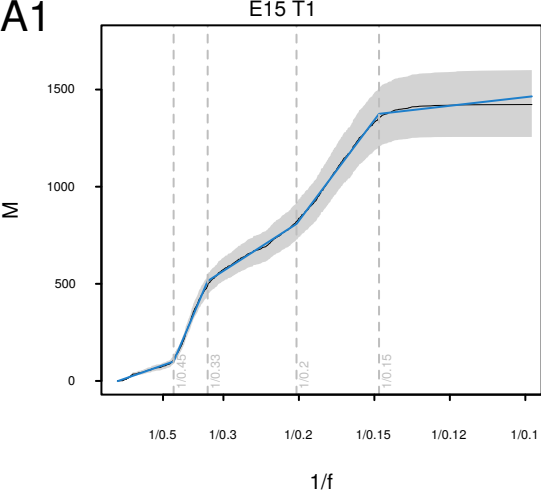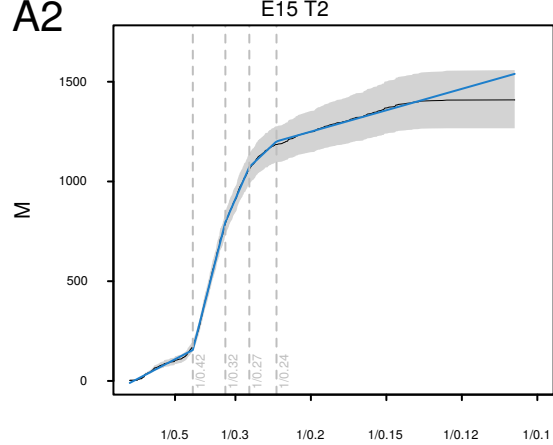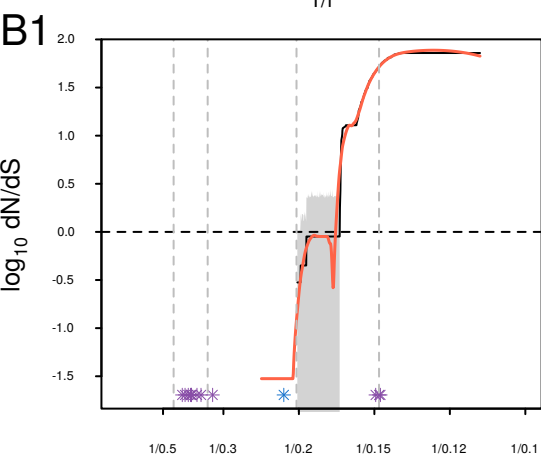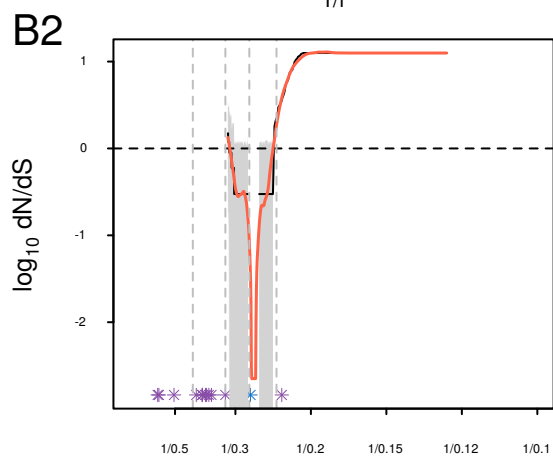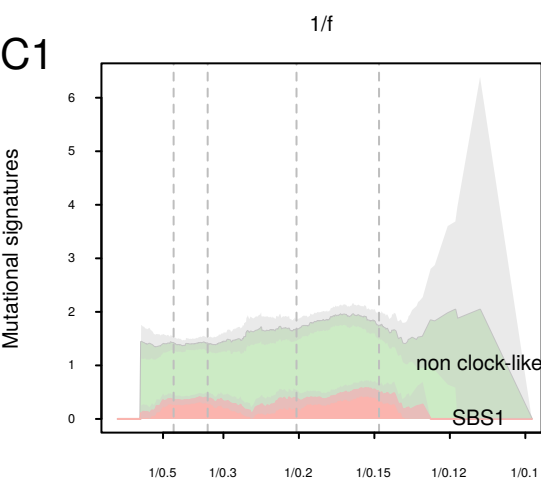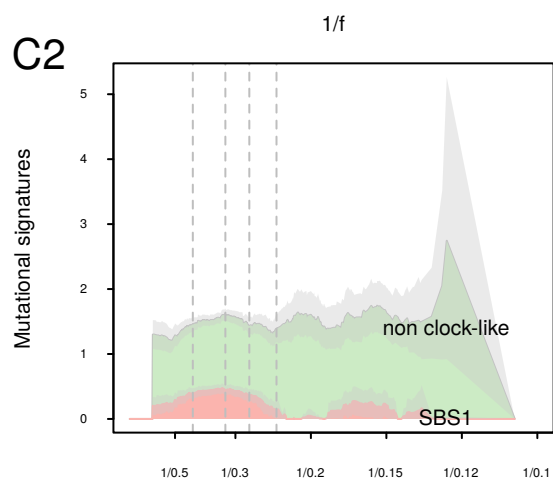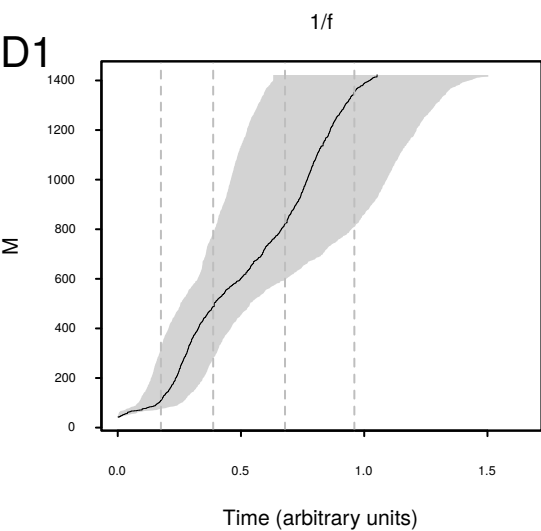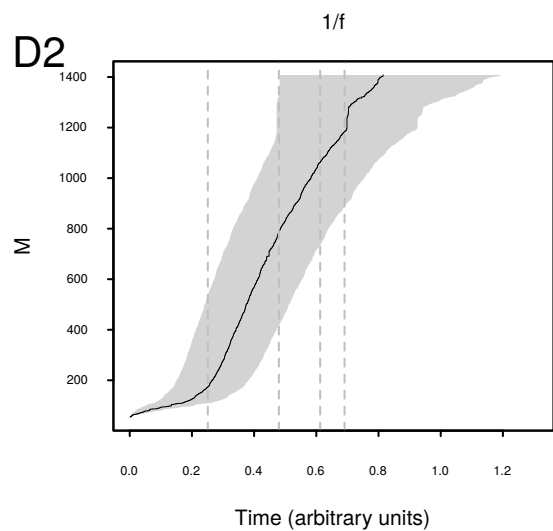

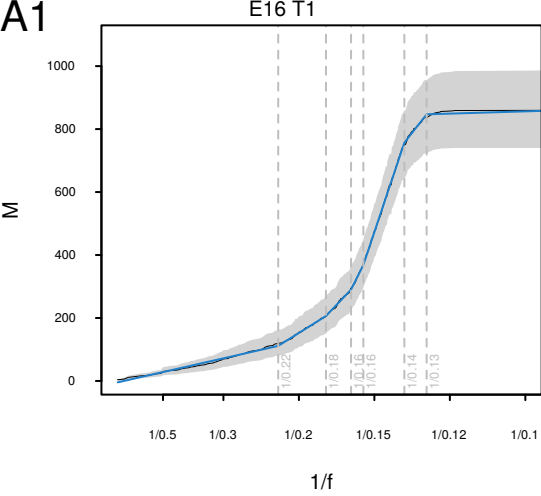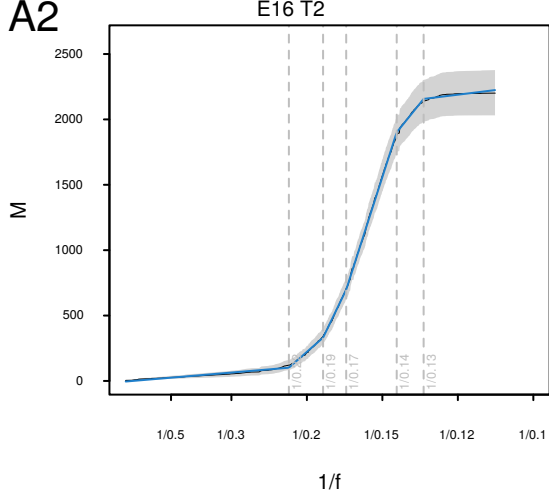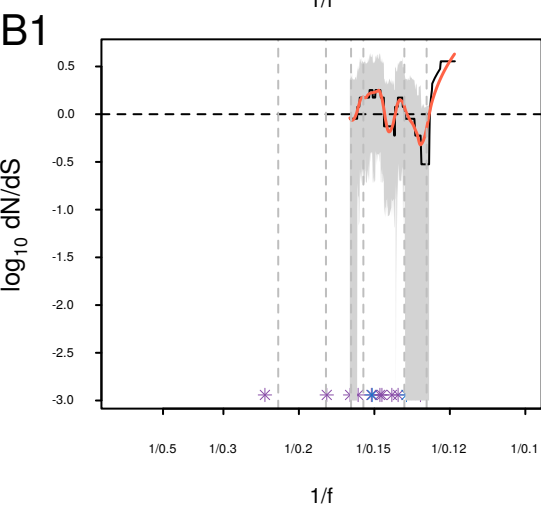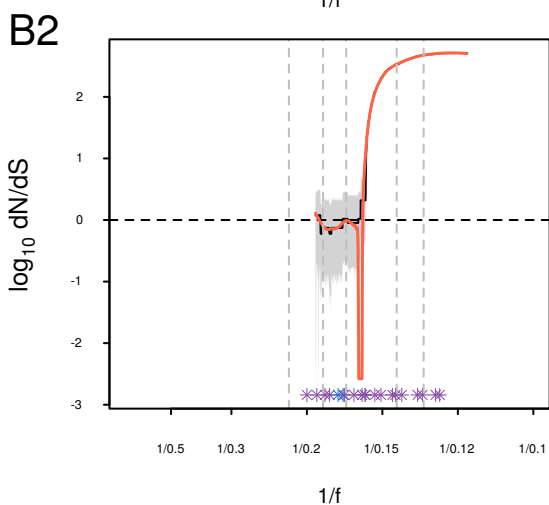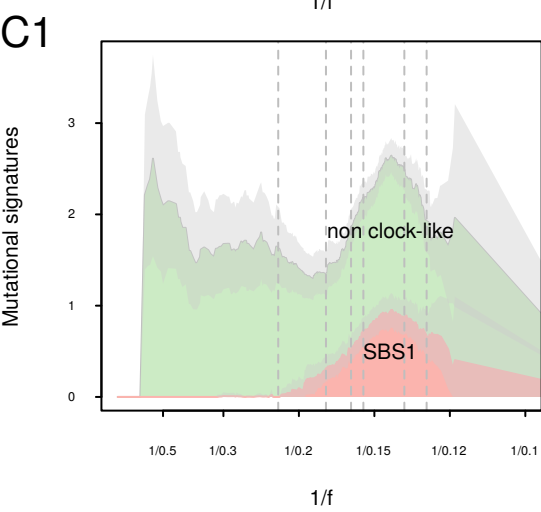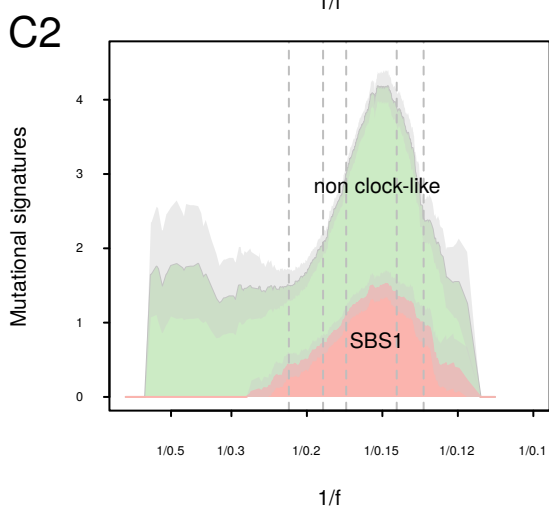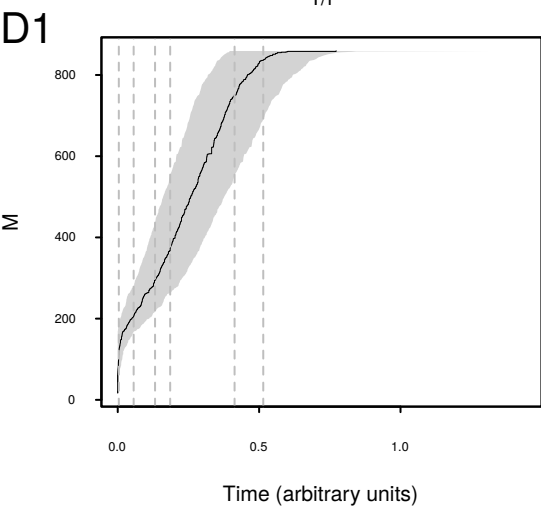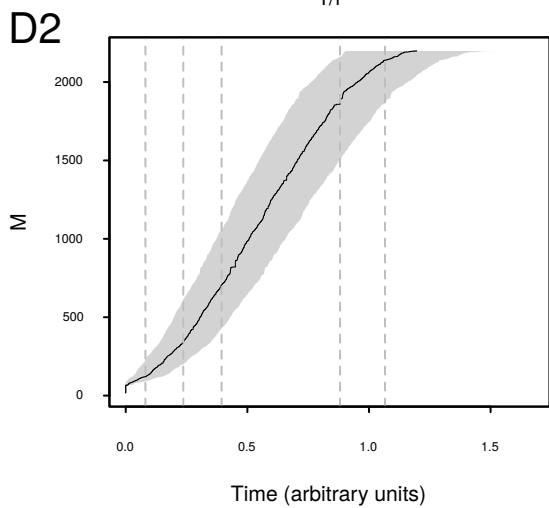

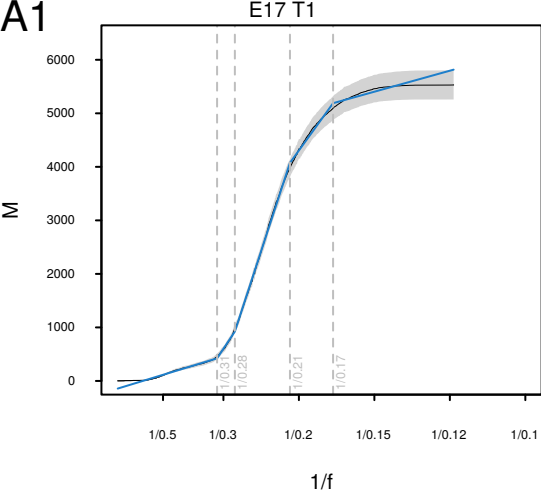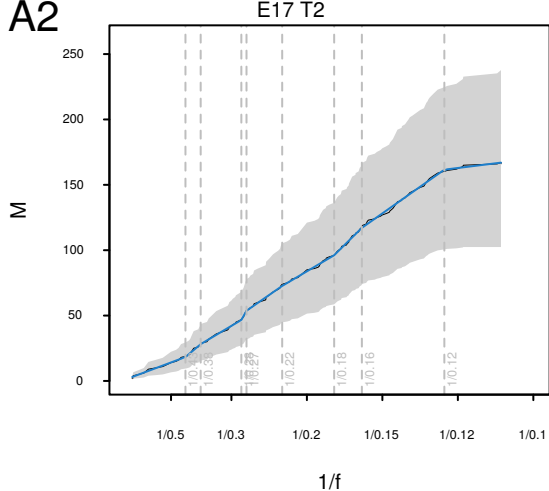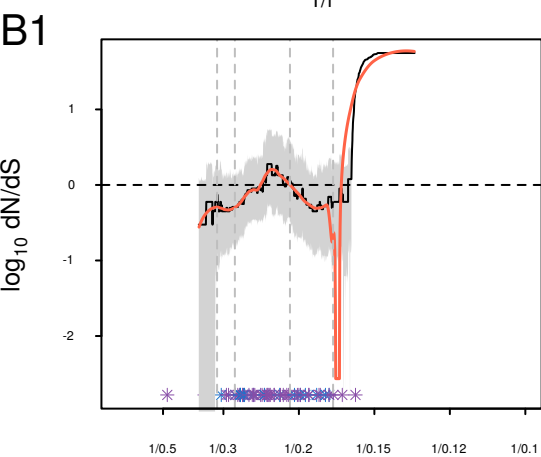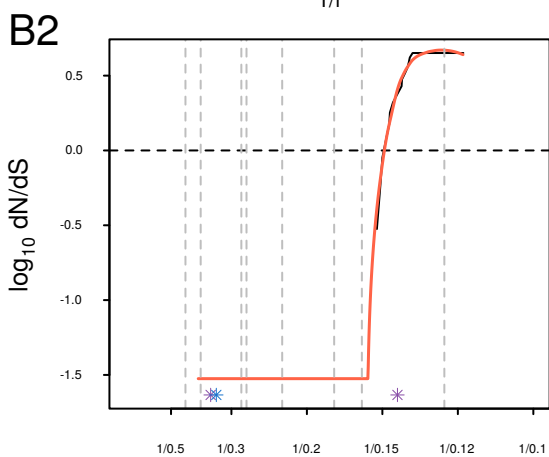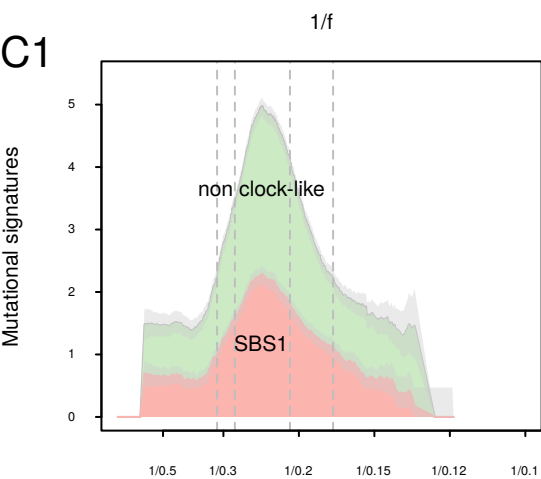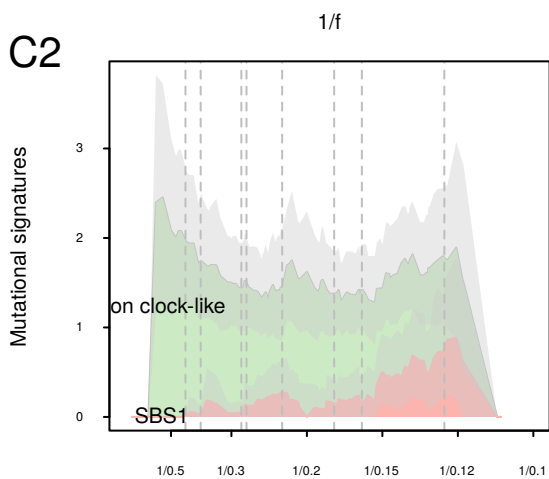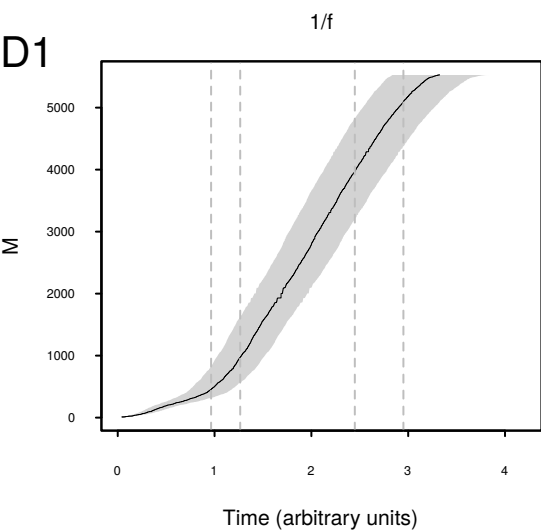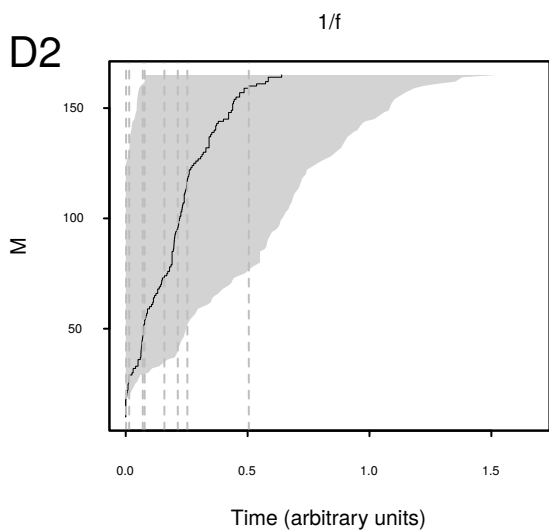

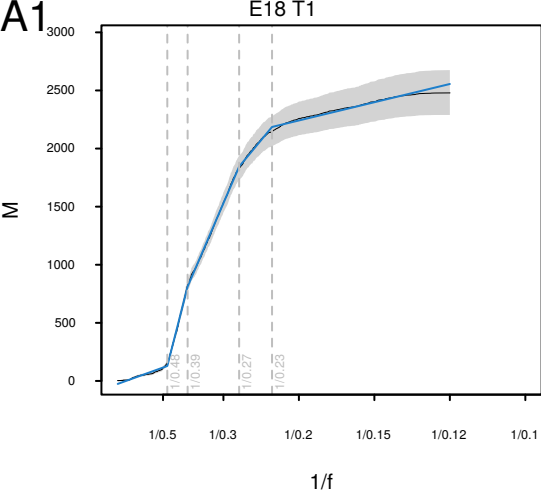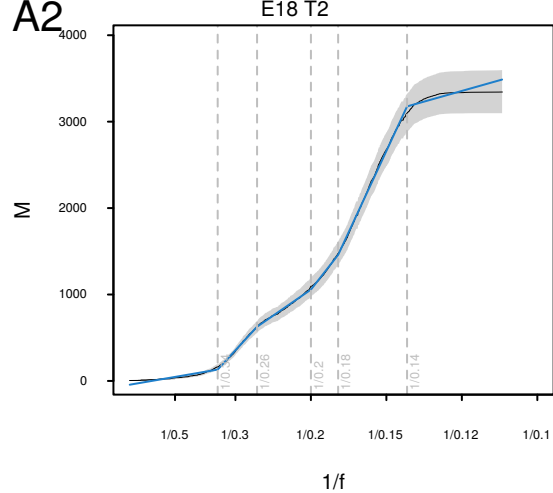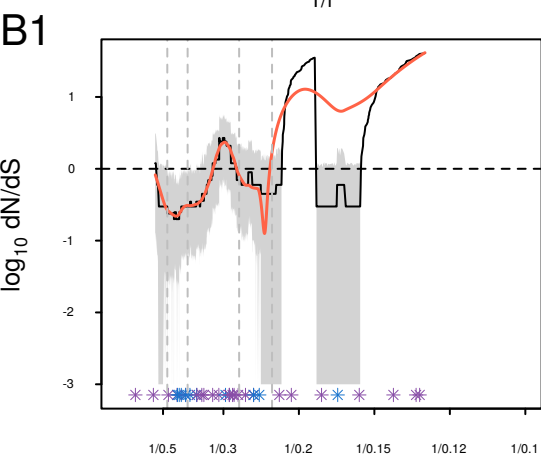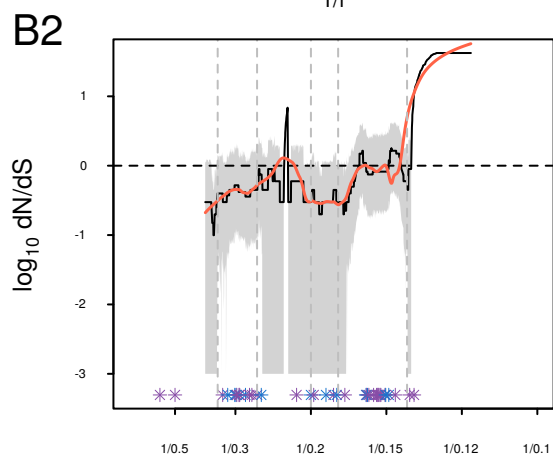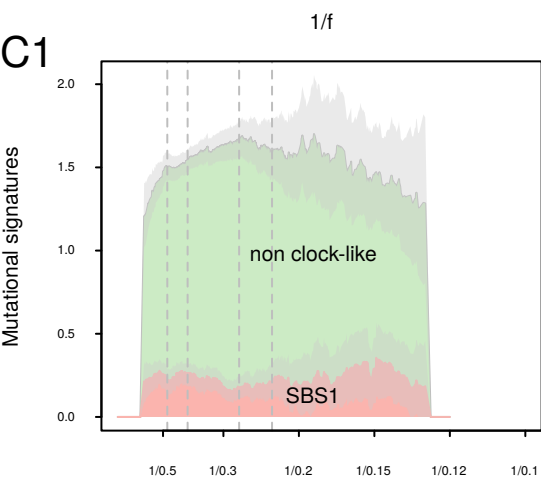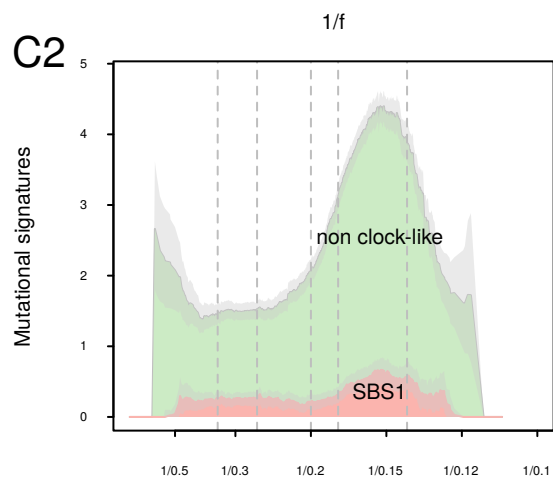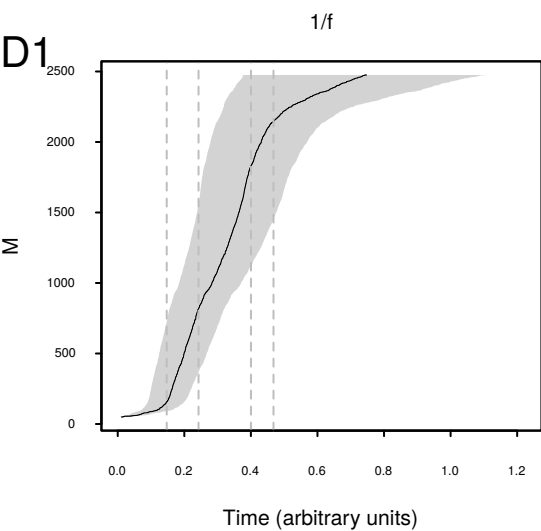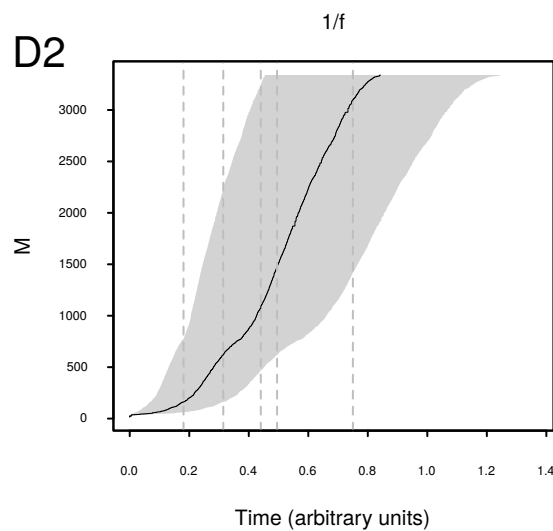

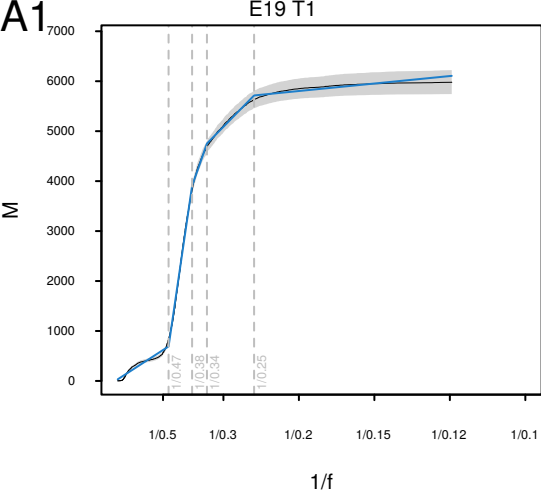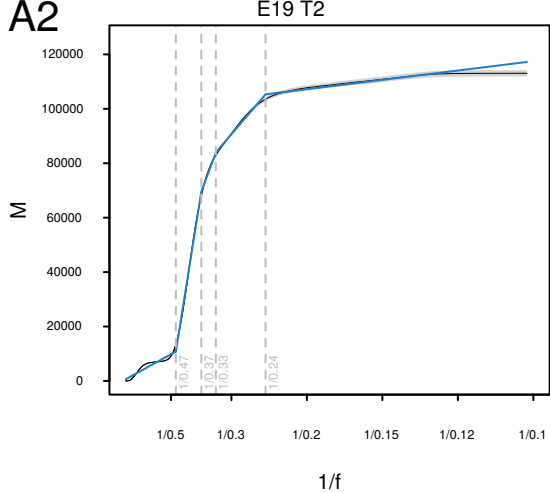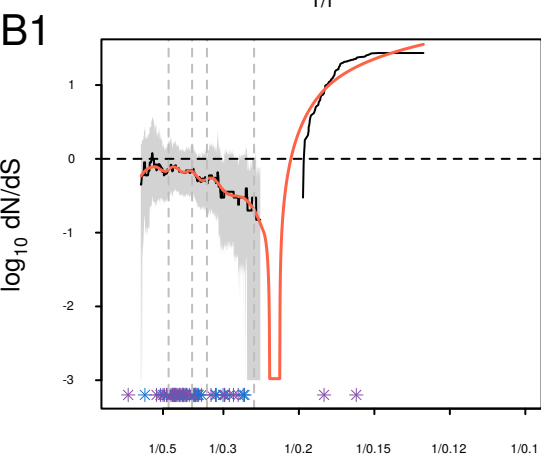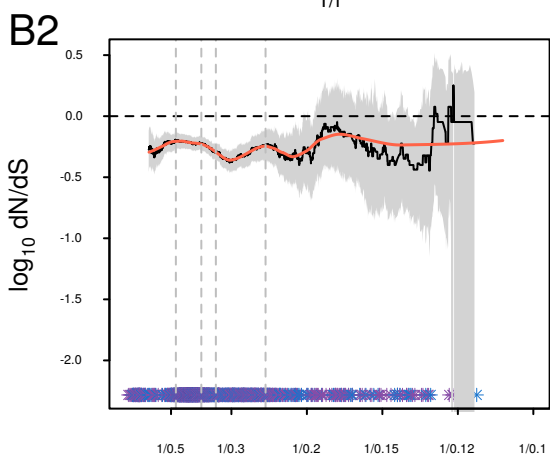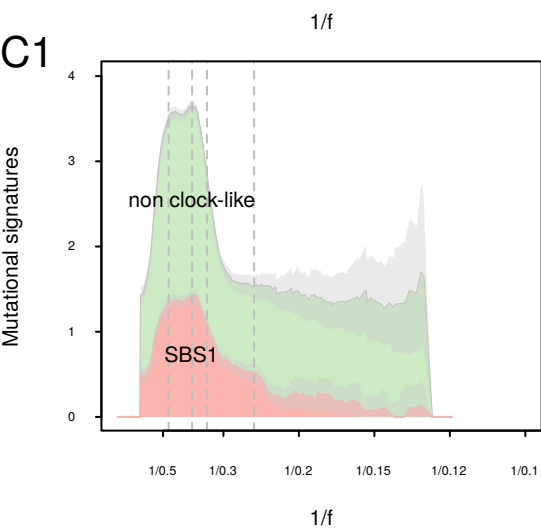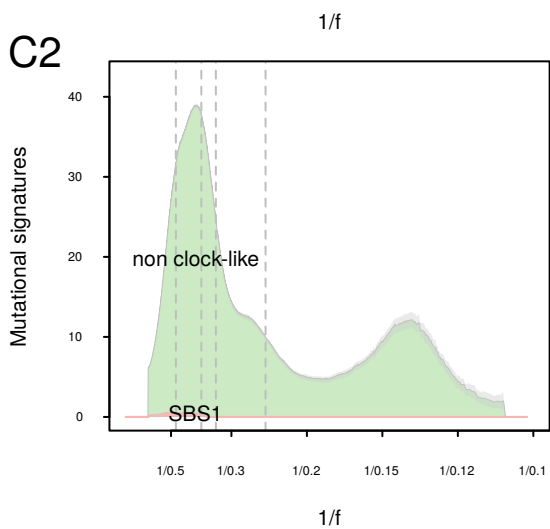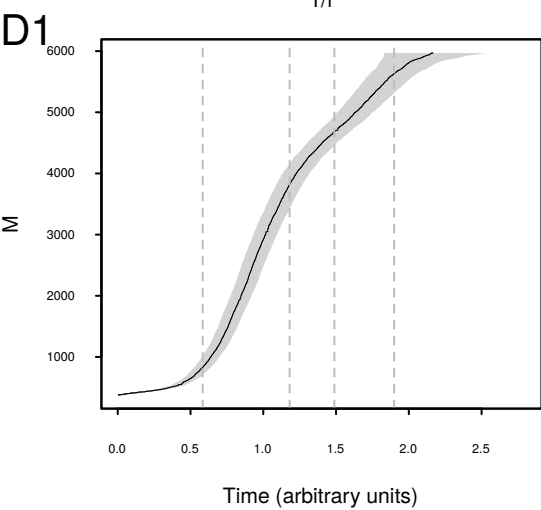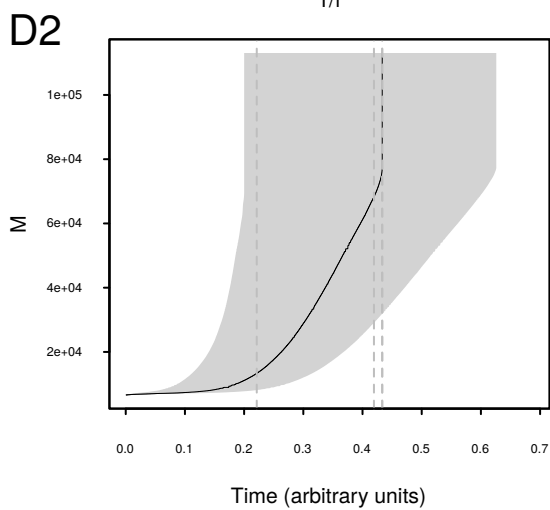

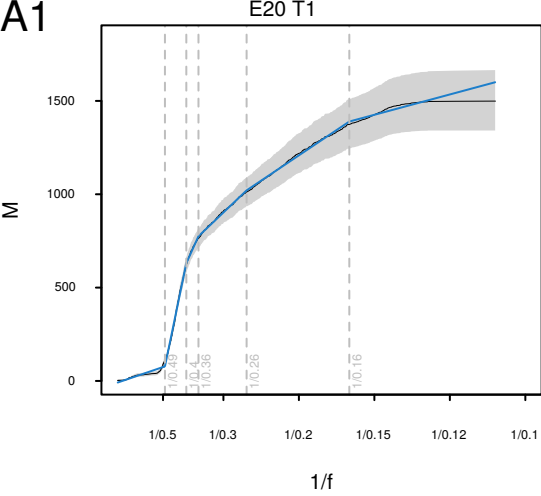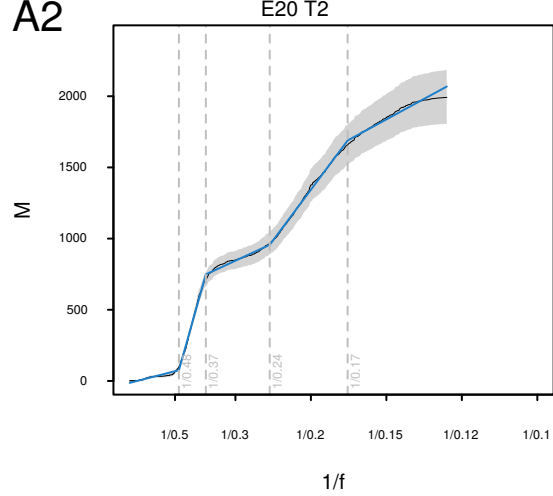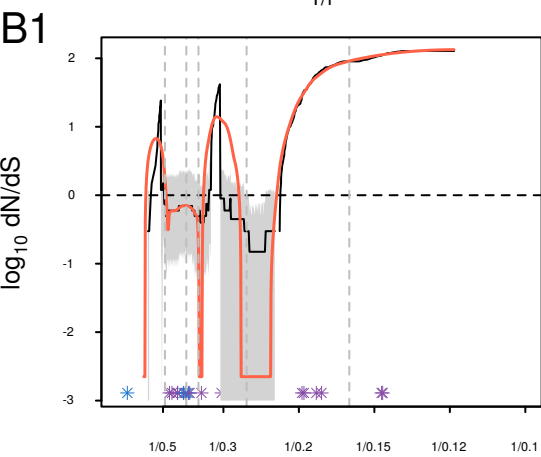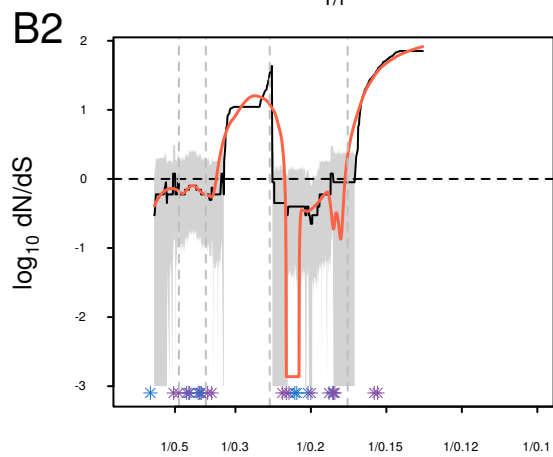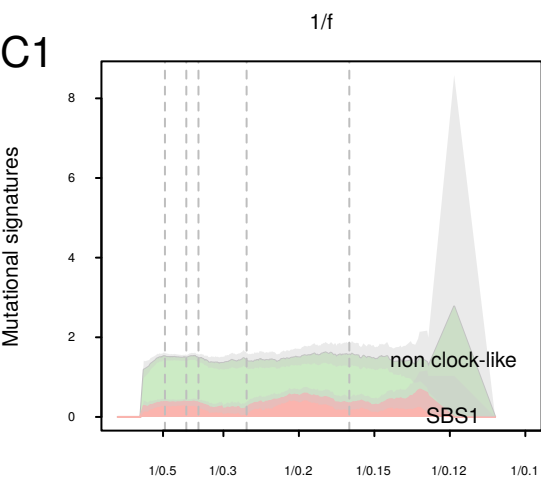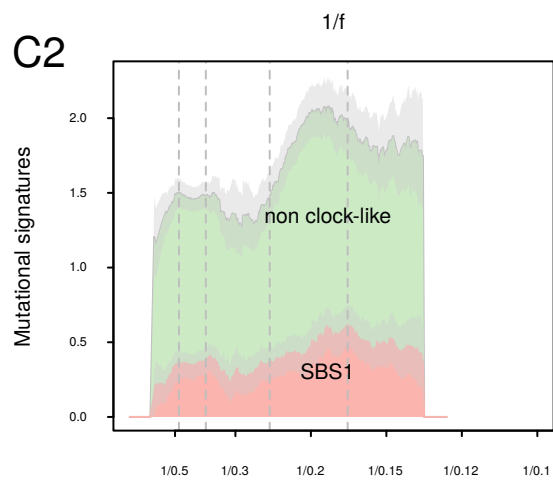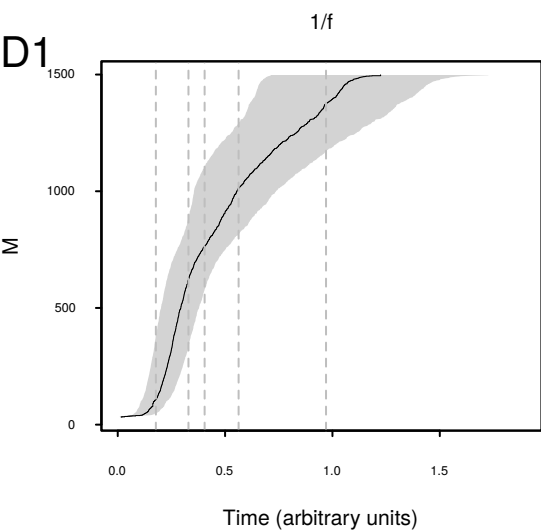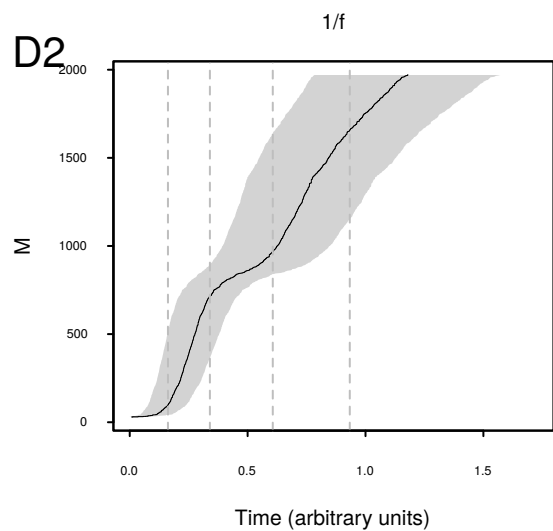

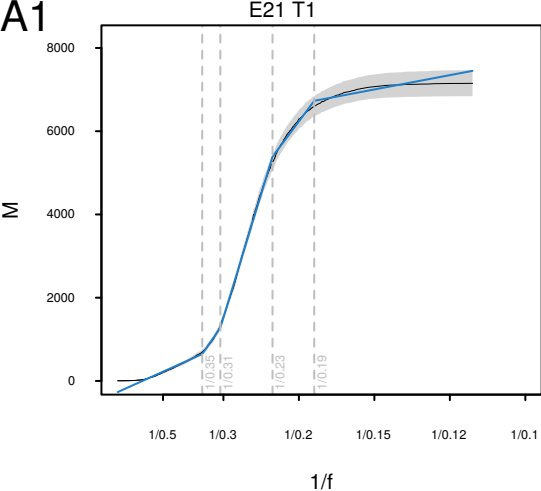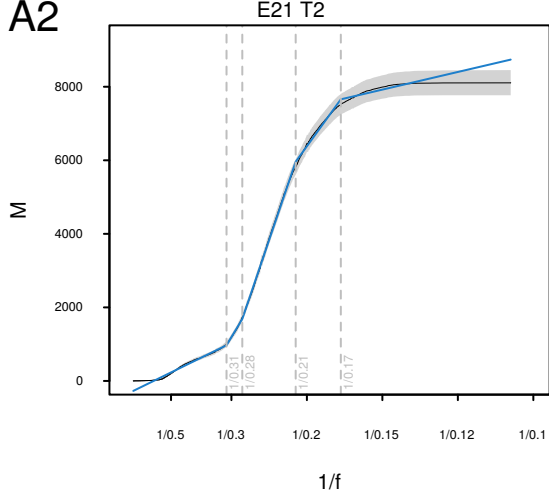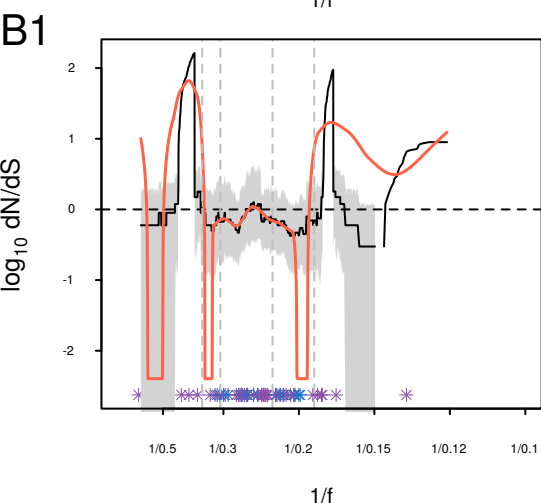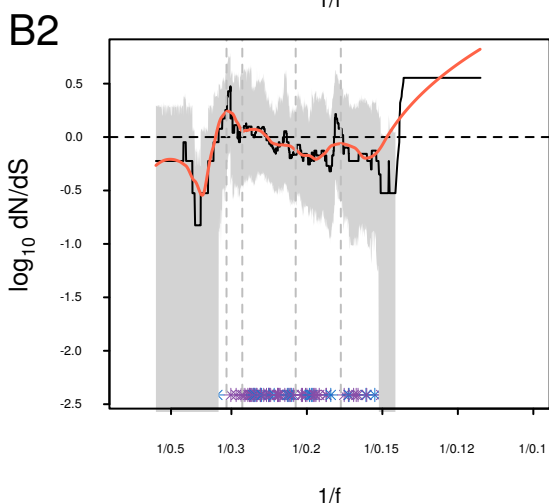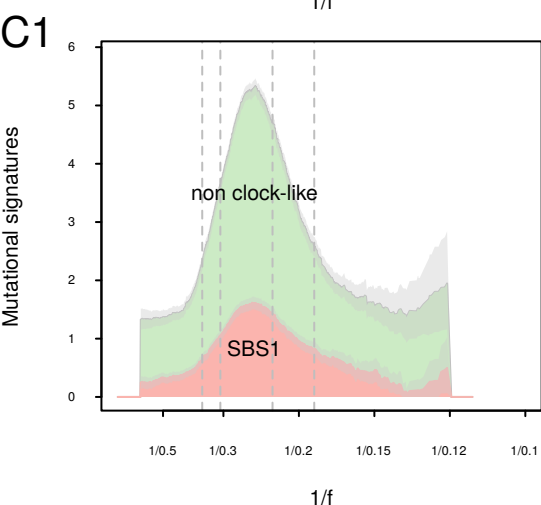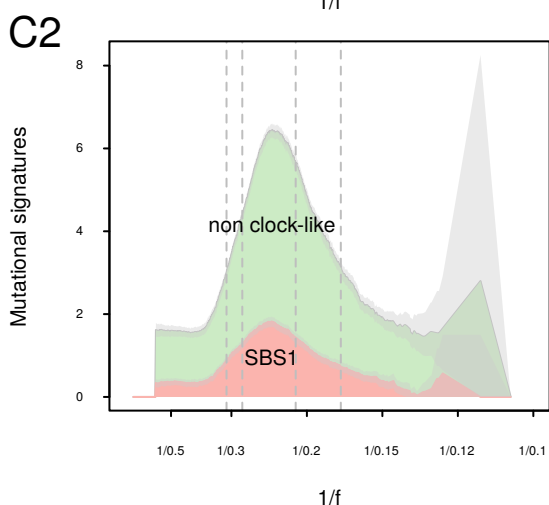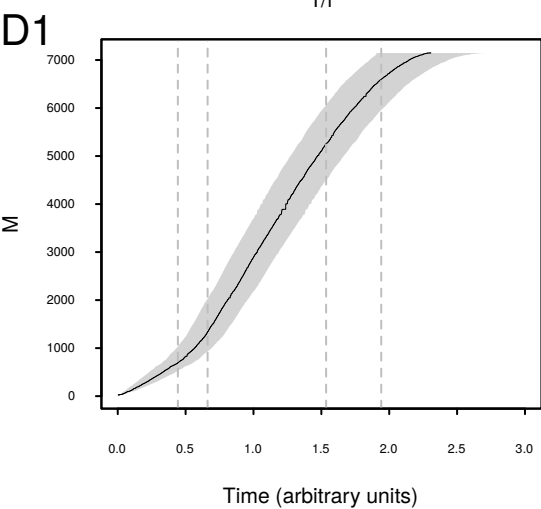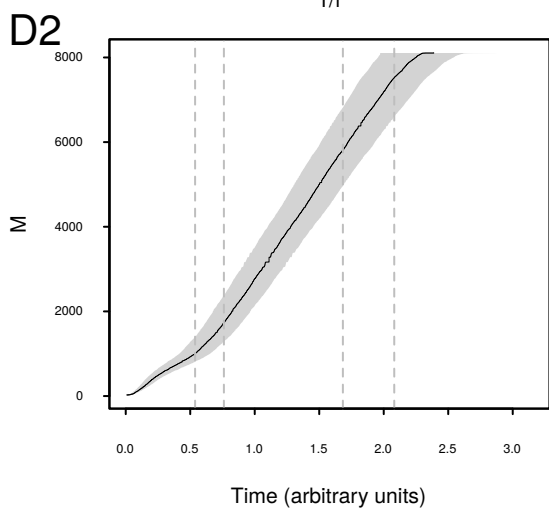

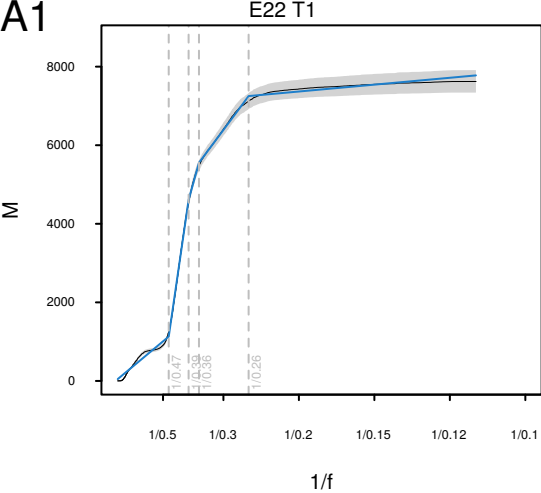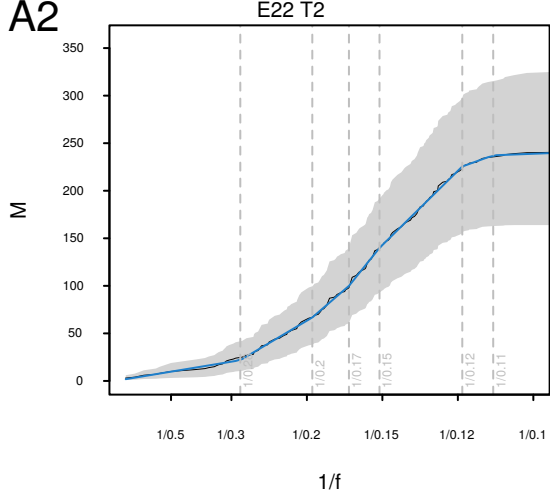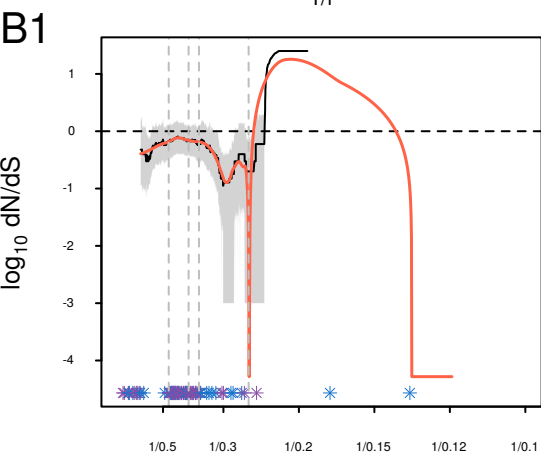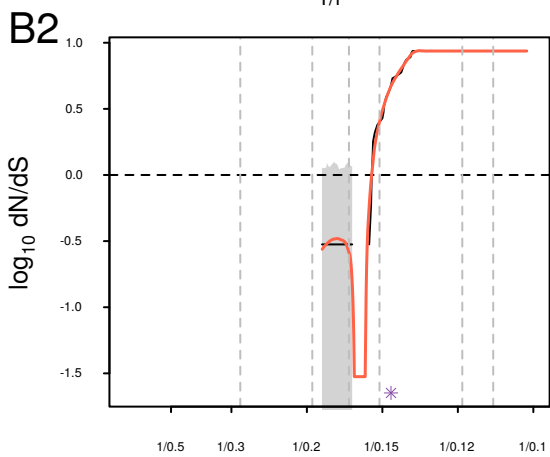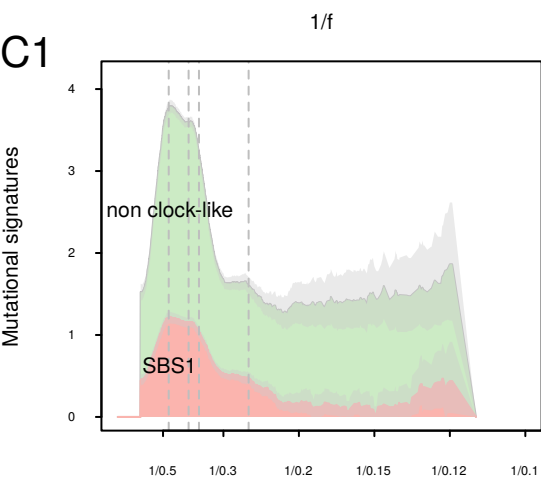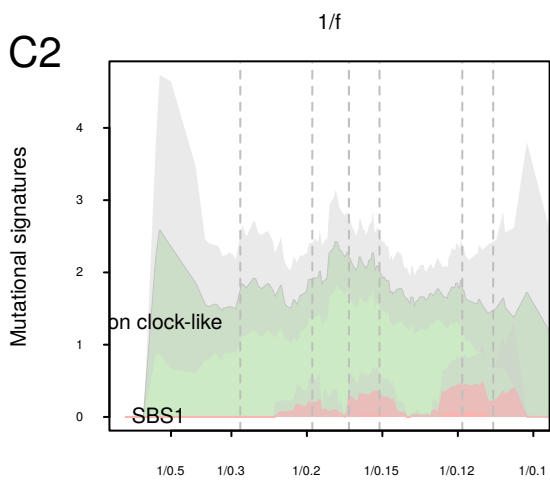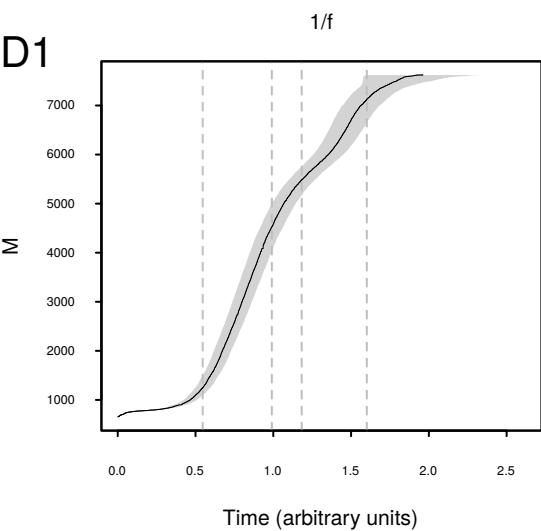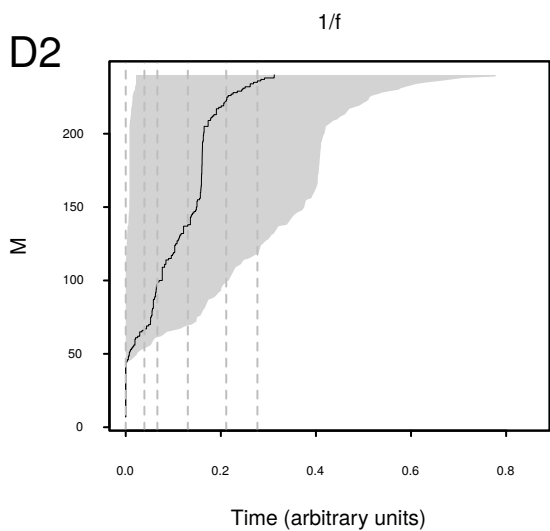

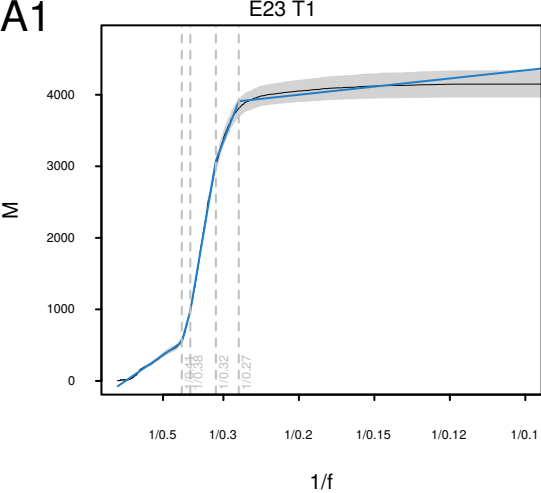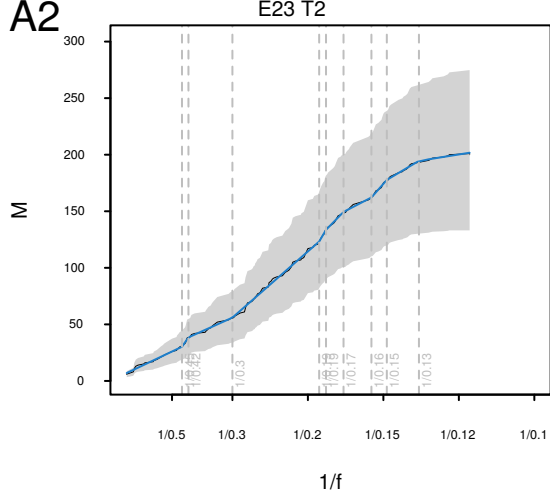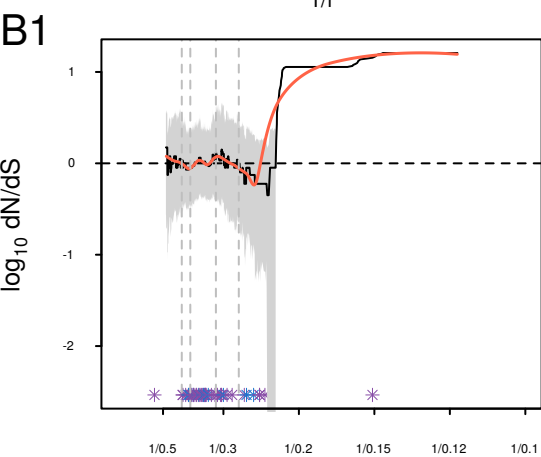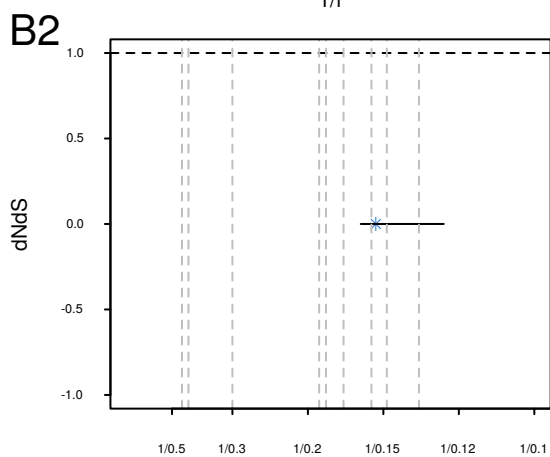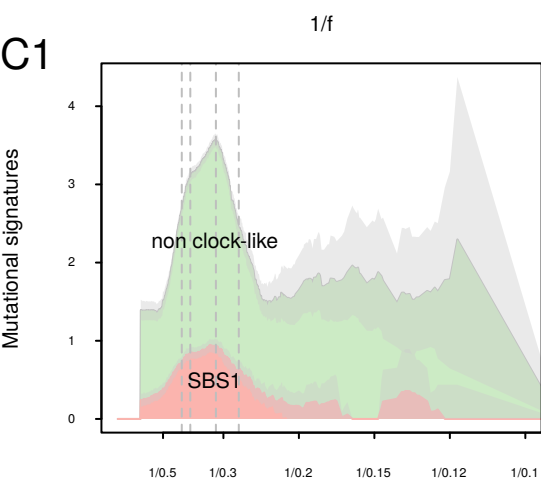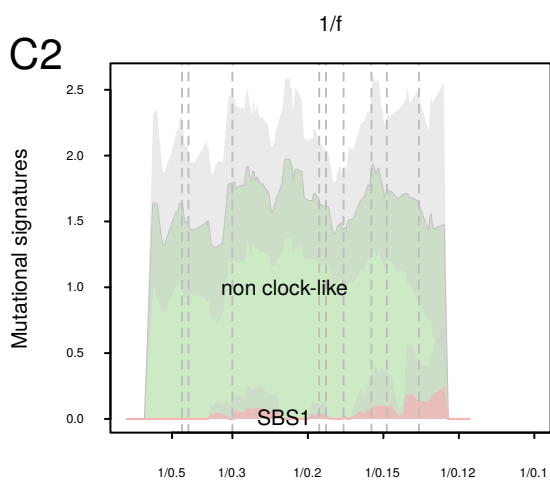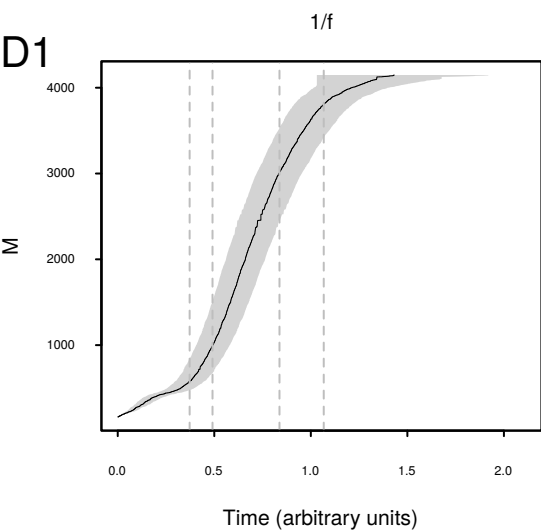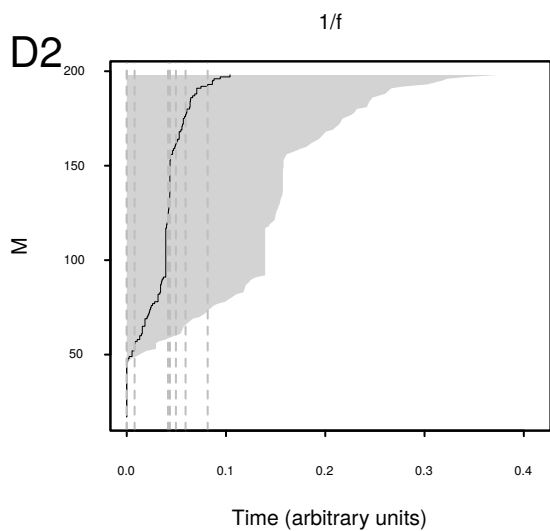

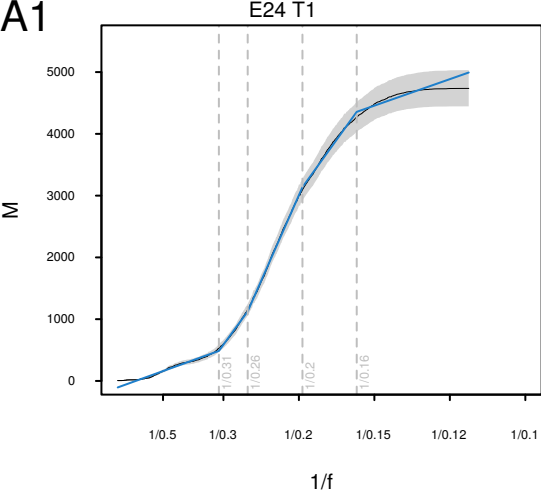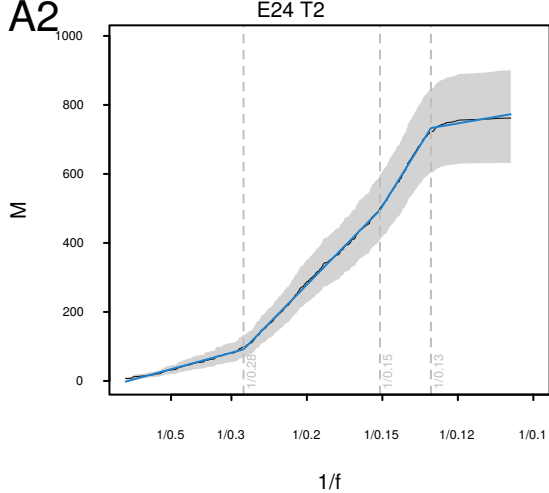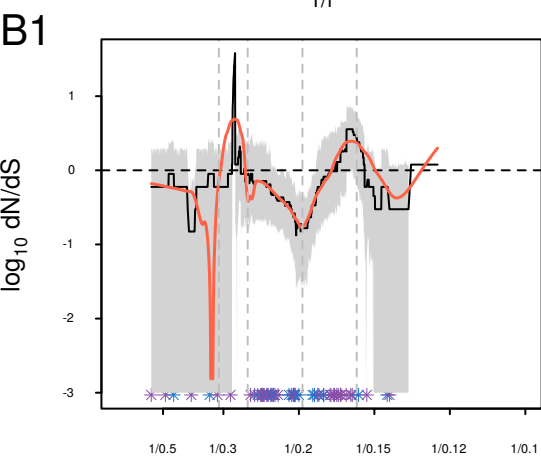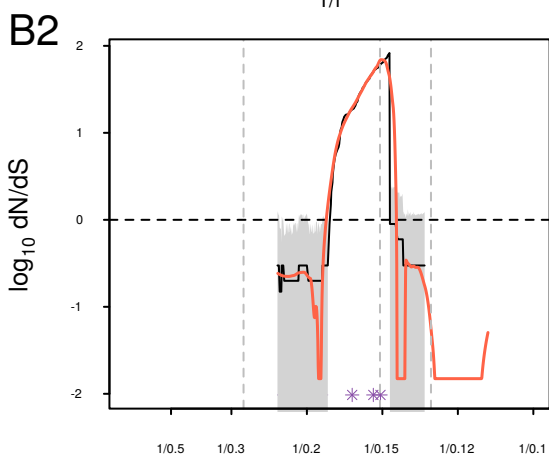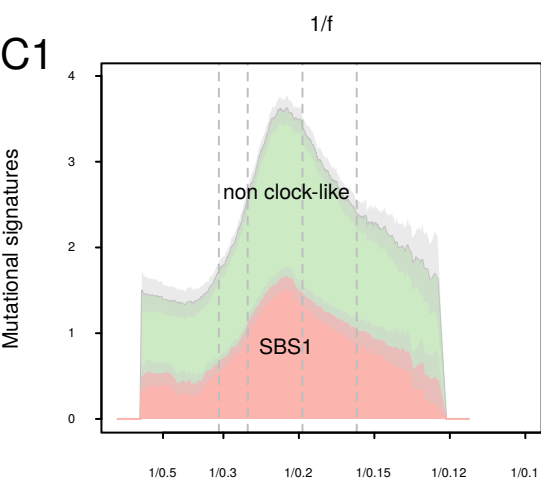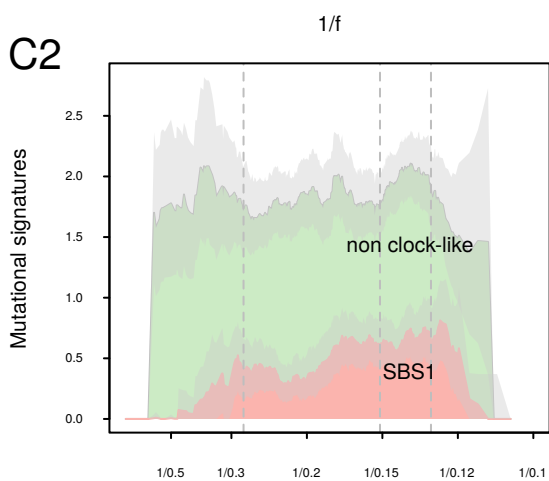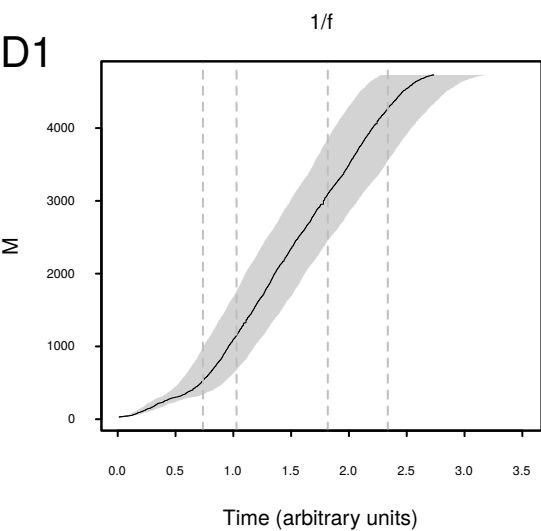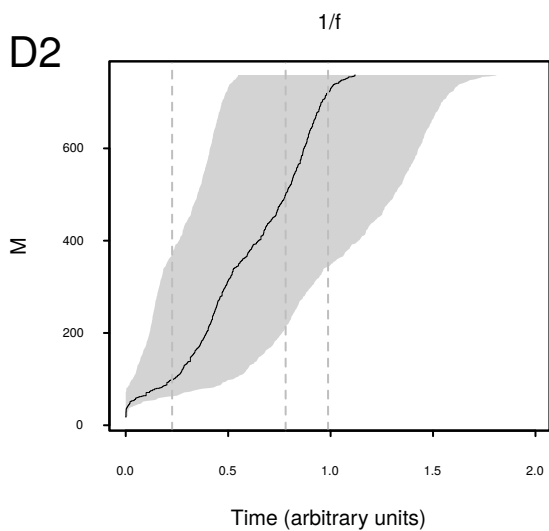

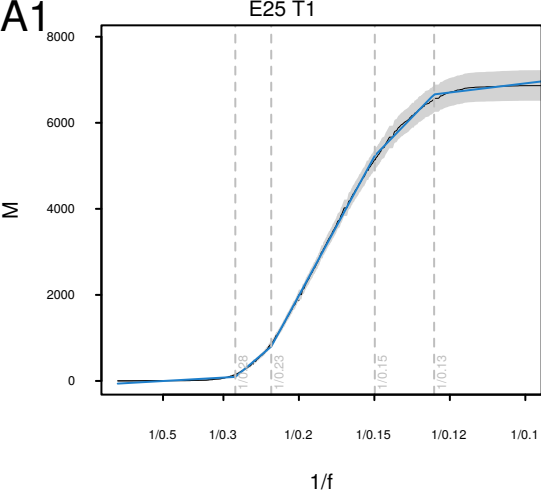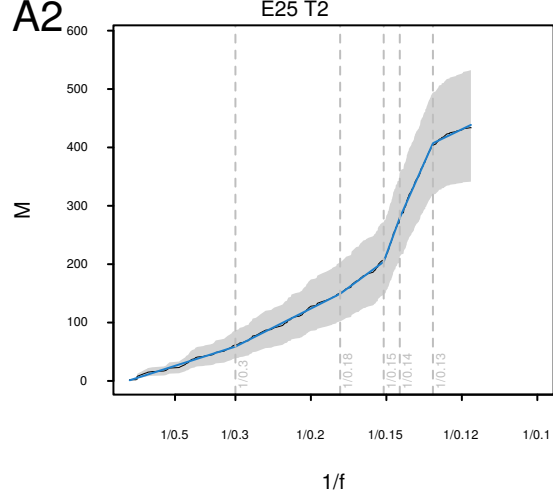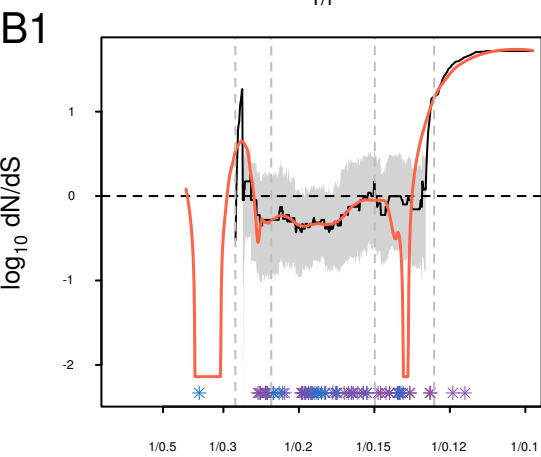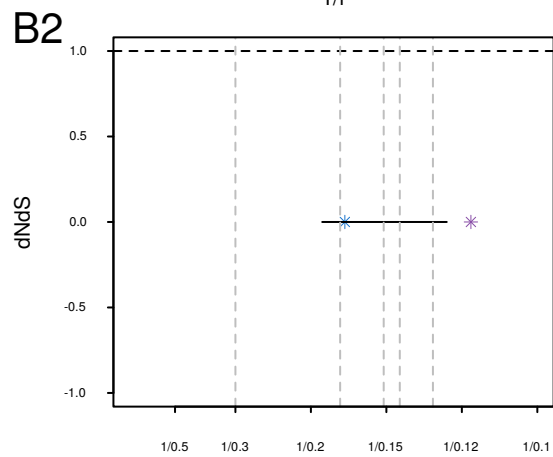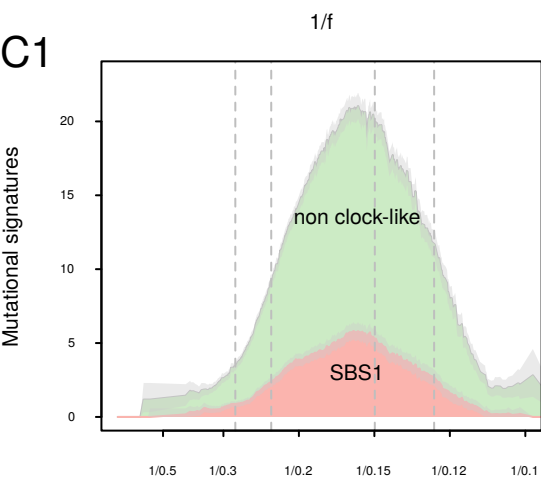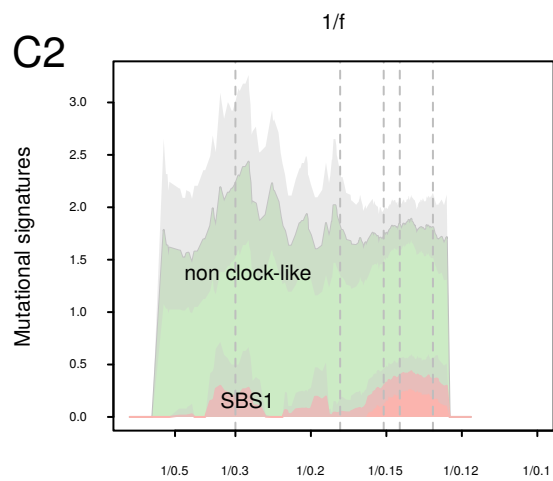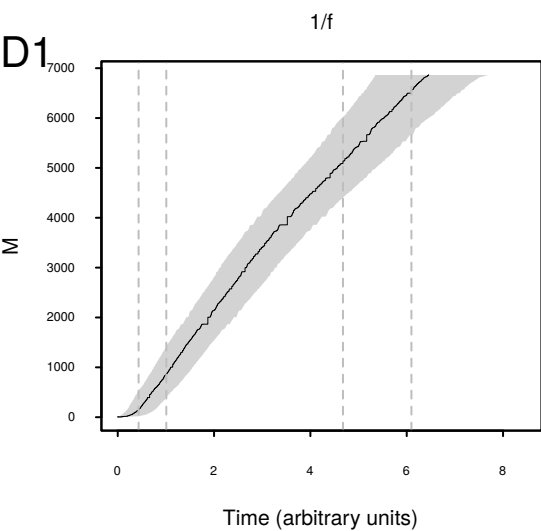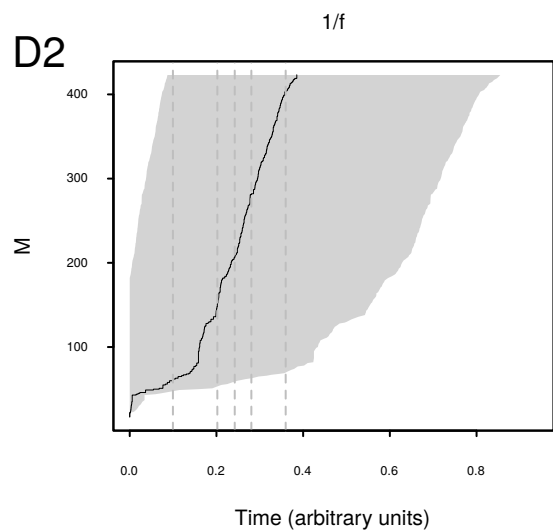

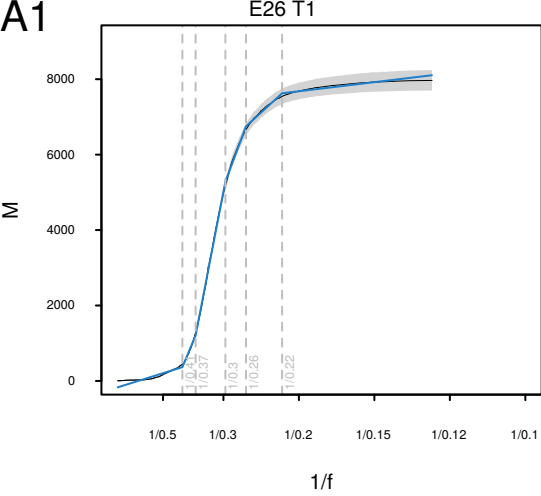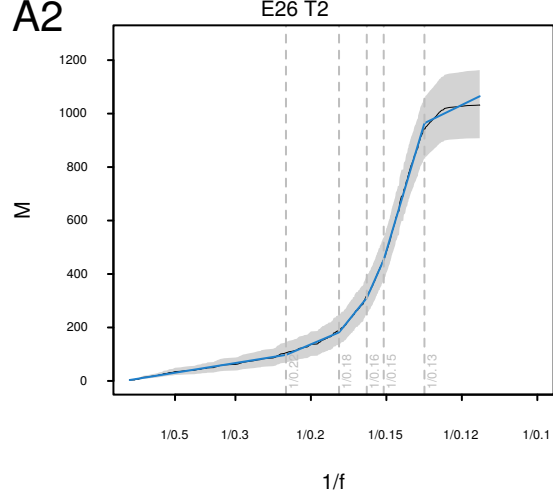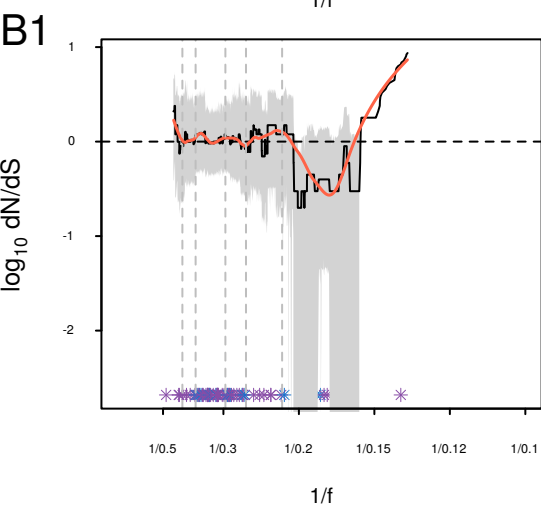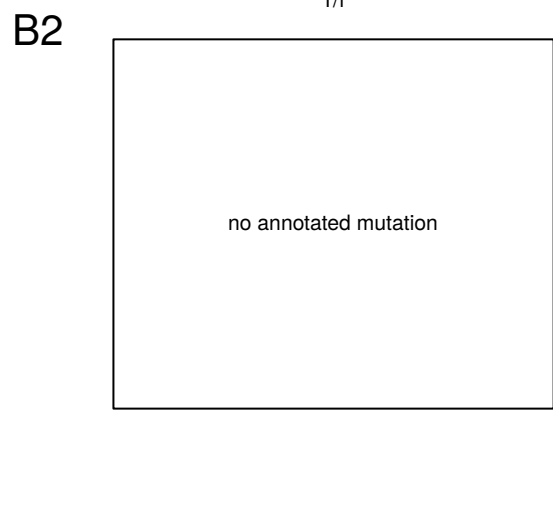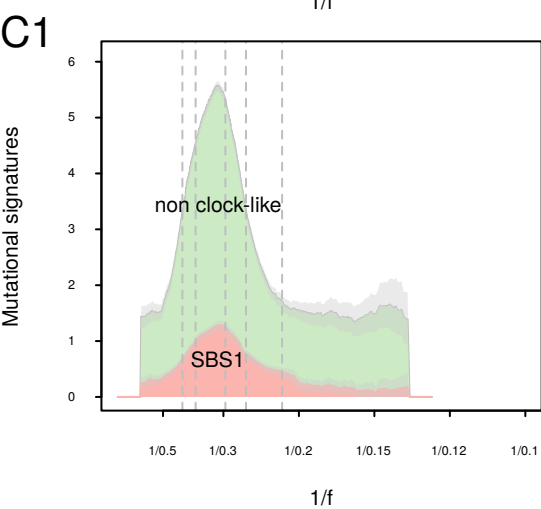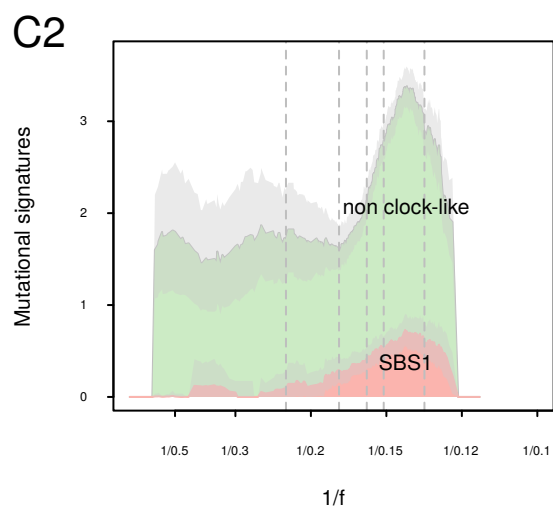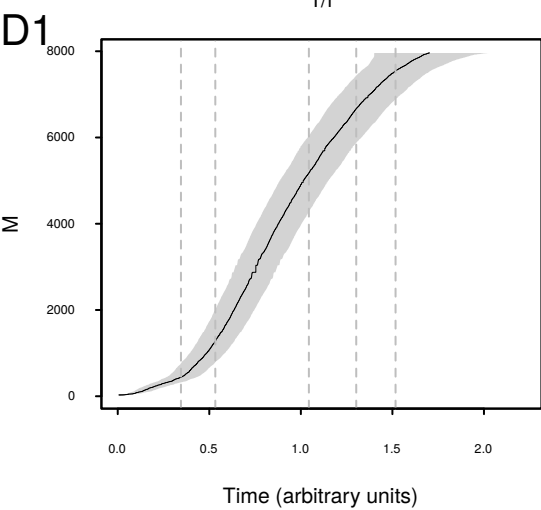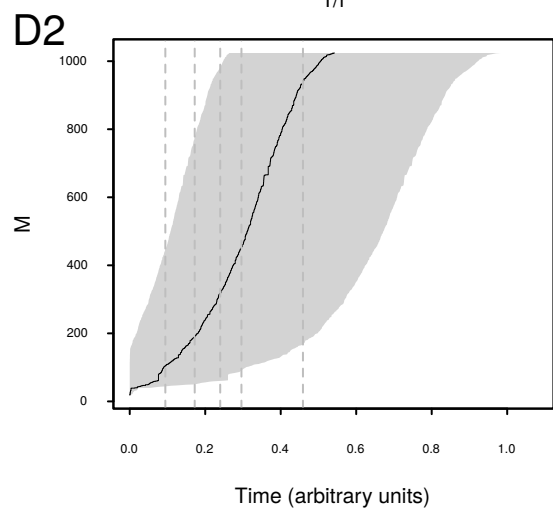

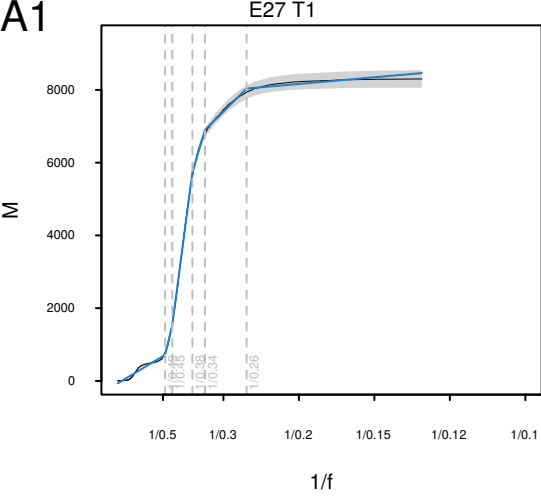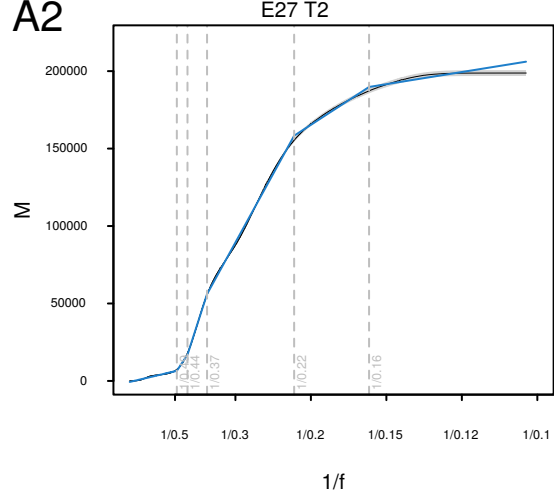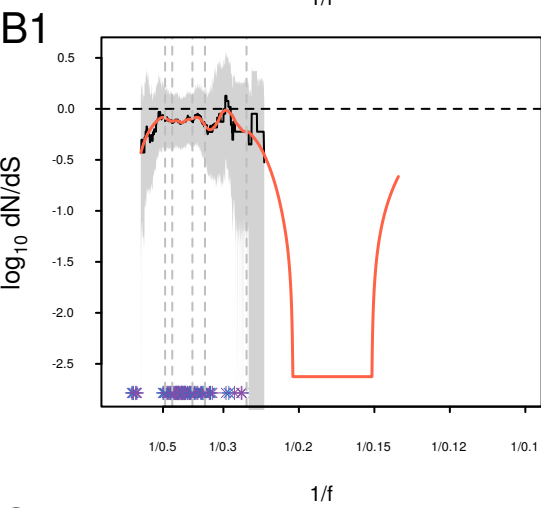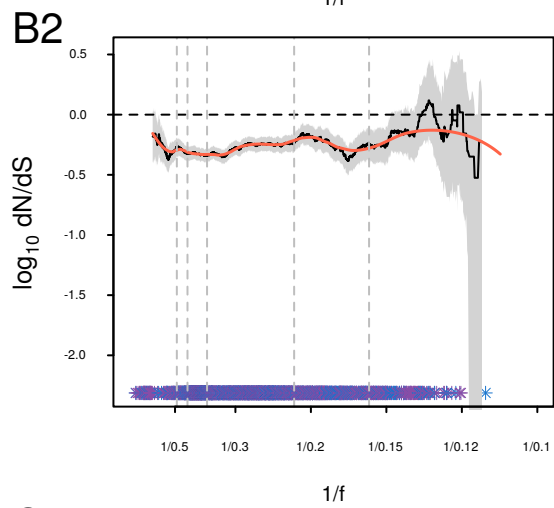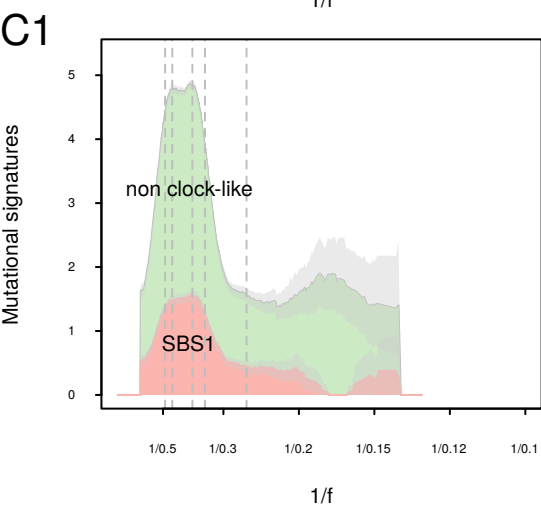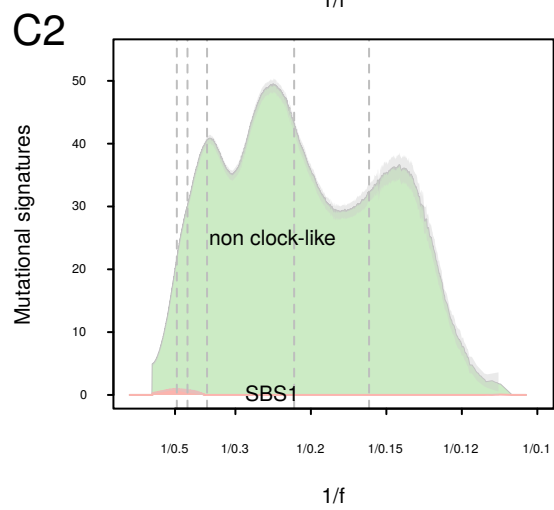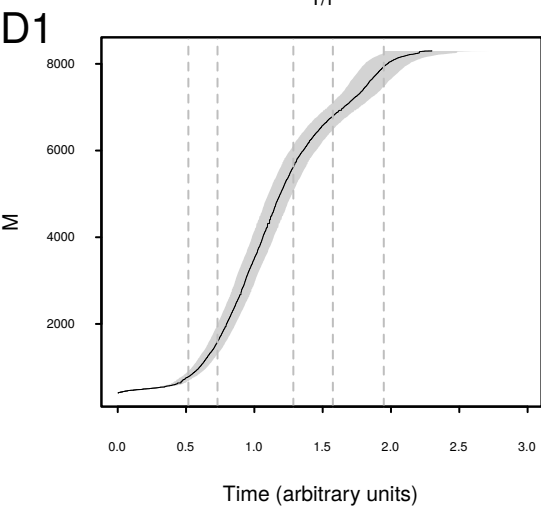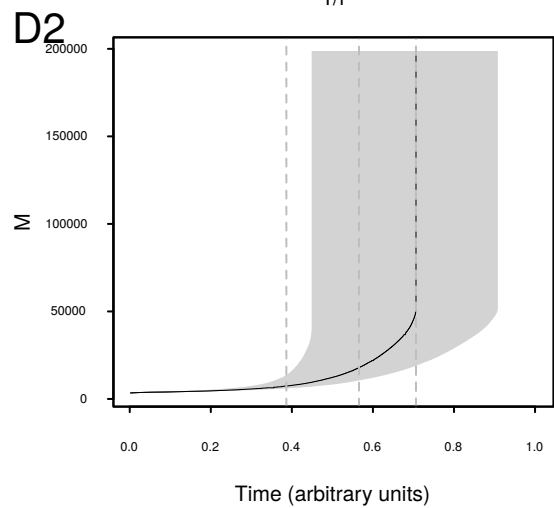

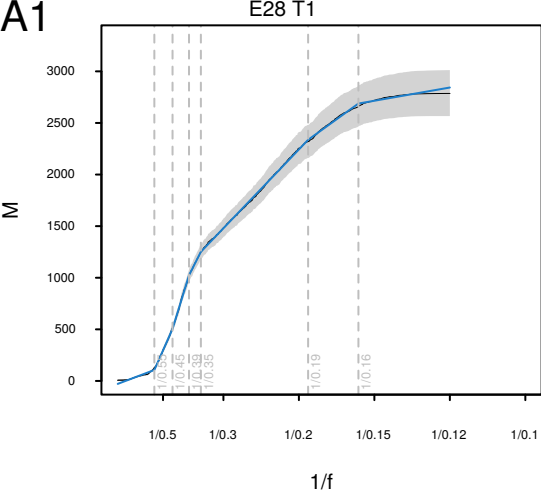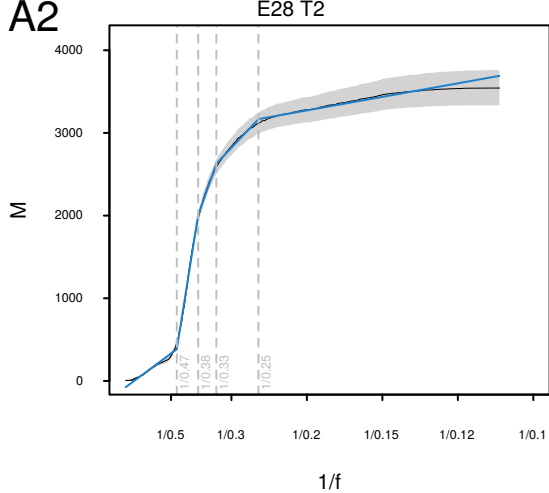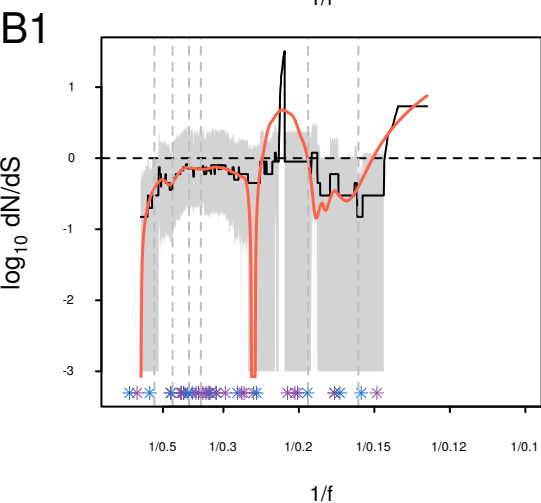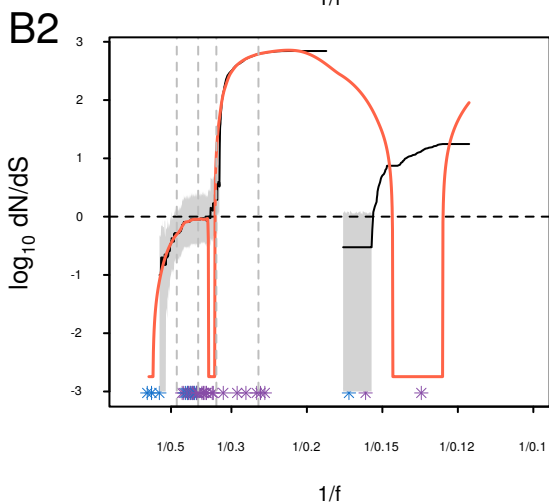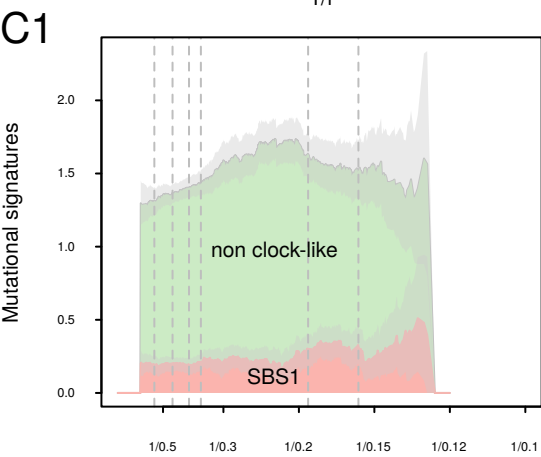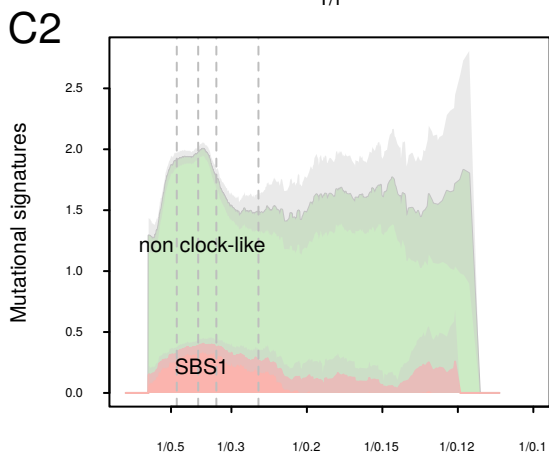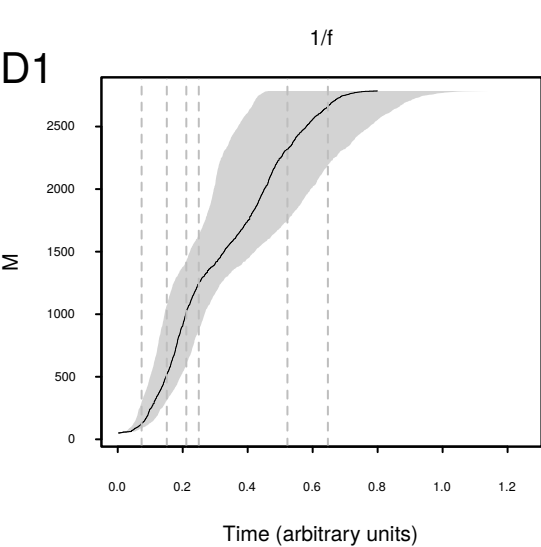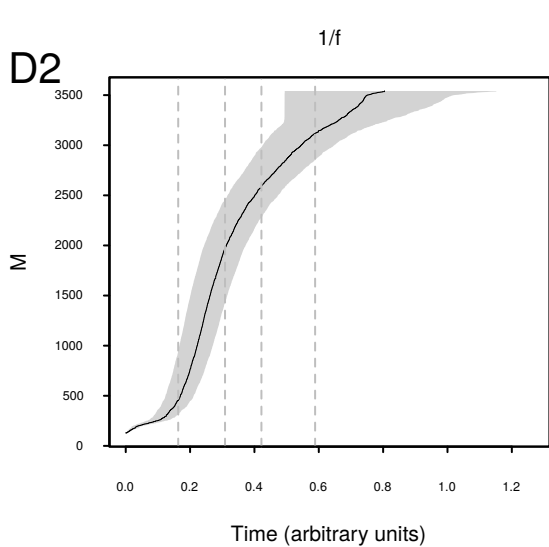

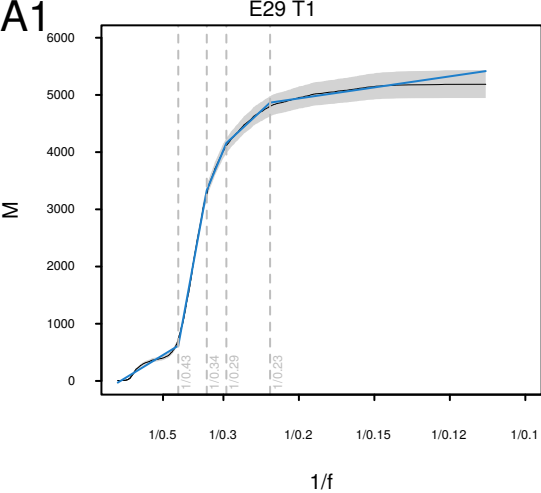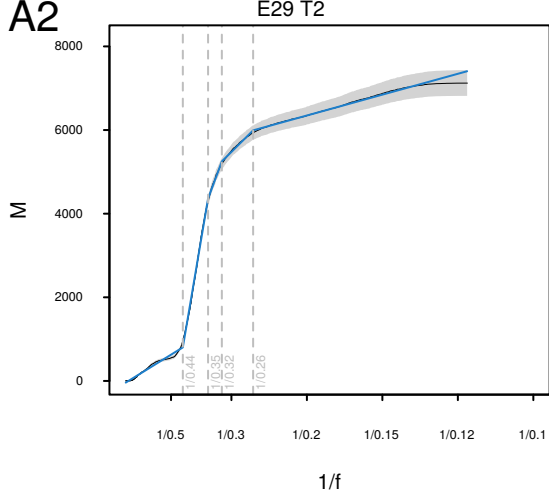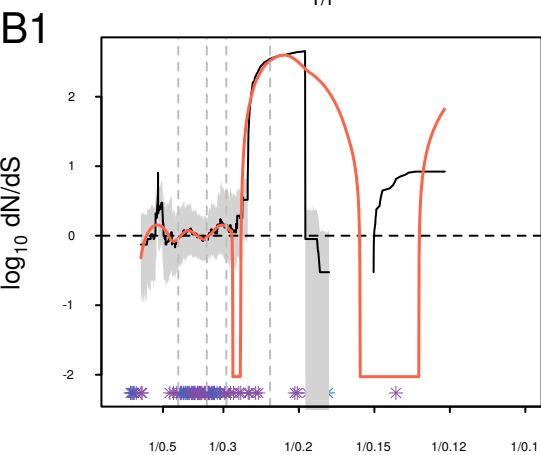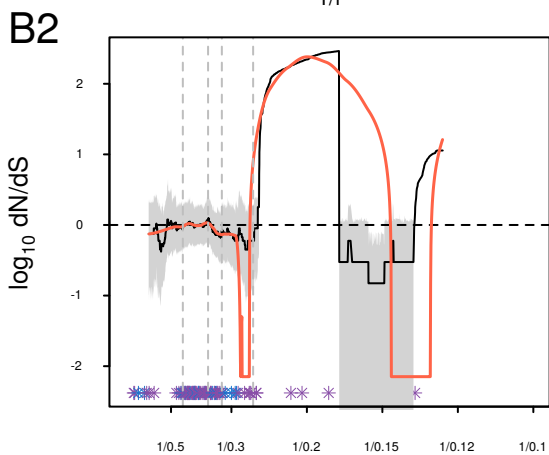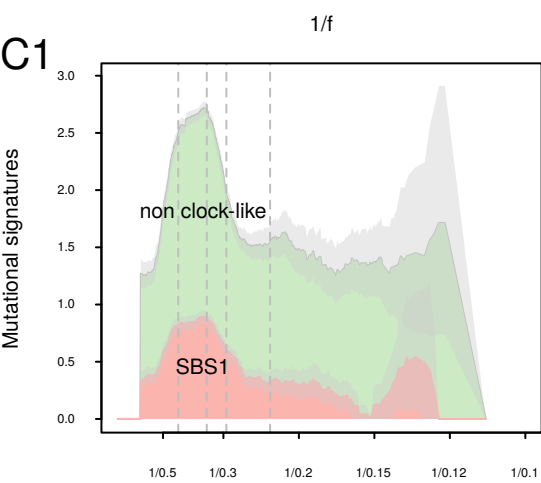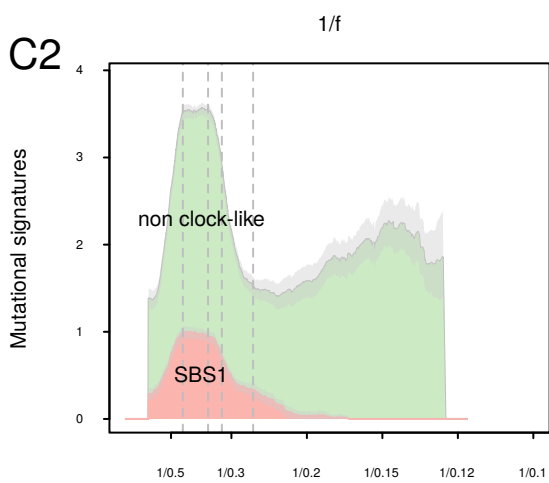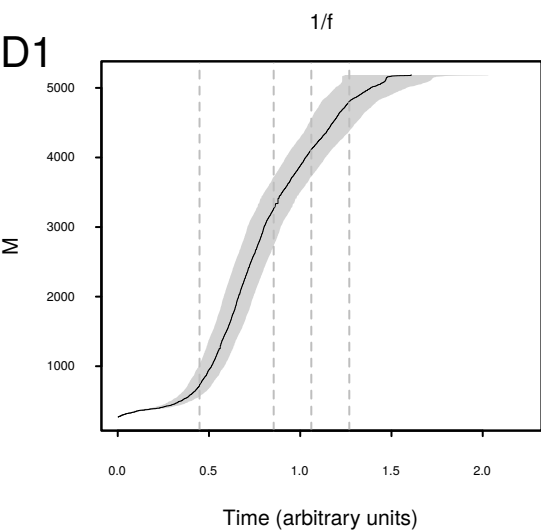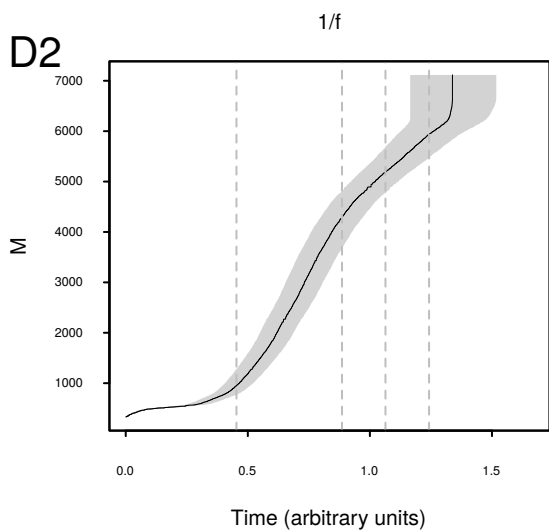

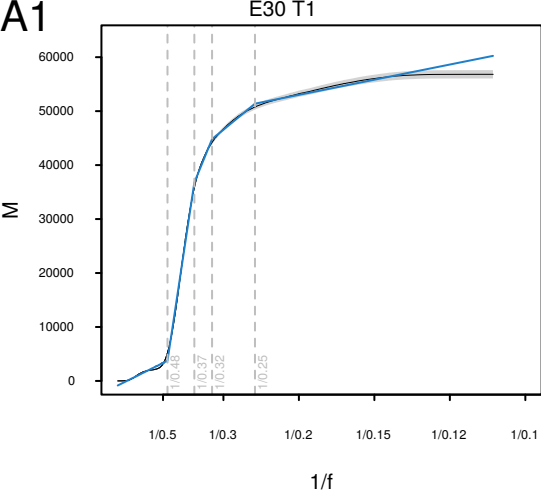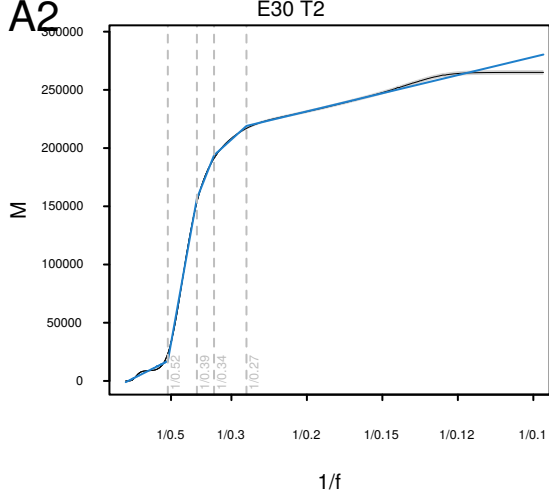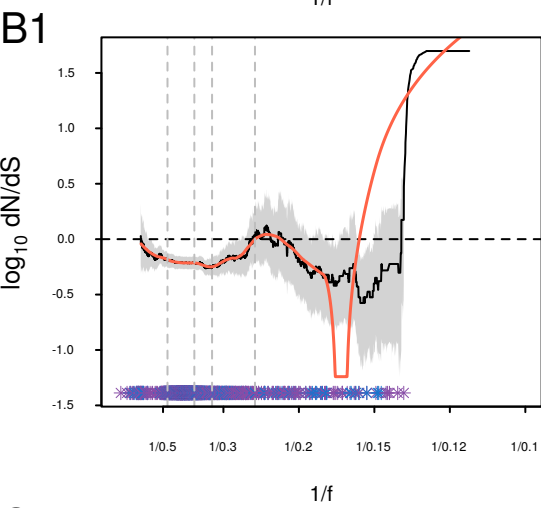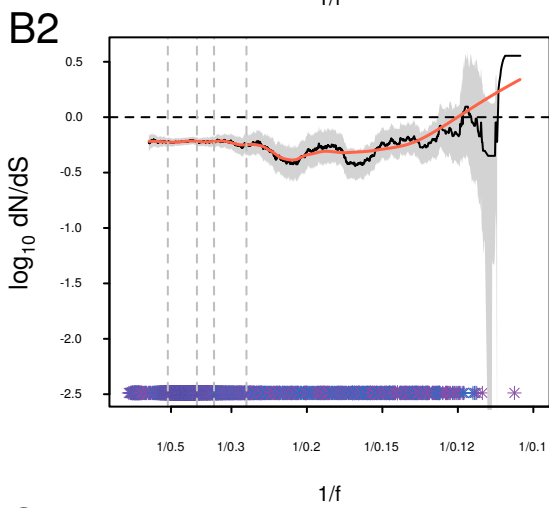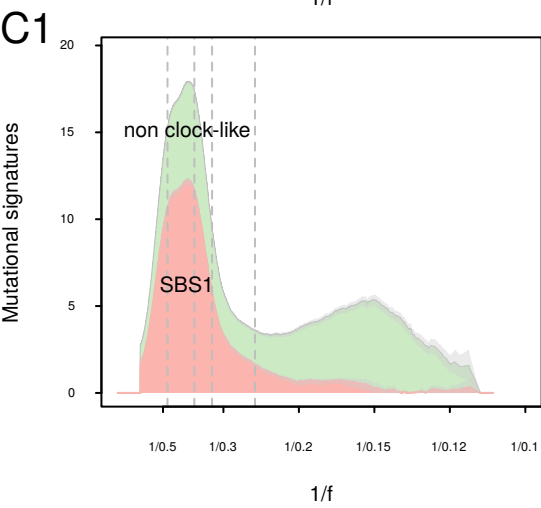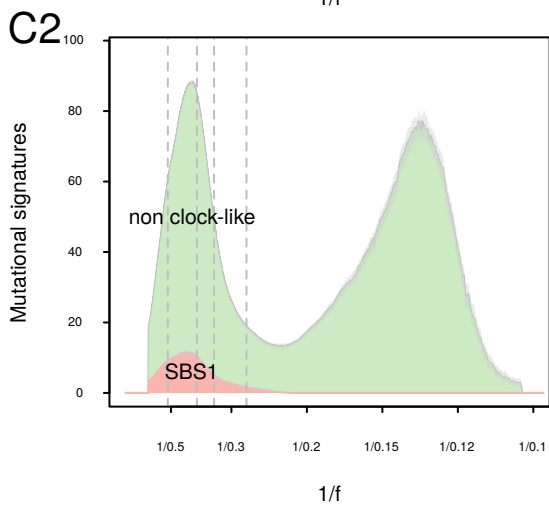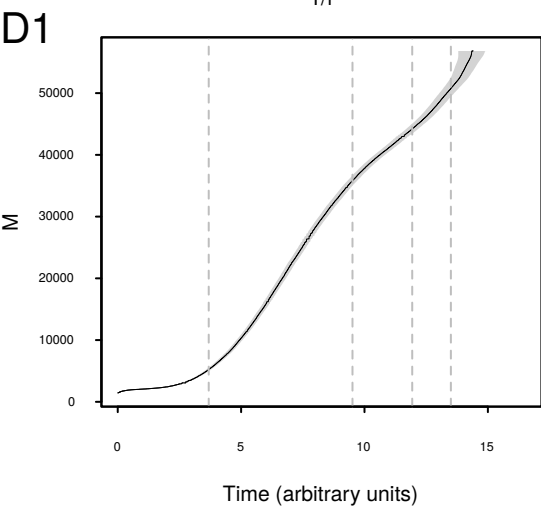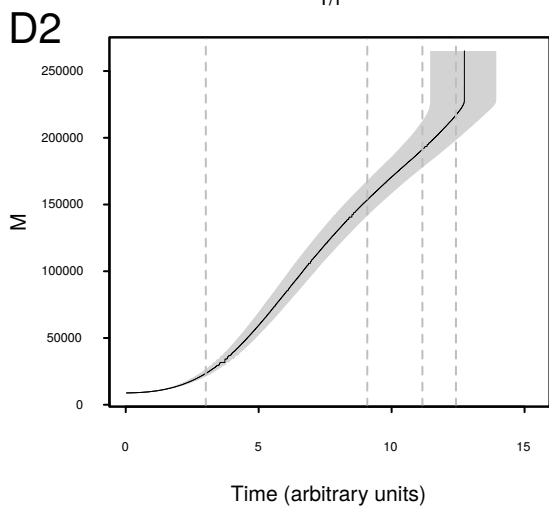

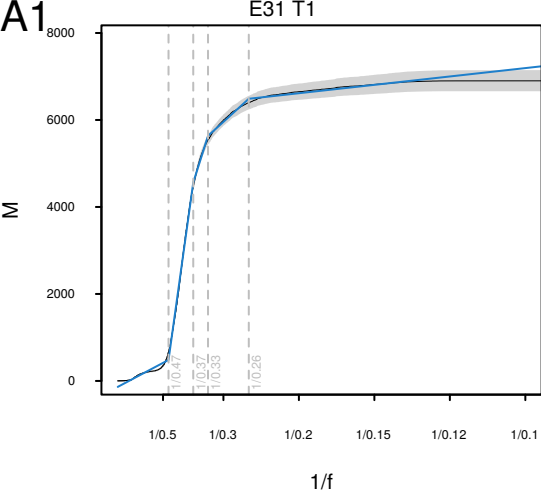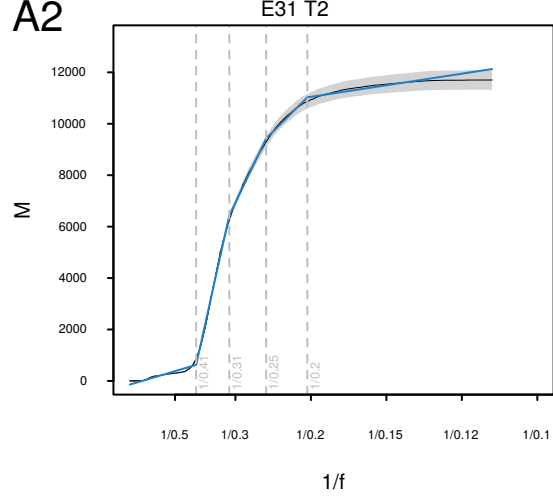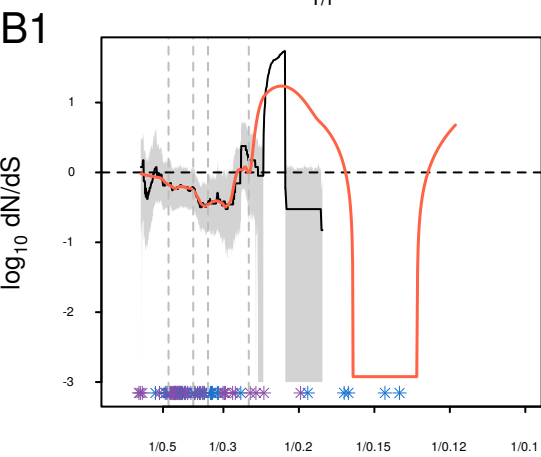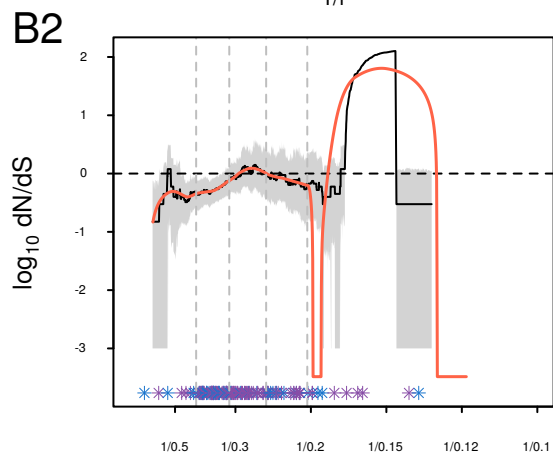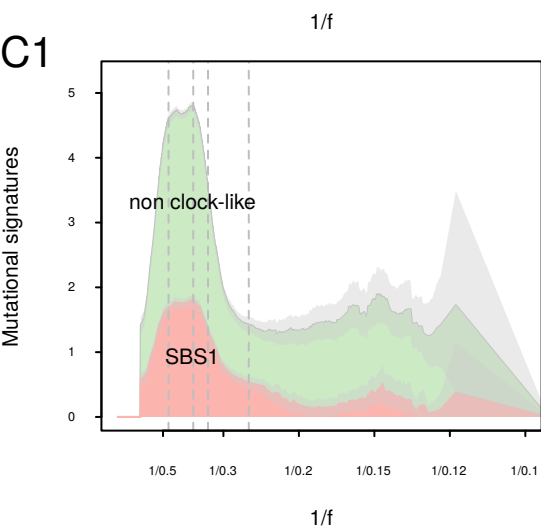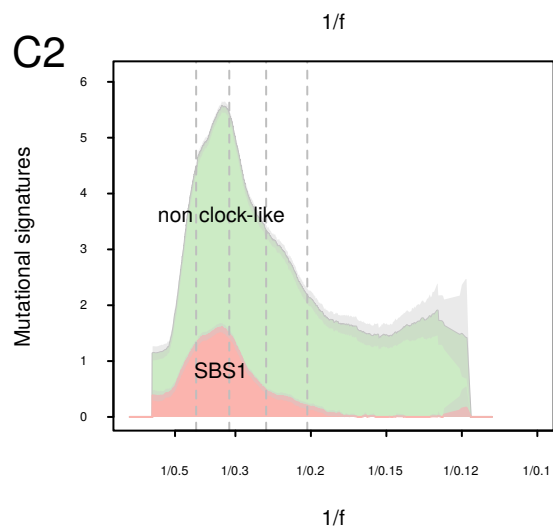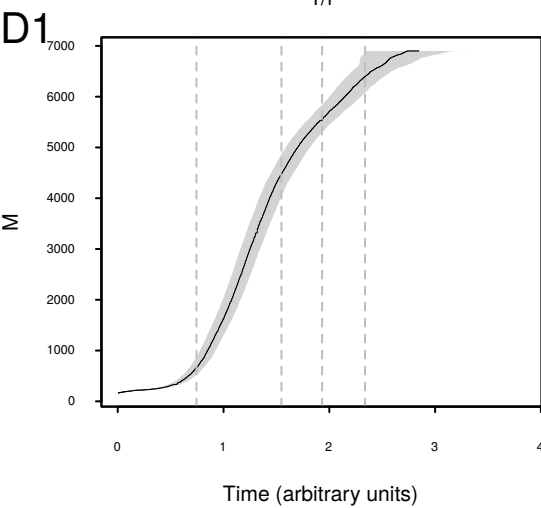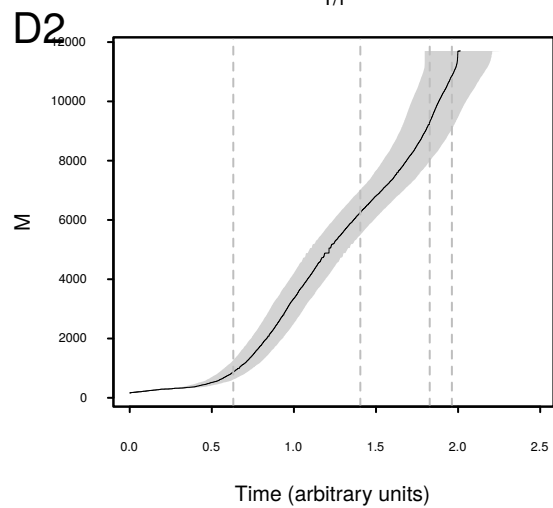

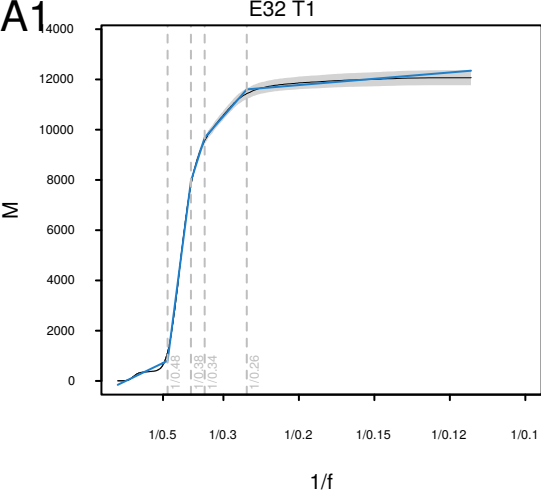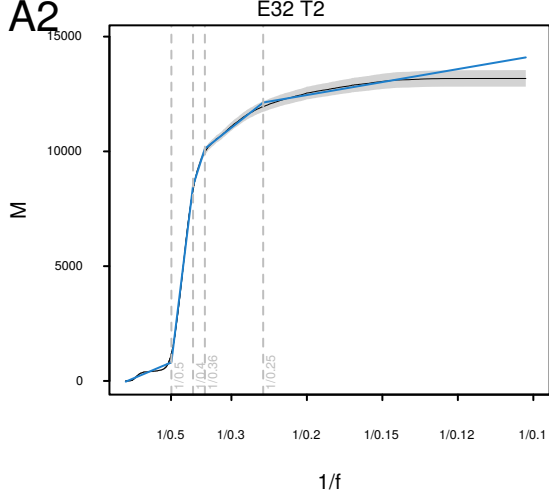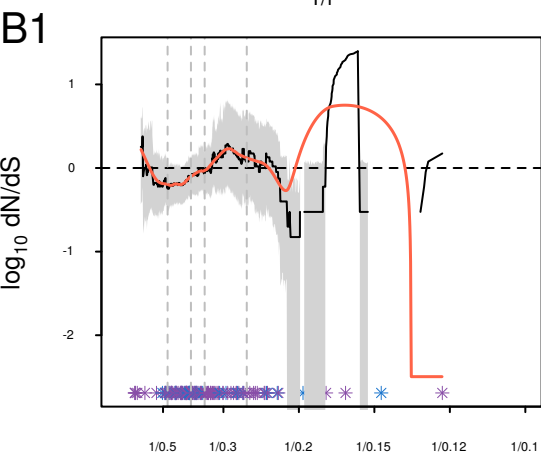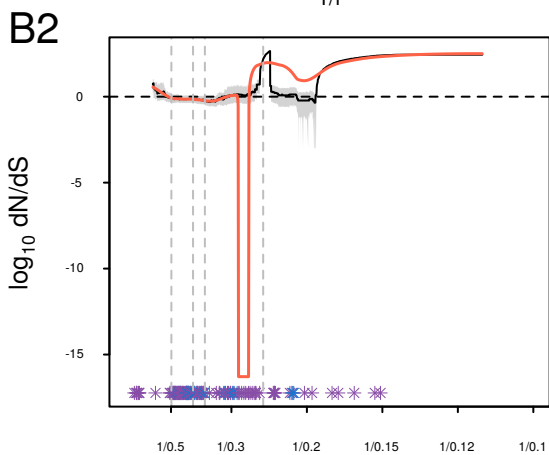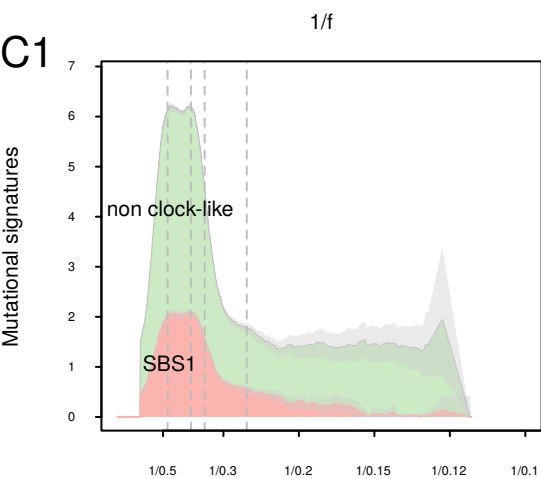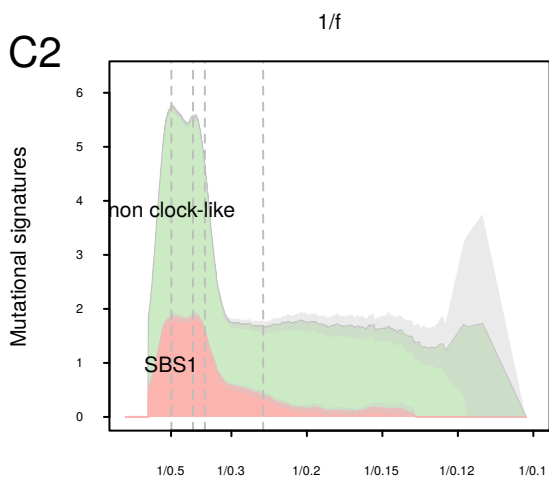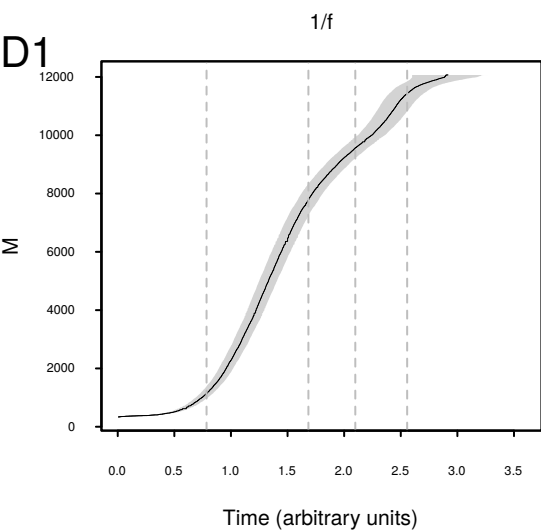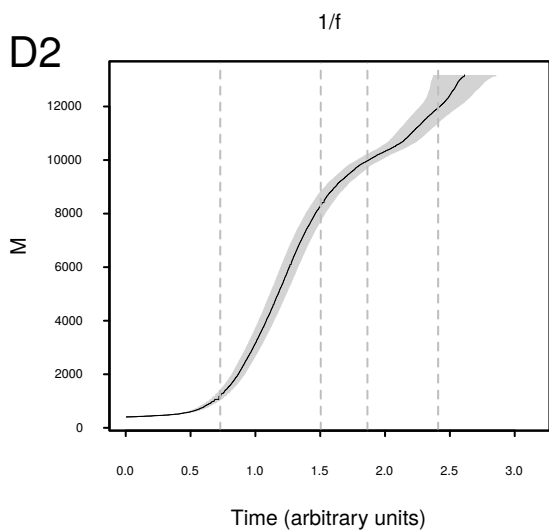

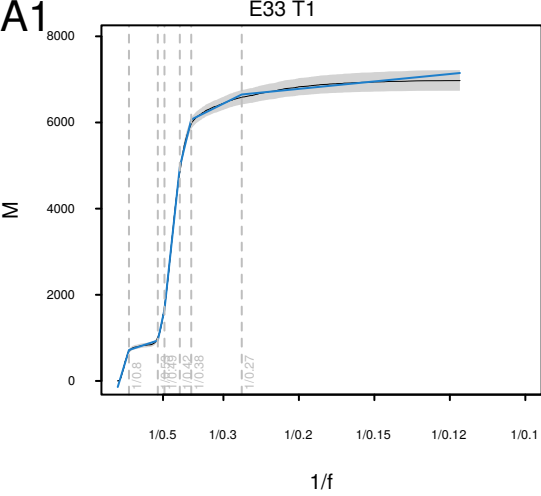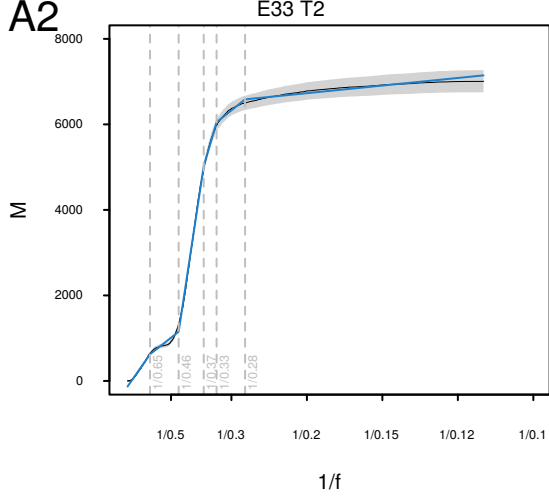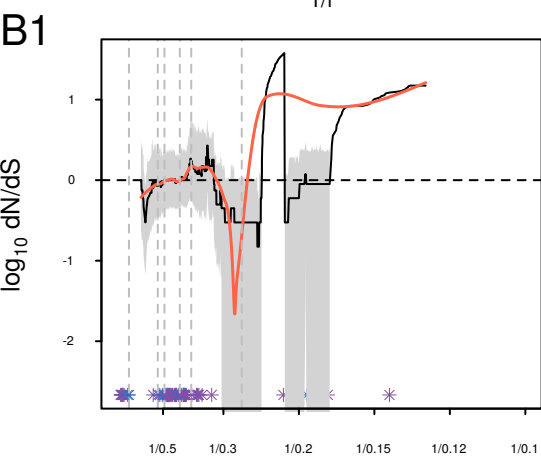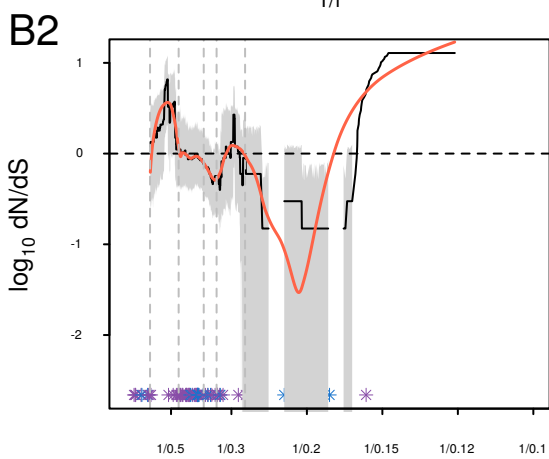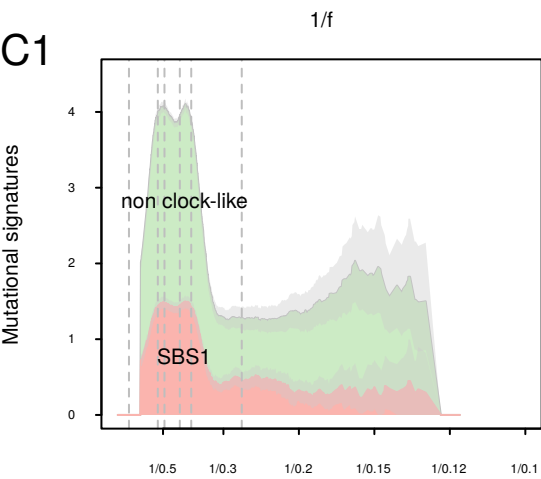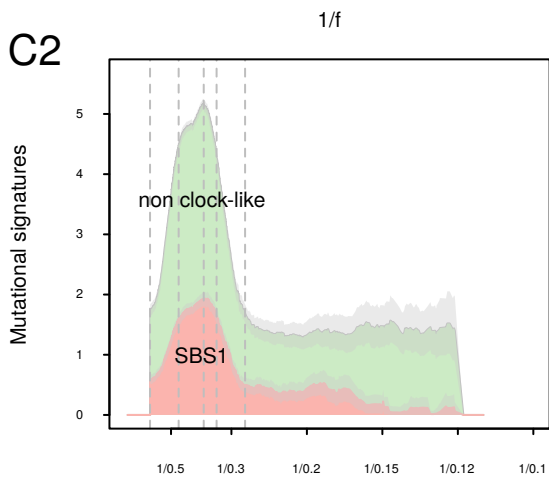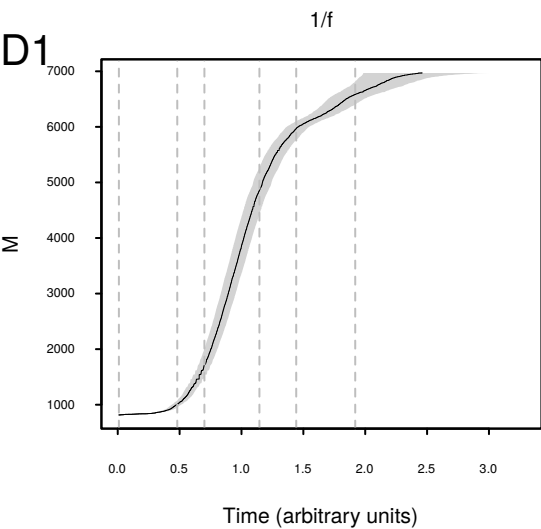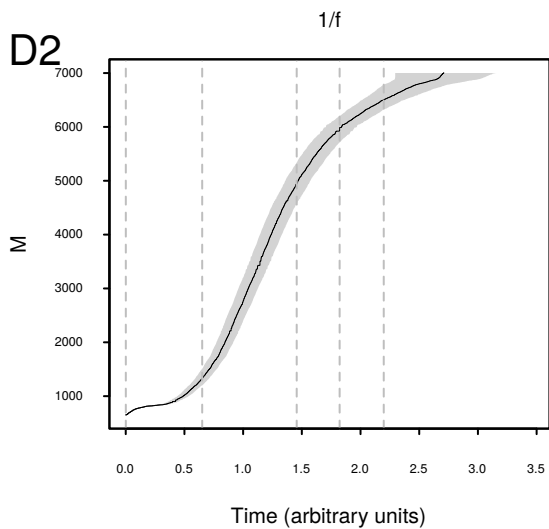

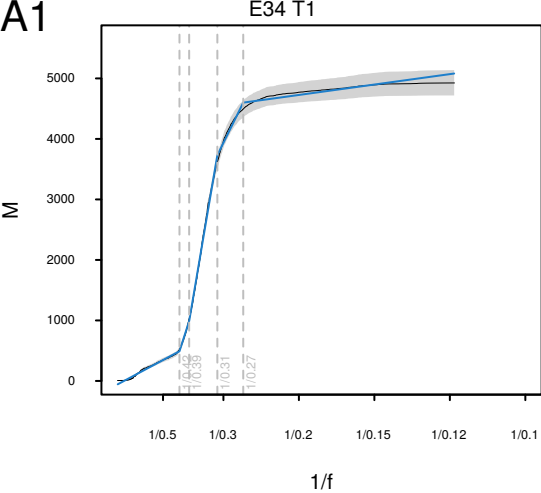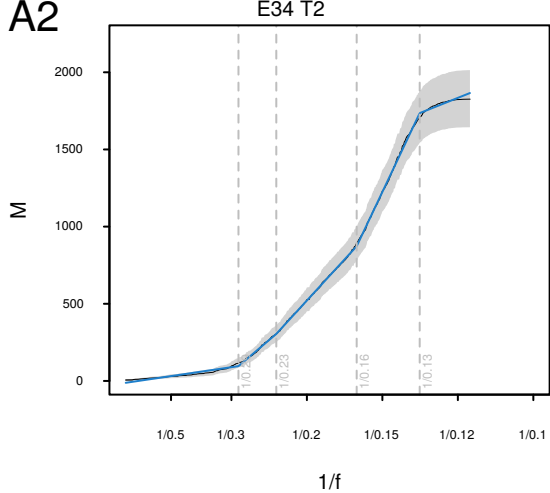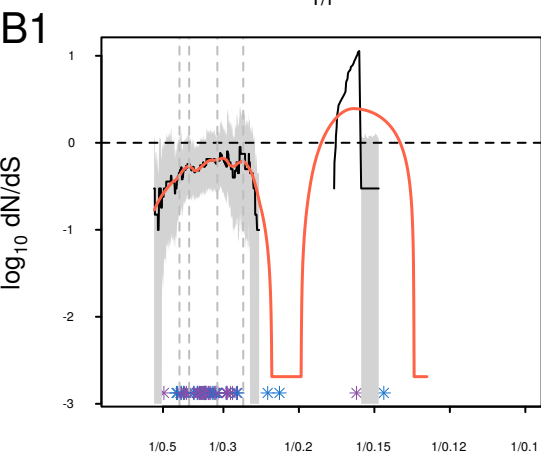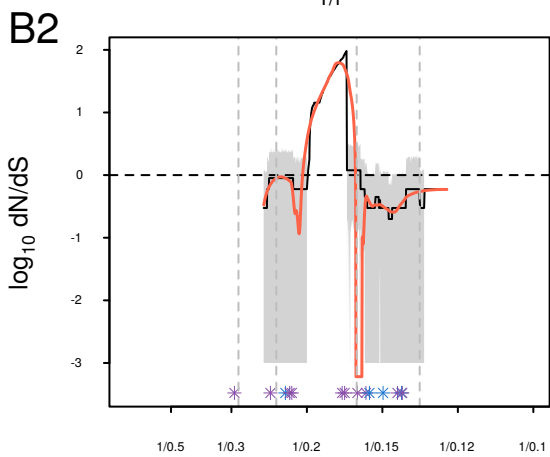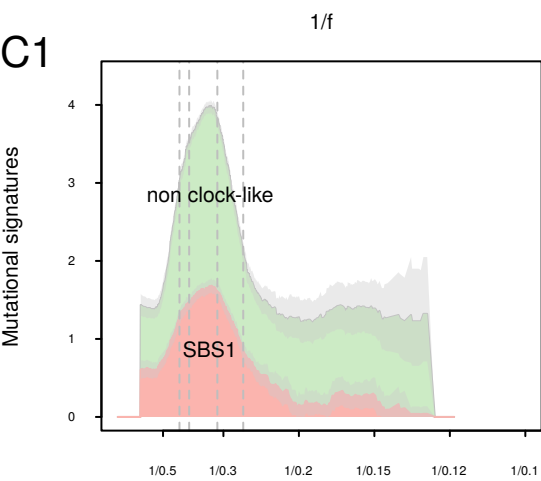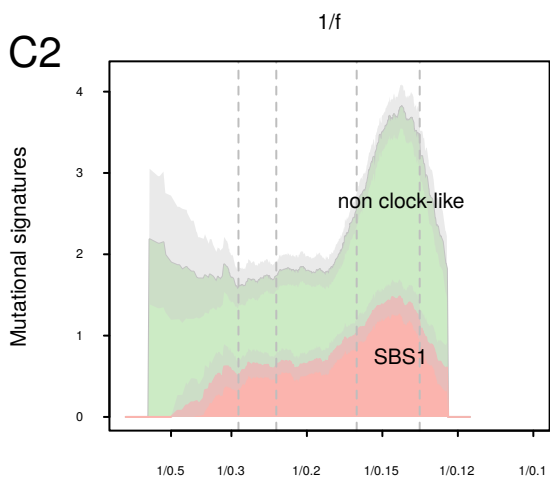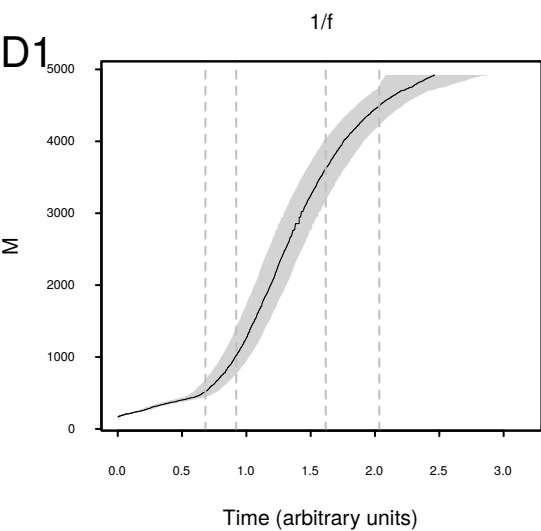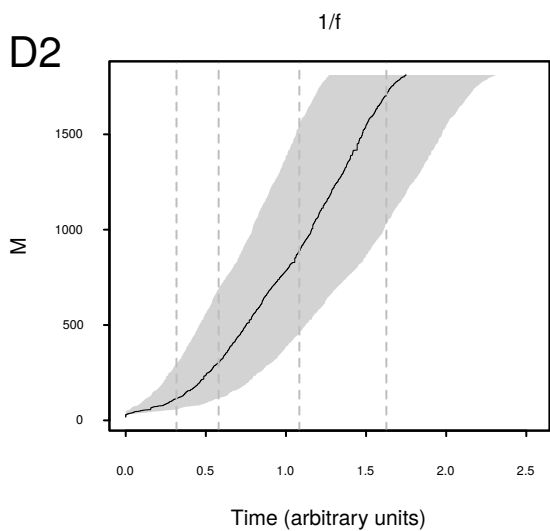

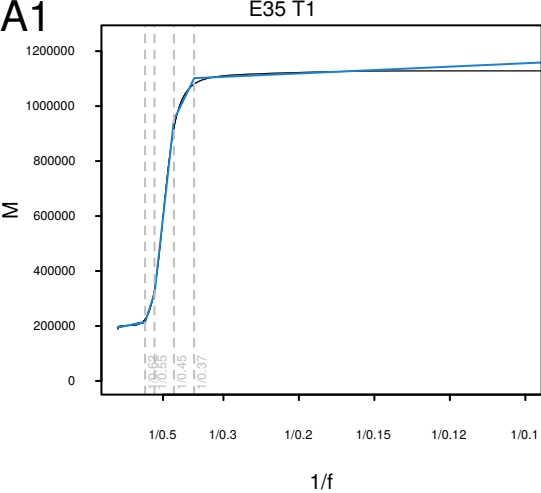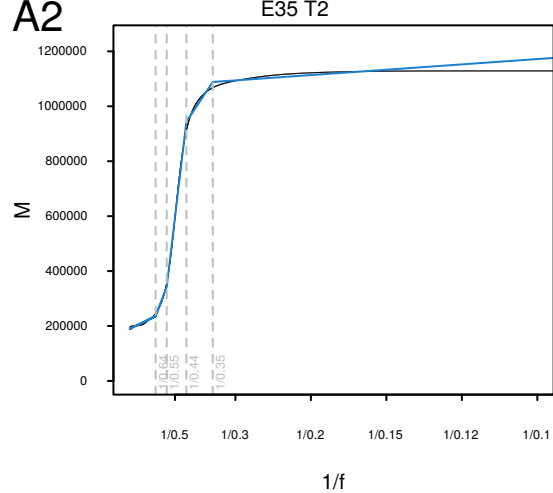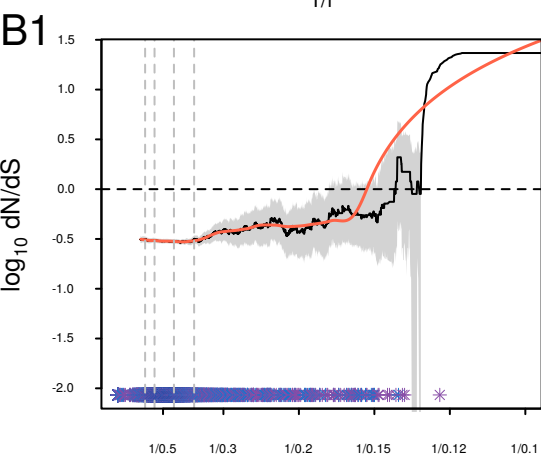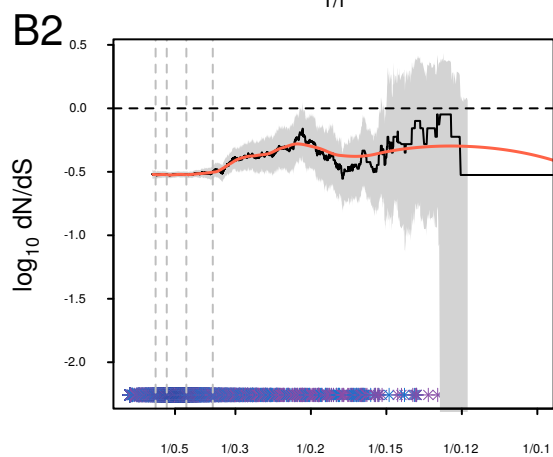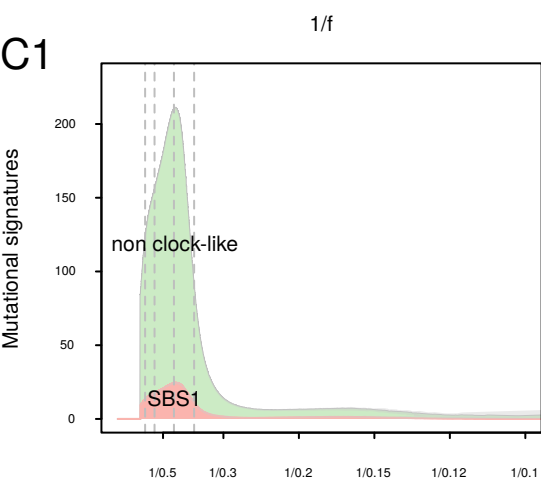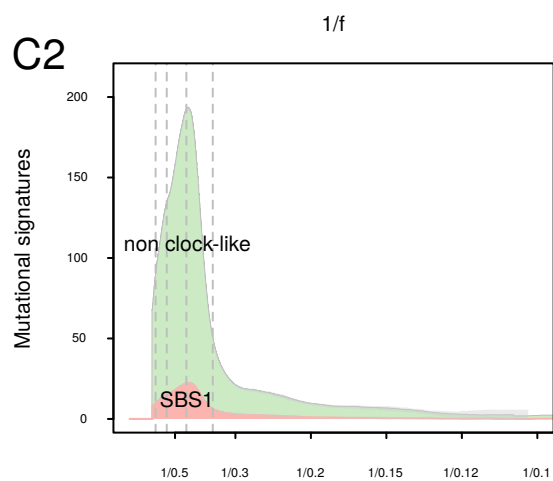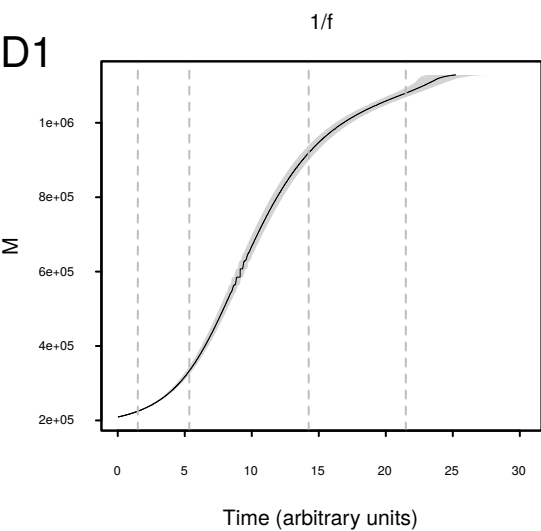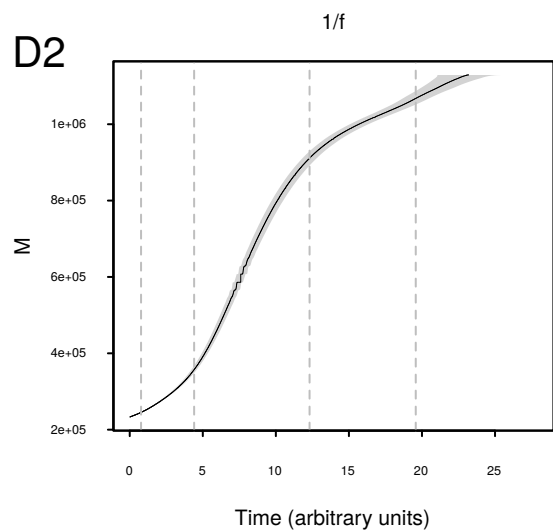

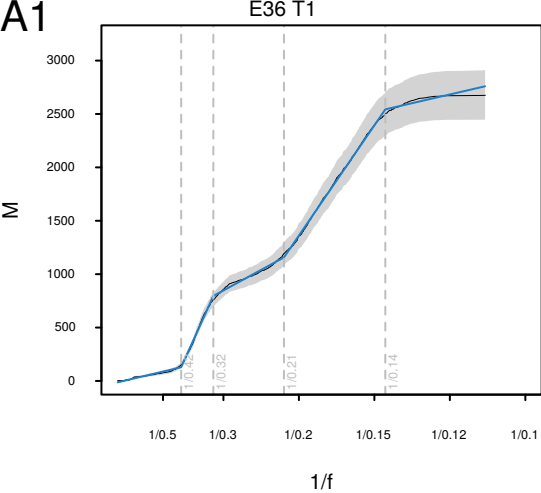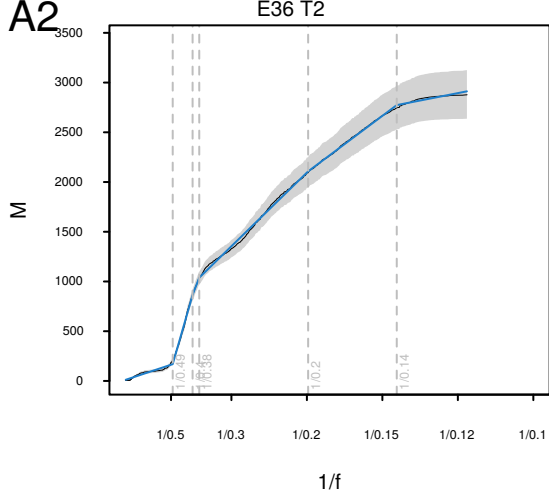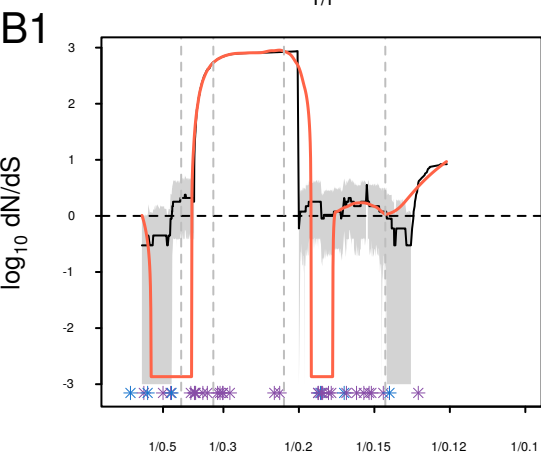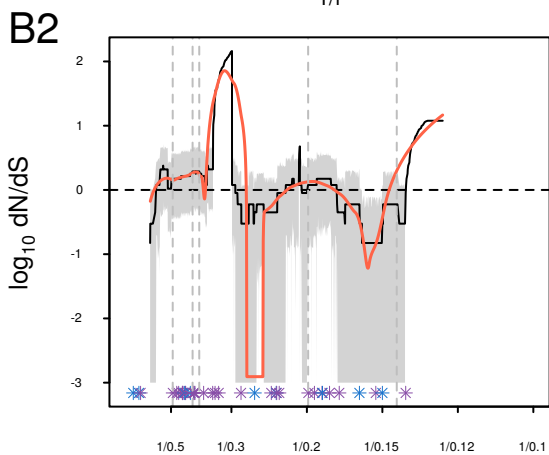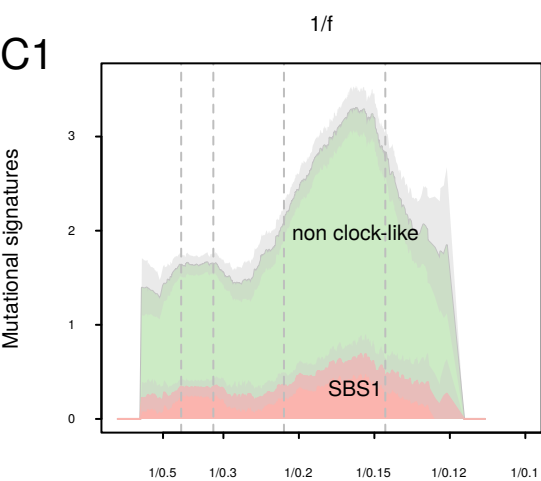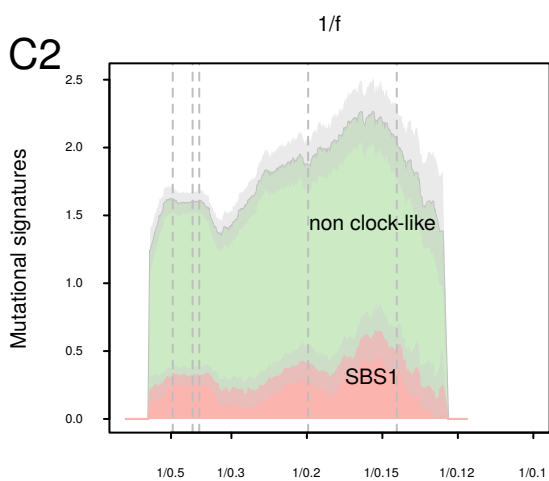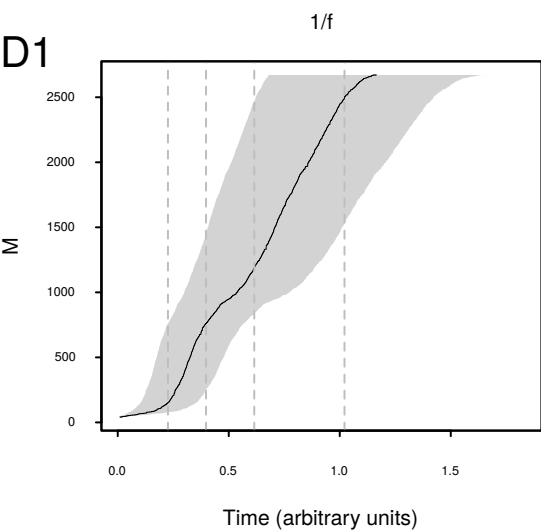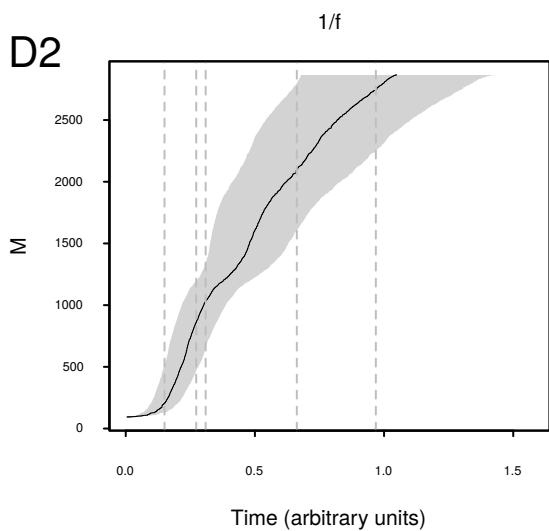

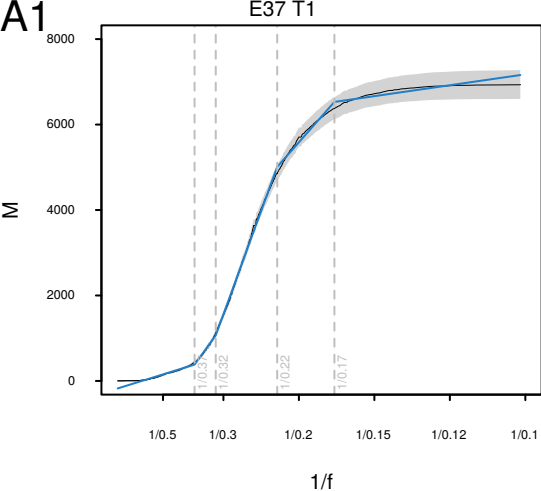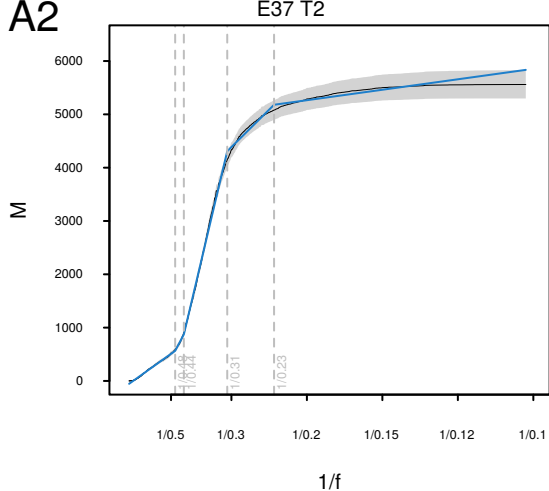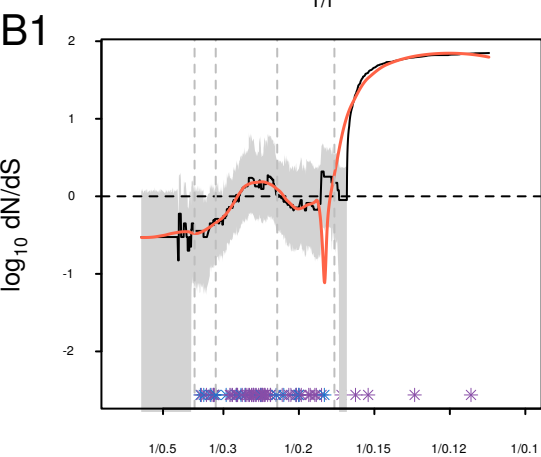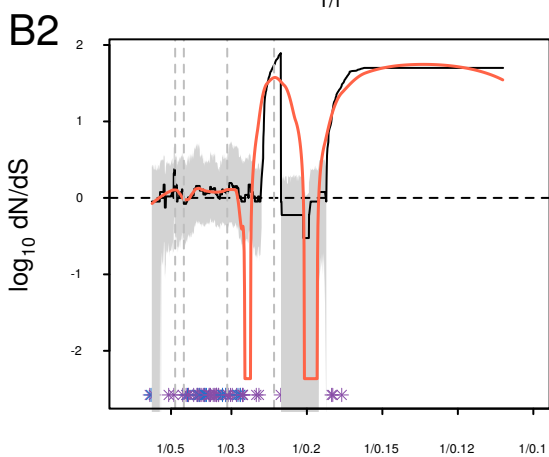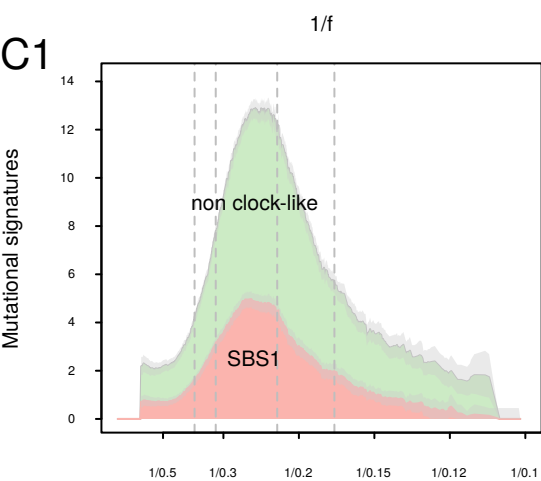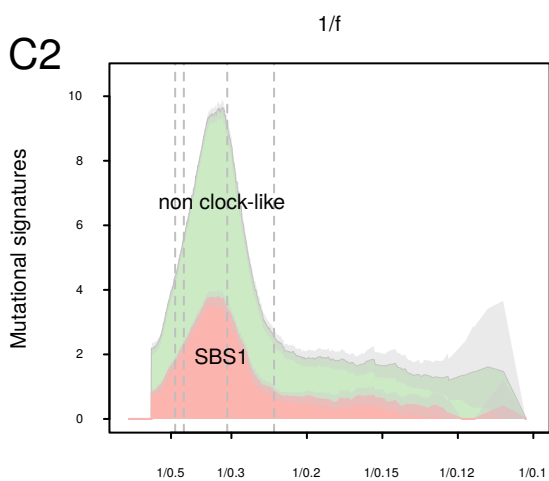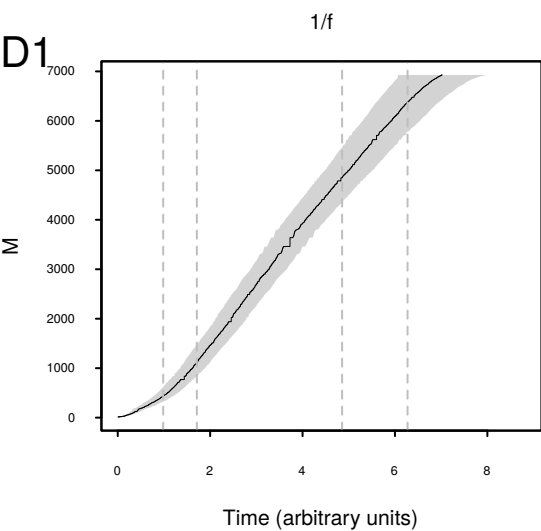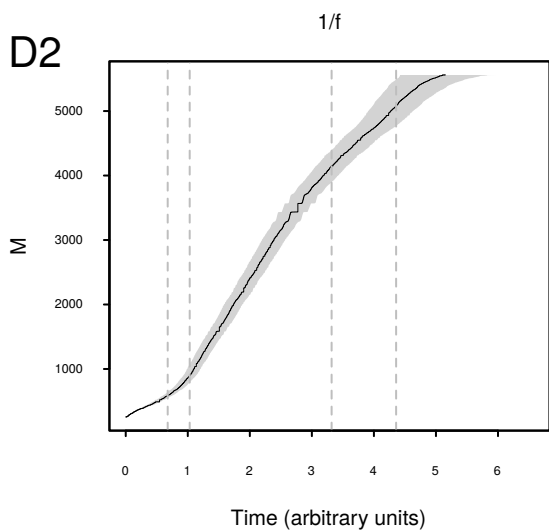

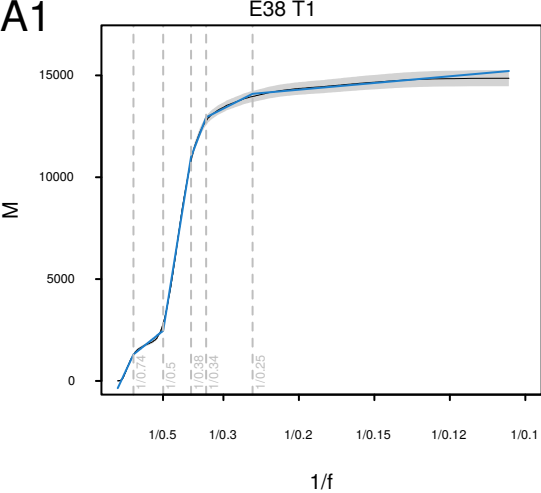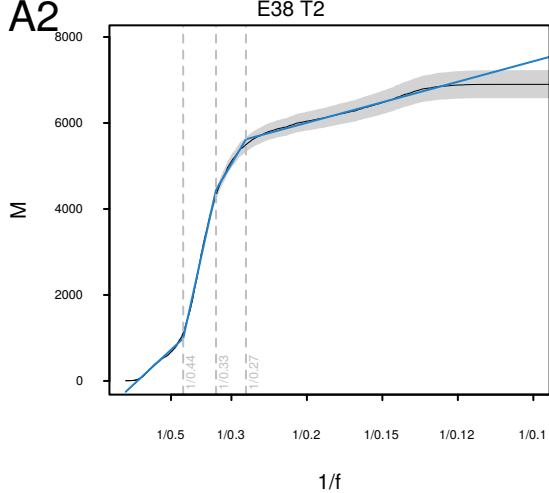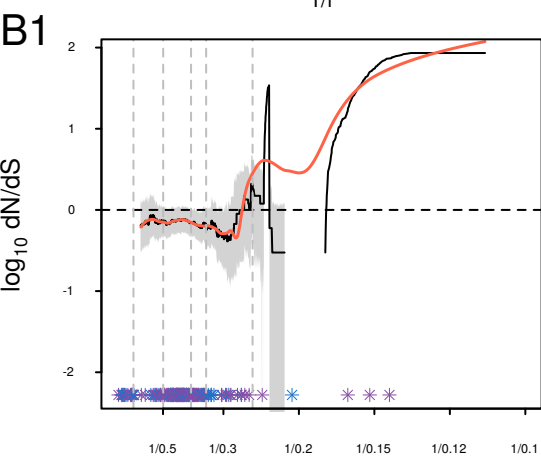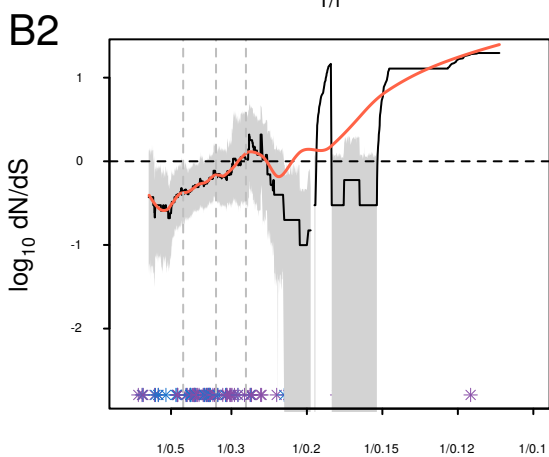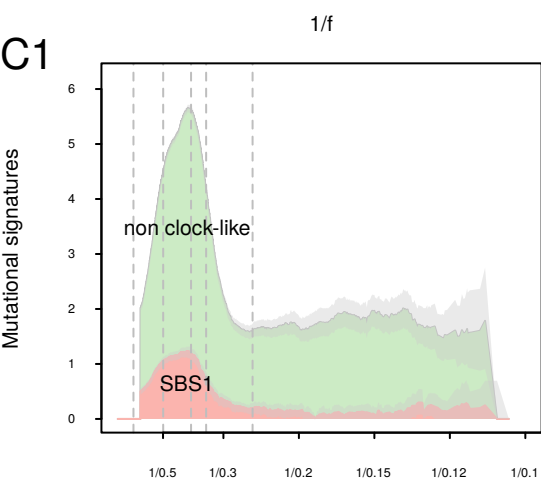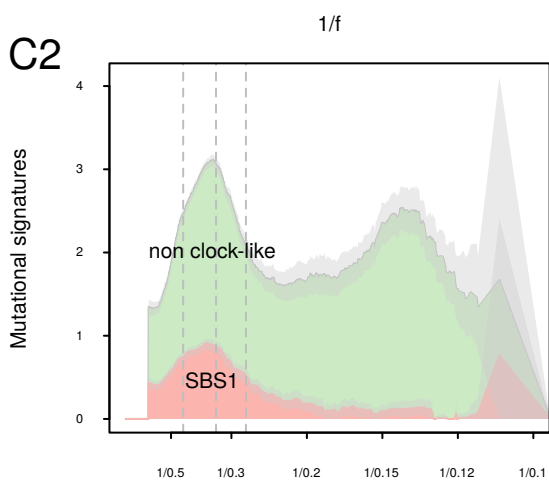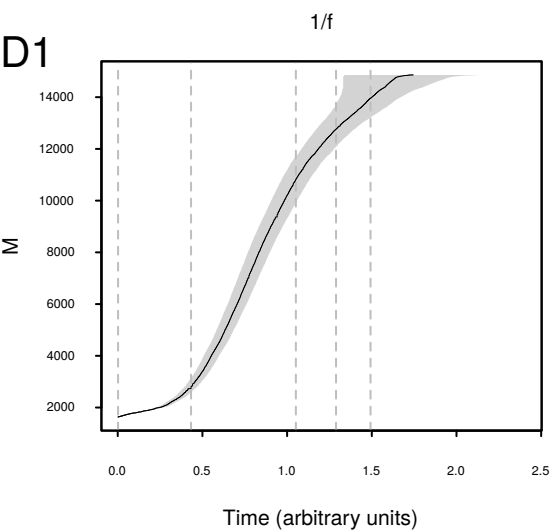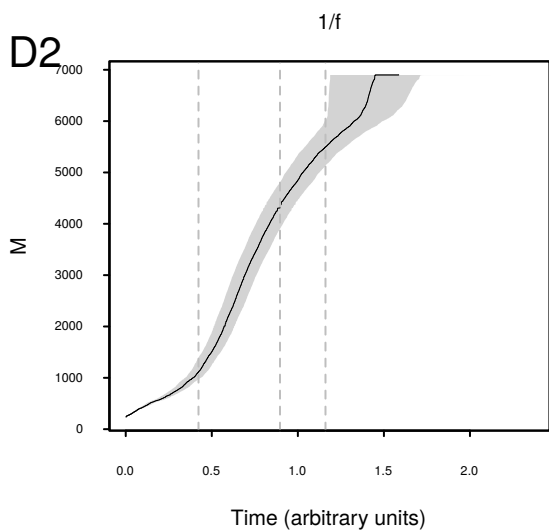

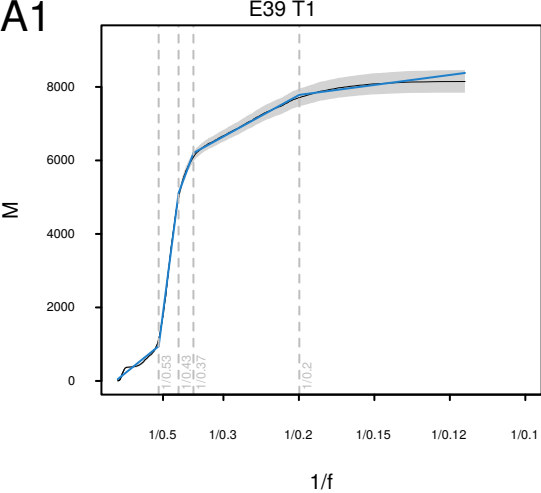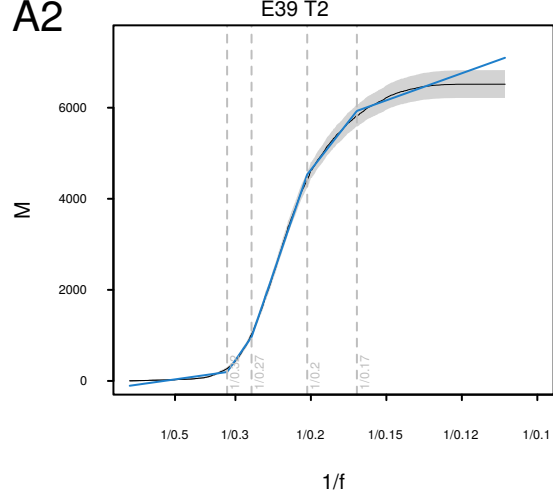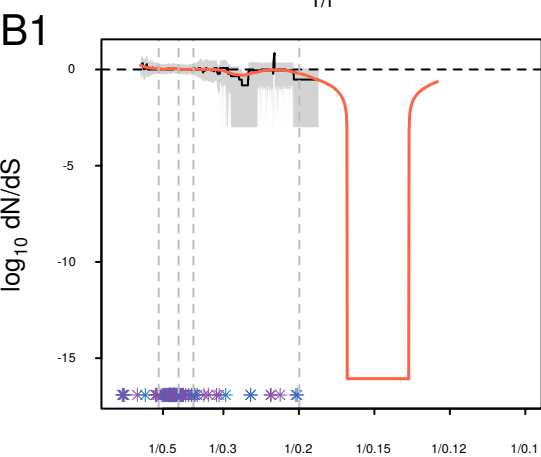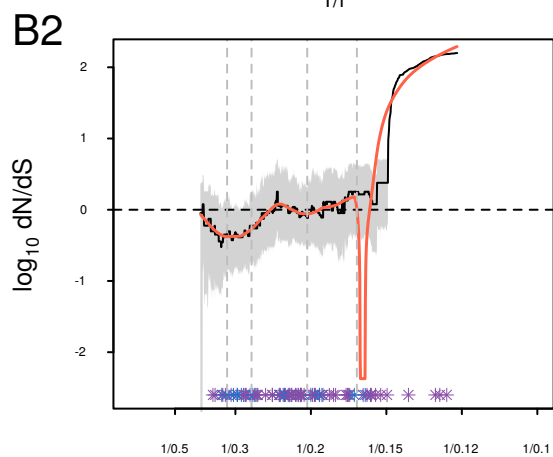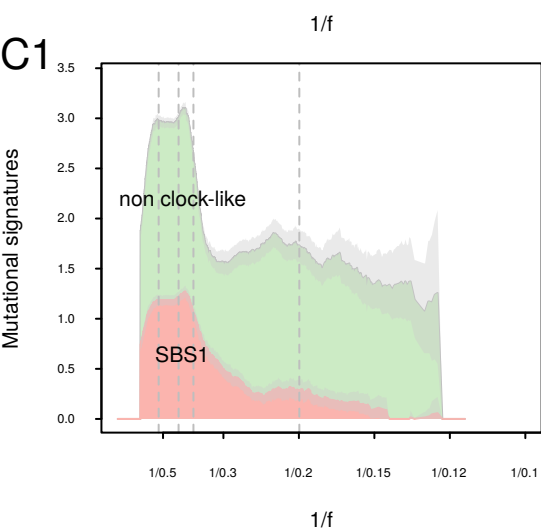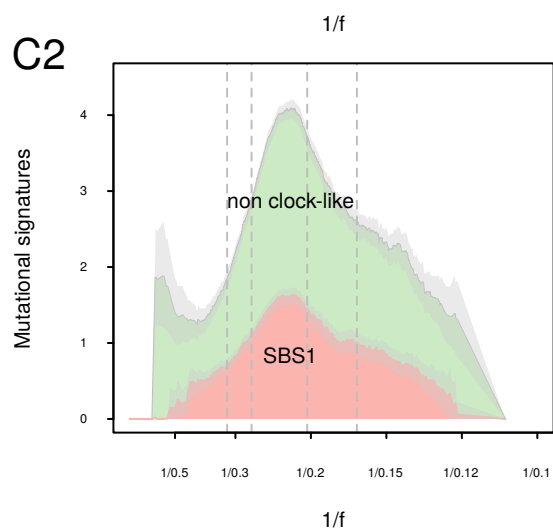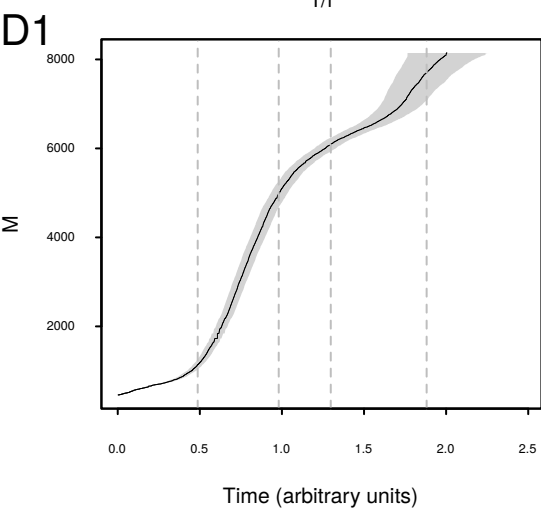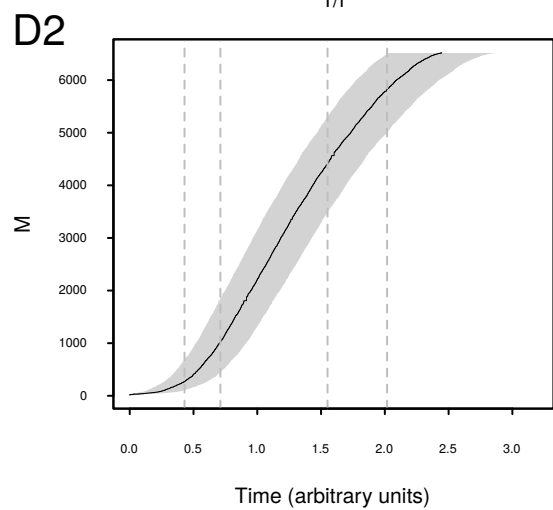

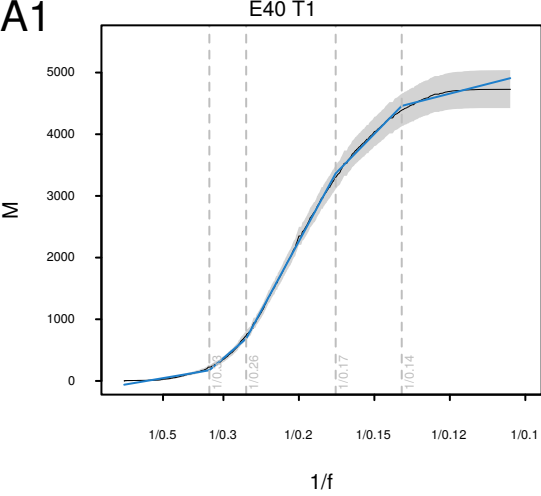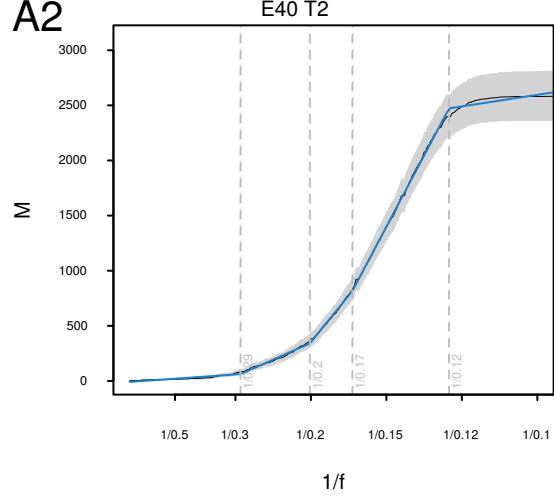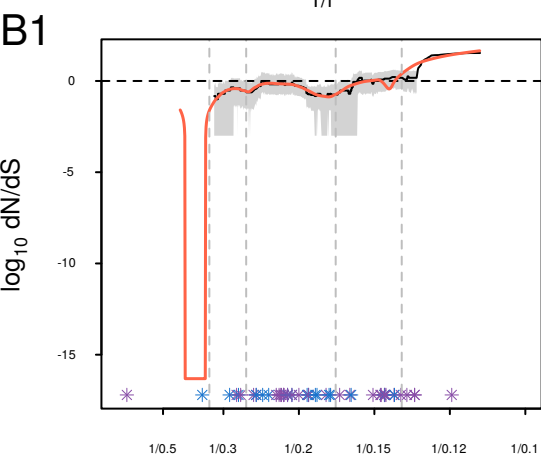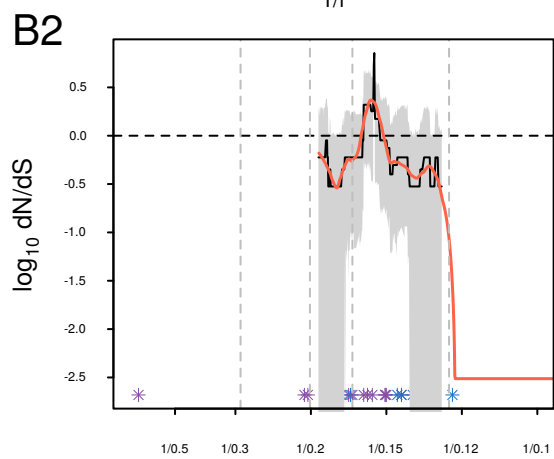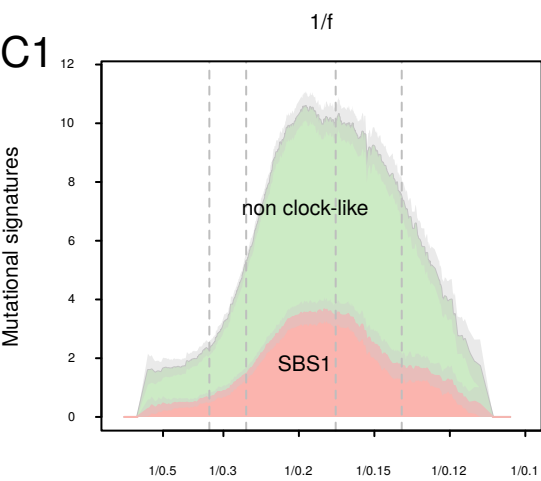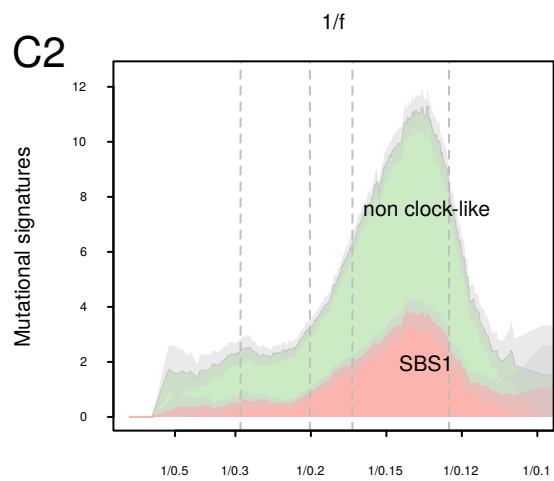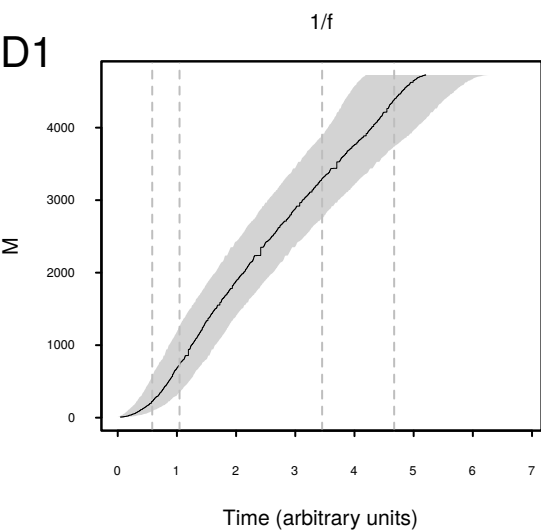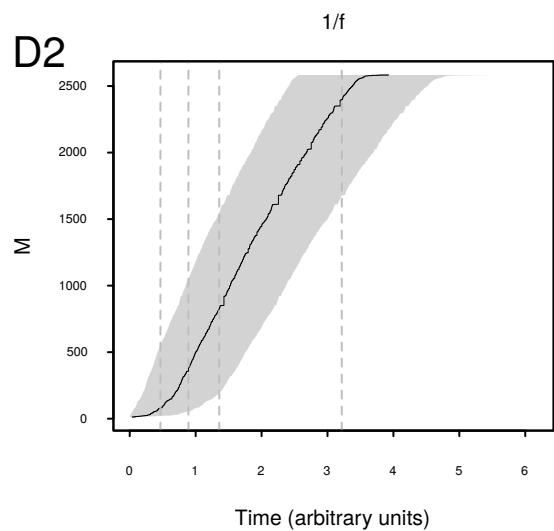

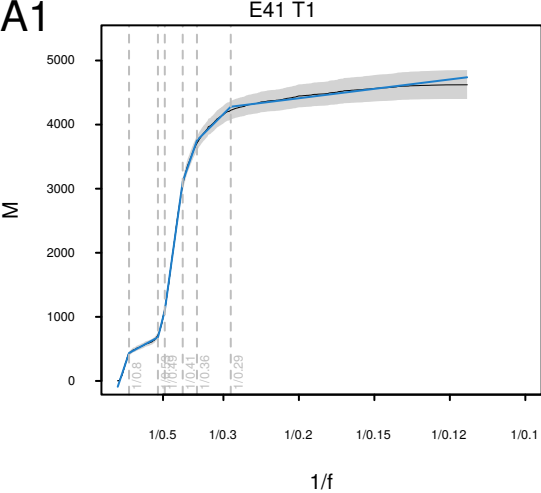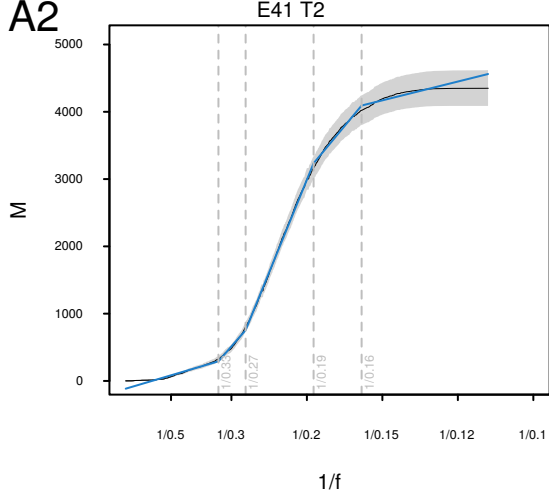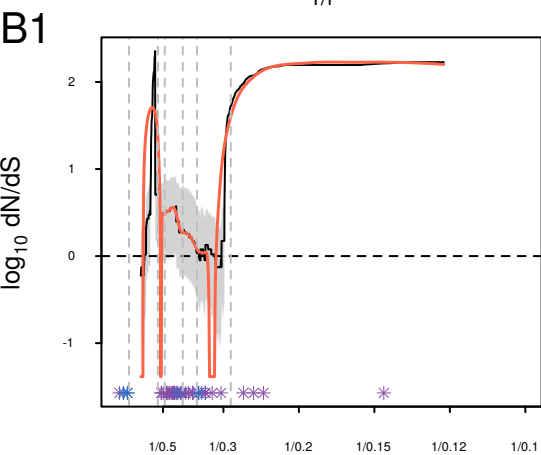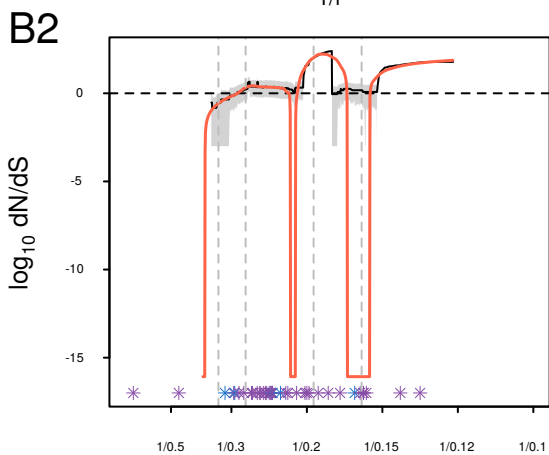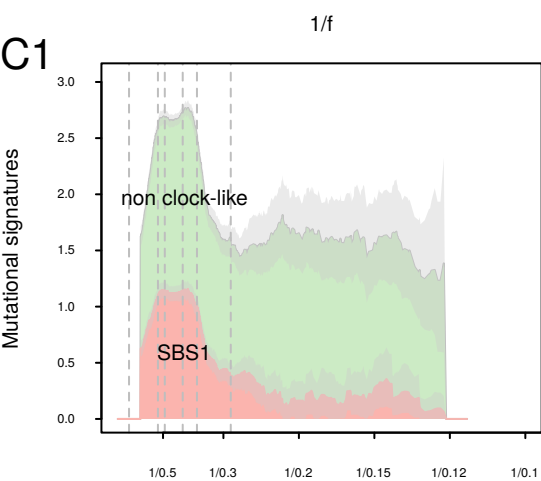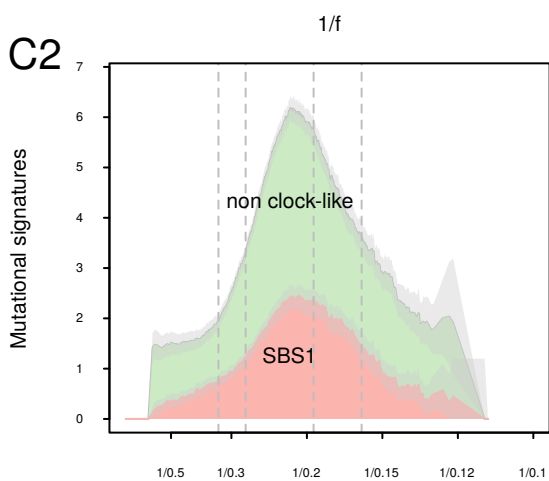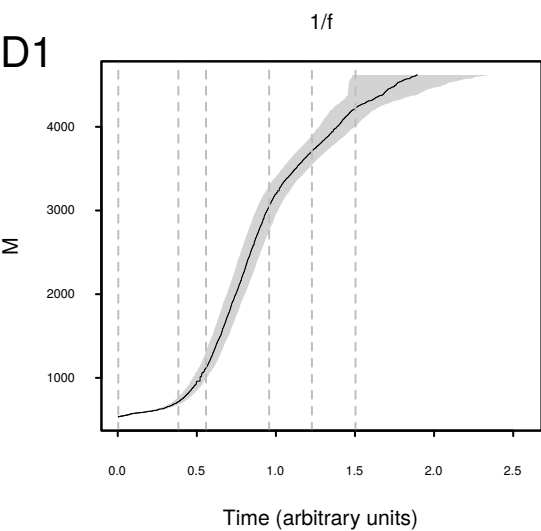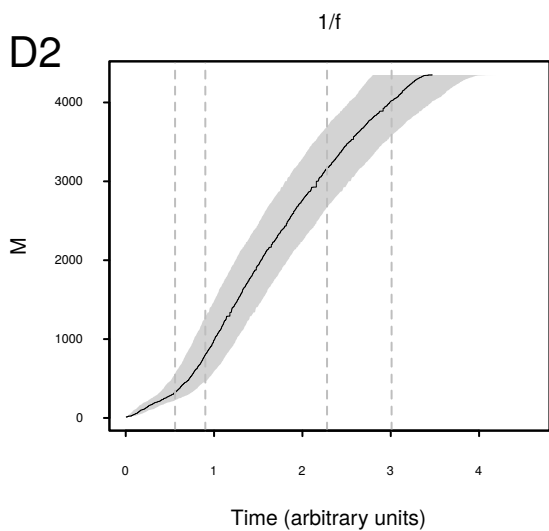

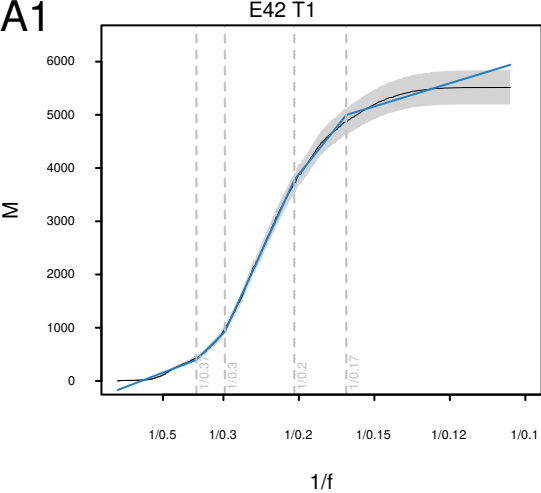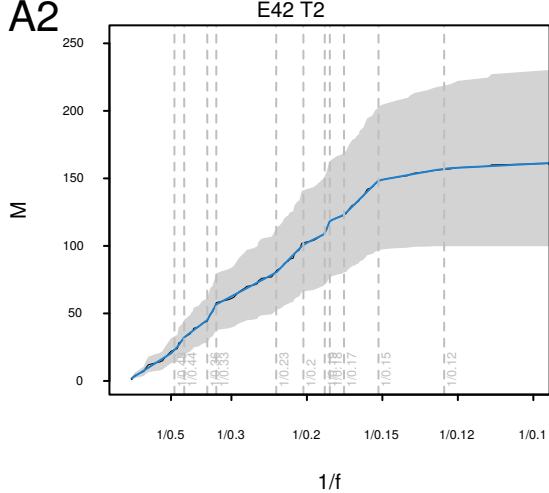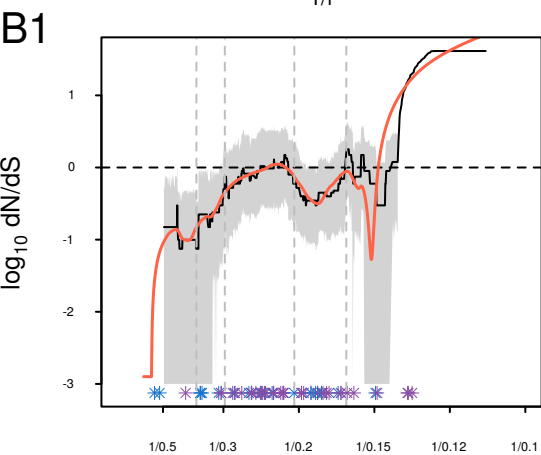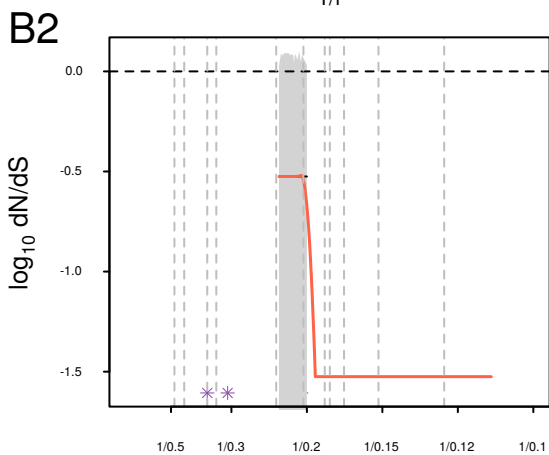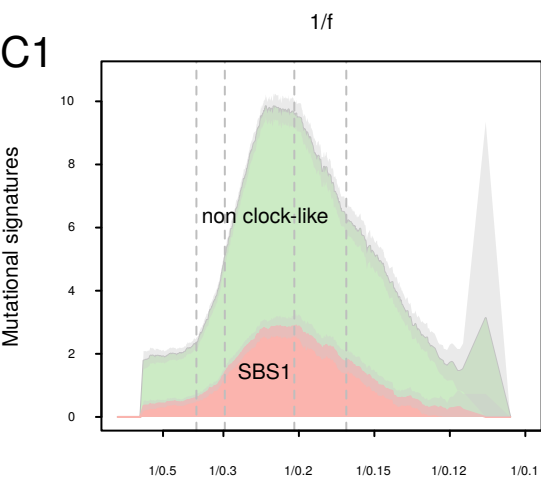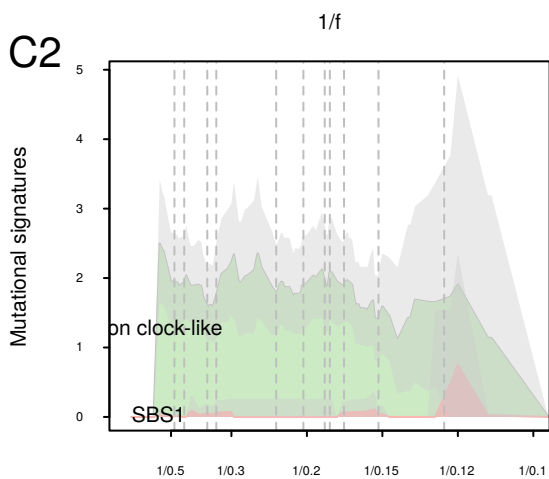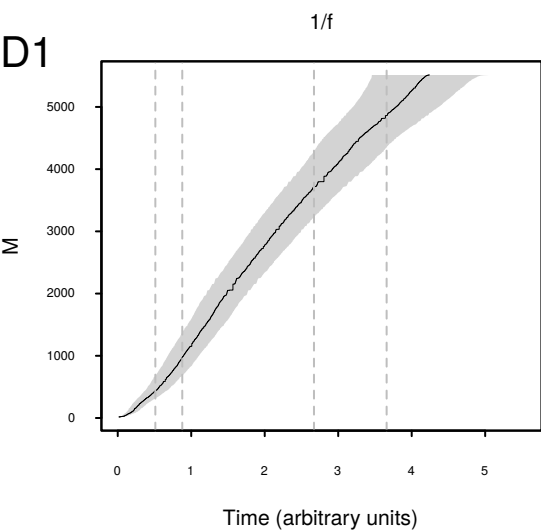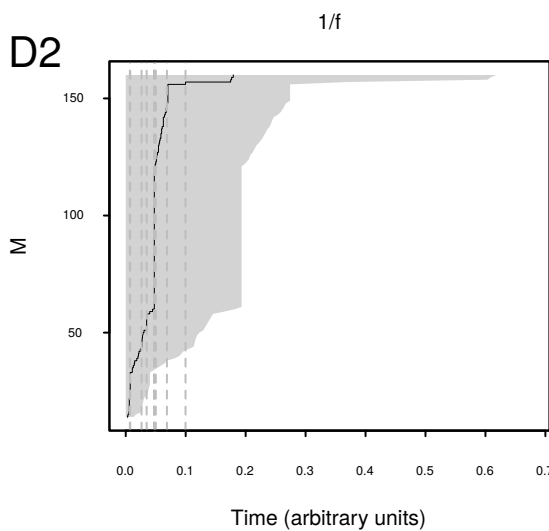

Supplement: S1 Fig — Panels A1-D1 show the primary tumor T1, panels A2-D2 show the recurrent tumor T2. (A) Mutation accumulation as a function of the inverse of allele frequency 1/f (black) and phases from automated segmentation (breakpoints (grey) and segments (blue)). The confidence band at 95% level is indicated in grey. (B) Nonsynonymous to synonymous ratio. Purple and blue stars show nonsynonymous and synonymous mutations, respectively. The smoothened ratio is shown in red. (C) Clock-like and non-clock-like mutational signatures. (D) Mutation accumulation as a function of time. (PDF) [file pgen.1011085.s001.pdf]
