# Supplementary material for: Time-resolved, integrated analysis of clonally evolving genomes
Source: PLoS Genet. 2023 Dec 14;19(12):e1011085. doi: 10.1371/journal.pgen.1011085 (PMC10754456; doi:10.1371/journal.pgen.1011085)
Supplement: S1 Text — (PDF) [file pgen.1011085.s003.pdf]

Supplementary Methods

Study of Mutation Accumulation

Contents

1 Expressing mutation accumulation as a function of time **2**
1.1 Mutation accumulation with no subclones 2
1.2 Mutation accumulation stratified by subclone 2

2 Expressing allele frequency as a function of time **2**
2.1 Number of cells and allele frequency 2
2.2 Number of cells and time 4
2.3 Allele frequency as a function of time 4

3 Expressing mutation accumulation as a function of allele frequency **5**
3.1 Mutation accumulation stratified by subclone 5
3.2 Mutation accumulation as a function of time for all subclones . . 5

1 Expressing mutation accumulation as a function of time

1.1 Mutation accumulation with no subclones

The number of mutations at time t is denoted $M(t)$. Taking relevant parameters in consideration, the increment of mutations $dM(t)$ at instant t can be expressed as follows:

$$dM(t) = \mu(t) \cdot \pi(t) \cdot \omega(t) \cdot \gamma(t) \cdot N(t) \cdot dt \ ,$$

where :

- $\mu(t)$ is the mutation rate at instant t, per division, per haploid genome,
- $\pi(t)$ is the ploidy at instant t,
- $\omega(t)$ is the number of divisions per unit time, at instant t,
- $\gamma(t)$ is the survival rate, at instant t,
- $N(t)$ is the number of cells at instant t.

The number of divisions per unit time is scaled by the number of effectively surviving cells, through the use of survival rate $\gamma(t)$. This is because only surviving cells give rise to observable mutations.

1.2 Mutation accumulation stratified by subclone

The expression in the latter section is limited to situations where cells are sufficiently homogeneous to be represented by a single set of parameters $\mu(t)$, $\pi(t)$, $\omega(t)$. This is seldom the case in tumors, where these parameters can vary from subclone to subclone or from cell to cell. Hence the increment of mutations is stratified by subclone i:

$$dM_i(t) = \mu_i(t) \cdot \pi_i(t) \cdot \omega_i(t) \cdot \gamma_i(t) \cdot N_i(t) \cdot dt \ , \qquad (1)$$

where i in $1, ..., n_c$ denotes a specific subclone, and n_c is the number of subclones.

2 Expressing allele frequency as a function of time

2.1 Number of cells and allele frequency

The number of cells, $N_i(t)$ can be expressed as a function of allele frequency $f_i(t)$ [1], when one considers that a mutation appearing in subclone i at instant t must correspond to one mutation among $N_i(t) \cdot \pi_i(t)$ chromosome copies, at that specific instant t. This can be written as follows:

$$f_i(t) = \frac{1}{N_i(t) \cdot \pi_i(t)} \ . \tag{2}$$

However, allele frequency is observed not only in subclone i, but over all subclones. Furthermore, allele frequency is observed not when the mutation occurred, but later at sample retrieval or resection time point, τ_r. It should be noted that τ_r is a constant.

Therefore we have to introduce a generalized version of equation (2), by using the observed allele frequency $f_i(t;\tau_r)$, for a mutation appeared in subclone i at time t and observed at time τ_r (resection time or sampling time).

To obtain the formulation of $f_i(t;\tau_r)$, we make the following assumptions :

- If a mutation is under selection, the subclone part containing the mutation is considered as an additional subclone. Inside of a subclone, selection is considered to be negligible.
- Growth is considered high enough to make random genetic drift negligible [2, 3, 4]. This allows to consider a constant allele frequency between mutation occurrence and sampling and sequencing events.
- Constant ploidy π.

Considering the above assumptions, the observed allele frequency $f_i(t;\tau_r)$ may be expressed as follows:

$$f_i(t;\tau_r) = \frac{1}{N_i(t) \cdot \pi} \cdot \frac{N_i(\tau_r)}{\sum\limits_j N_j(\tau_r)} \tag{3}$$

$$\iff N_i(t) = \frac{1}{f_i(t;\tau_r) \cdot \pi} \cdot \frac{N_i(\tau_r)}{\sum\limits_j N_j(\tau_r)} \ , \tag{4}$$

where j denotes a subclone, and maybe equal or different of i in 1,2,...,n_c.

The factor term $N_i(\tau_r)/\sum_j N_j(\tau_r)$ is denoted $K_{i,r}$ in the following. This factor corresponds to the proportion of subclone i among all subclones. $K_{i,r}$ is constant since it depends only on constant values.

Expressions (3) and (4) for the observed allele frequency and for number of cells are rewritten using $K_{i,r}$:

$$f_i(t;\tau_r) = \frac{K_{i,r}}{N_i(t) \cdot \pi} \tag{5}$$

$$\iff N_i(t) = \frac{K_{i,r}}{f_i(t;\tau_r) \cdot \pi} \ .$$

Finally, observed allele frequency $f_i(t;\tau_r)$ is comparable between subclones. As a result, it is possible to denote this frequency simply f, to represent the allele frequency observed at sampling time over all subclones, with t taken into account via $K_{i,r}$, and with τ_r, which is the same for all subclones, implied. For clarity we continue to use $f_i(t;\tau_r)$ when looking at a single subclone i, and we use f when summing over all subclones (section 3.2).

2.2 Number of cells and time

The new cells are generated via a certain number of divisions per time unit, equal to $\omega_i(t)$ and, since not all new cells survive, this number is modulated by the survival rate $\gamma_i(t)$. As a consequence, $Ni(t+dt)$ can be expressed as follows:

$$N_i(t+dt) = N_i(t) + \omega_i(t) \cdot \gamma_i(t) \cdot N_i(t) \cdot dt \ . \tag{6}$$

An increment of cells dN_i appeared during time increment dt is defined as follows:

$$dN_i = N_i(t+dt) - N_i(t) \ .$$

Consequently, dNi can be written as a function of dt, using (6):

$$dN_i = \omega_i(t) \cdot \gamma_i(t) \cdot N_i(t) \cdot dt \ . \tag{7}$$

2.3 Allele frequency as a function of time

From here, we use inverse allele frequency $1/f_i(t;\tau_r)$ and its increment $d(1/f_i(t;\tau_r))$, instead of $f_i(t;\tau_r)$ and $df_i(t;\tau_r)$, because it is equivalent and yields simpler formulations. From the above expression (5), $1/f_i(t;\tau_r)$ can be expressed as a function of $N_i(t)$:

$$(1/f_i)(t;\tau_r) = \frac{N_i(t) \cdot \pi}{K_{i,r}} \ .$$

Doing the same at $t+dt$ and substracting the expression at t from the expression at $t+dt$ yields the increment:

$$\begin{aligned} d(1/f_i(t;\tau_r)) &= (1/f_i)(t+dt;\tau_r) - (1/f_i)(t;\tau_r) \\ &= \frac{N_i(t+dt) \cdot \pi}{K_{i,r}} - \frac{N_i(t) \cdot \pi}{K_{i,r}} \\ &= \frac{(N_i(t+dt) - N_i(t)) \cdot \pi}{K_{i,r}} \\ &= \frac{dN_i \cdot \pi}{K_{i,r}} \ . \end{aligned}$$

Then, dN_i can be replaced by its expression (7), leading to the following:

$$d(1/f_i(t;\tau_r)) = \frac{\omega_i(t) \cdot \gamma_i(t) \cdot N_i(t) \cdot dt \cdot \pi}{K_{i,r}} \ . \tag{8}$$

These equations, as well as those for the number of cells dynamics, are summarized in table 1.

3 Expressing mutation accumulation as a function of allele frequency

3.1 Mutation accumulation stratified by subclone

Using equation (8), one can reformulate mutation accumulation from (1) as a function of $d(1/f)$:

Table 1: Dynamics of $N_i(t)$ and $(1/f)(t)$.

	Value at t	Value at $t+dt$	Increment during dt
N_i	$N_i(t)$	$N_i(t+dt) = N_i(t) + \omega_i(t)\,\gamma_i(t)\,N_i(t)\,dt$	$N_i(t+dt) - N_i(t) = dN_i = \omega_i(t)\,\gamma_i(t)\,N_i(t)\,dt$
$1/f$	$(1/f_i)(t;\tau_r) = \frac{N_i(t)\,\pi}{K_{i,r}}$	$(1/f_i)(t+dt;\tau_r) = \frac{N_i(t+dt)\,\pi}{K_{i,r}}$	$(1/f_i)(t+dt;\tau_r) - (1/f_i)(t;\tau_r) = d(1/f)(t) = \frac{(N_i(t+dt)-N_i(t))\,\pi}{K_{i,r}} = \frac{dN_i \cdot \pi}{K_{i,r}} = \frac{\pi\,\omega_i(t)\,\gamma_i(t)\,N_i(t)\,dt}{K_{i,r}}$

$$dM_i(t) = \mu_i(t) \cdot \pi \cdot \omega_i(t) \cdot \gamma_i(t) \cdot N_i(t) \cdot dt \cdot \frac{d(1/f_i)(t;\tau_r) \cdot K_{i,r}}{\omega_i(t) \cdot \gamma_i(t) \cdot N_i(t) \cdot dt \cdot \pi} \ . \quad (9)$$

Equation (9) simplifies into the following:

$$dM_i(t) = \mu_i(t) \cdot K_{i,r} \cdot d(1/f_i)(t;\tau_r) \ . \quad (10)$$

3.2 Mutation accumulation as a function of time for all subclones

Considering all subclones, mutation accumulation is simply the sum over subclones 1, ... , i, ... , n_c of the mutation increment in each subclone:

$$dM(t) = \sum_{i=1}^{n_c} dM_i(t) \ , \quad (11)$$

Using (10) and (11) yields mutation accumulation as a function of the inverse allele frequency (12). As mentioned in section *2.1*, f is the observed allele frequency and is identical from one subclone to the other, while discrepancies between subclones are represented by constant $K_{i,r}$. Mutation accumulation over all subclones can then be written as follows:

$$dM(t) = \left(\sum_{i=1}^{n_c} \mu_i(t) \cdot K_{i,r} \right) \cdot d(1/f) \ . \quad (12)$$

From equation (12), mutation accumulation dM is proportional to $d(1/f)$. This relation is modulated by a weighted sum over the mutation rates in subclones i, where the weights are the $K_{i,r}$ terms. Since the weights $K_{i,r}$ do not

depend on time, this means that constant mutation rates lead to a linear relationship between dM and $d(1/f)$, regardless of the presence or not of natural selection.

Conversely, it is not possible to say that linearity between dM and $d(1/f)$ implies constant mutation rates, since these terms could in principle exactly compensate each other to form the same constant sum $\sum_i \mu_i(t) \cdot K_{i,r}$. However, this might be considered unlikely.

As a result, one can write the following equivalence, implication and consideration:

$$\left\{ \sum_i \mu_i(t) \cdot K_{i,r} \text{ } \textit{is constant} \right\} \iff \{ M(1/f) \text{ } \textit{is linear} \} \,,$$

$$\{ \mu_i(t) \text{ } \textit{is constant} \ \forall i \} \Rightarrow \{ M(1/f) \text{ } \textit{is linear} \}$$

and

$$\{ M(1/f) \text{ } \textit{is linear} \} \text{ } \textit{possibly implies} : \{ \mu_i(t) \text{ } \textit{is constant} \ \forall i \} \,.$$

As a consequence, equation (12) also means that the profile of M as a function of $1/f$ corresponds to a sum of subclones at each $1/f$. As a consequence, any nonlinearity in this curve will reflect mutation rate dynamics happening in one (or several) subclone(s), and does not correspond to a single subclone.

References

[1] Marc J Williams, Benjamin Werner, Chris P Barnes, Trevor A Graham, and Andrea Sottoriva. Identification of neutral tumor evolution across cancer types. *Nat. Genet.*, 48(3):238–244, 2016.

[2] Ivana Bozic, Jeffrey M. Gerold, and Martin A. Nowak. Quantifying clonal and subclonal passenger mutations in cancer evolution. *PLoS Comput. Biol.*, 12(2):e1004731, 2013.

[3] Hisashi Ohtsuki and Hideki Innan. Forward and backward evolutionary processes and allele frequency spectrum in a cancer cell population. *Theor. Popul. Biol.*, 117:43–50, 2017.

[4] Yuxin Chen, Ding Tong, and Chung-I Wu. A new formulation of random genetic drift and its application to the evolution of cell populations. *Mol. Biol. Evol.*, 34(8):2057–2064, 2017.
