# Supplementary material for: Time-resolved, integrated analysis of clonally evolving genomes
Source: PLoS Genet. 2023 Dec 14;19(12):e1011085. doi: 10.1371/journal.pgen.1011085 (PMC10754456; doi:10.1371/journal.pgen.1011085)
Supplement: S1 Table — P-values for test of difference between segments for P. virginalis and glioblastoma samples. (DOCX) [file pgen.1011085.s004.docx]

**Supplementary Table 1.** *P-values* for test of difference between segments for P. virginalis and glioblastoma samples.

| **Sample** | ***p-value* (for primary tumor for glioblastoma samples)** | ***p-value* for recurrent tumor (for glioblastoma samples)** |
| --- | --- | --- |
| P.virginalis | 6.345498x10^-2^ | not applicable |
| GBM1 | <2.2x10^-16^ | <2.2x10^-16^ |
| GBM2 | <2.2x10^-16^ | <2.2x10^-16^ |
| GBM3 | <2.2x10^-16^ | <2.2x10^-16^ |
| GBM4 | <2.2x10^-16^ | <2.2x10^-16^ |
| GBM5 | <2.2x10^-16^ | <2.2x10^-16^ |
| GBM6 | <2.2x10^-16^ | <2.2x10^-16^ |
| GBM7 | <2.2x10^-16^ | <2.2x10^-16^ |
| GBM8 | <2.2x10^-16^ | <2.2x10^-16^ |
| GBM9 | <2.2x10^-16^ | <2.2x10^-16^ |
| GBM10 | <2.2x10^-16^ | <2.2x10^-16^ |
| GBM11 | <2.2x10^-16^ | <2.2x10^-16^ |
| GBM12 | <2.2x10^-16^ | <2.2x10^-16^ |
| GBM13 | <2.2x10^-16^ | <2.2x10^-16^ |
| GBM14 | <2.2x10^-16^ | <2.2x10^-16^ |
| GBM15 | 7.962633 x10^-15^ | <2.2x10^-16^ |
| GBM16 | <2.2x10^-16^ | <2.2x10^-16^ |
| GBM17 | <2.2x10^-16^ | 7.891463 x10^-01^ |
| GBM18 | <2.2x10^-16^ | <2.2x10^-16^ |
| GBM19 | <2.2x10^-16^ | <2.2x10^-16^ |
| GBM20 | <2.2x10^-16^ | <2.2x10^-16^ |
| GBM21 | <2.2x10^-16^ | <2.2x10^-16^ |
| GBM22 | <2.2x10^-16^ | 1.883298 x10^-03^ |
| GBM23 | <2.2x10^-16^ | 1.000000 |
| GBM24 | <2.2x10^-16^ | 4.886992 x10^-11^ |
| GBM25 | <2.2x10^-16^ | 2.928589 x10^-02^ |
| GBM26 | <2.2x10^-16^ | <2.2x10^-16^ |
| GBM27 | <2.2x10^-16^ | <2.2x10^-16^ |
| GBM28 | <2.2x10^-16^ | <2.2x10^-16^ |
| GBM29 | <2.2x10^-16^ | <2.2x10^-16^ |
| GBM30 | 0.000000 | <2.2x10^-16^ |
| GBM31 | <2.2x10^-16^ | <2.2x10^-16^ |
| GBM32 | <2.2x10^-16^ | <2.2x10^-16^ |
| GBM33 | <2.2x10^-16^ | <2.2x10^-16^ |
| GBM34 | <2.2x10^-16^ | <2.2x10^-16^ |
| GBM35 | <2.2x10^-16^ | <2.2x10^-16^ |
| GBM36 | <2.2x10^-16^ | <2.2x10^-16^ |
| GBM37 | <2.2x10^-16^ | <2.2x10^-16^ |
| GBM38 | <2.2x10^-16^ | <2.2x10^-16^ |
| GBM39 | <2.2x10^-16^ | <2.2x10^-16^ |
| GBM40 | <2.2x10^-16^ | <2.2x10^-16^ |
| GBM41 | <2.2x10^-16^ | <2.2x10^-16^ |
| GBM42 | <2.2x10^-16^ | 5.637243 x10^-04^ |
