# Supplementary material for: Time-resolved, integrated analysis of clonally evolving genomes
Source: PLoS Genet. 2023 Dec 14;19(12):e1011085. doi: 10.1371/journal.pgen.1011085 (PMC10754456; doi:10.1371/journal.pgen.1011085)
Supplement: S2 Table — Characteristics of tumor cell survival ratio γR/γP (n = 20). (DOCX) [file pgen.1011085.s005.docx]

**Supplementary Table 2.** Characteristics of tumor cell survival ratio γ_R_/γ_P_ (n=20). Index R denotes the start of the recurrence and P the end of the primary. The lower and higher bounds on γ_R_/γ_P_ correspond to tumor emergence 2 and 7 years before diagnosis, respectively.

|  | log γ_R_/γ_P_ | |
| --- | --- | --- |
| **sample** | **lower bound** | **higher bound** |
| 1 | 1.9782320 | 2.5223001 |
| 4 | 3.3990271 | 3.9430951 |
| 6 | 1.3989074 | 1.9429809 |
| 7 | 1.5080083 | 2.0520764 |
| 8 | 1.2397434 | 1.7838077 |
| 10 | 1.4497716 | 1.9938336 |
| 11 | 3.2291480 | 3.7746907 |
| 14 | 1.4457411 | 1.9898273 |
| 15 | 1.2063772 | 1.7504348 |
| 18 | 1.4437458 | 1.9877957 |
| 21 | 1.6193366 | 2.1634227 |
| 23 | 2.2465702 | 2.7907532 |
| 24 | 1.3218368 | 1.8659186 |
| 27 | 2.3521282 | 2.8961963 |
| 28 | 1.8456369 | 2.3896593 |
| 30 | 1.0004823 | 1.5445460 |
| 36 | 1.2751182 | 1.8191862 |
| 39 | 0.0260095 | 0.5700782 |
| 40 | 0.7027364 | 1.2468078 |
| 42 | 0.5513292 | 1.0953980 |
| **mean** | 1.561994 | 2.106140 |
| **95% CI** | [1.250896 ; 1.947115] | [1.795669 ; 2.490084] |
| **median** | 1.444743 | 1.988812 |
| **95% CI** | [1.240748 ; 1.732487] | [1.784811 ; 2.276541] |
