# Supplementary material for: Time-resolved, integrated analysis of clonally evolving genomes
Source: PLoS Genet. 2023 Dec 14;19(12):e1011085. doi: 10.1371/journal.pgen.1011085 (PMC10754456; doi:10.1371/journal.pgen.1011085)
Supplement: S3 Table — Procambarus virginalis Samples. (DOCX) [file pgen.1011085.s006.docx]

**Supplementary Table 3.** *Procambarus virginalis* Samples

| **Name** | **Date of birth (y)** | **Uncertainty (y)** | **Name in Gutekunst *et al.* 2018** |
| --- | --- | --- | --- |
| Animal 1 | 2012.12500 | 0.166667 | Petshop 1 laboratory strain |
| Animal 2 | 2014.87500 | 0.083333 | Petshop 2 laboratory strain |
| Animal 34 | 2017.87500 | 0.083333 | n.a. |
| Animal 35 | 2018.20833 | 0.083333 | n.a. |
| Hannover | 2016.95833 | 0.083333 | Hannover aquarium lineage |
| Heidelberg | 2010.00000 | 2.000000 | Heidelberg laboratory strain |
| Madagascar 1 | 2011.00000 | 1.000000 | MA1 |
| Madagascar 2 | 2015.25000 | 0.250000 | MA2 |
| Madagascar 3 | 2015.58333 | 0.166667 | MA3 |
| Madagascar 4 | 2015.58333 | 0.250000 | MA4 |
| Madagascar 5 | 2015.58333 | 0.166667 | MA5 |
| Moosweiher | 2011.00000 | 1.000000 | Moosweiher |
| Reilingen | 2015.00000 | 1.000000 | Reilingen |

n.a. : not applicable
