# Supplementary material for: Time-resolved, integrated analysis of clonally evolving genomes
Source: PLoS Genet. 2023 Dec 14;19(12):e1011085. doi: 10.1371/journal.pgen.1011085 (PMC10754456; doi:10.1371/journal.pgen.1011085)
Supplement: S4 Table — Raw sequencing data for new and resequenced Procambarus virginalis samples. (DOCX) [file pgen.1011085.s007.docx]

**Supplementary Table 4.** Raw sequencing data for new and resequenced *Procambarus virginalis* samples

| **Sample** | **Sequencing strategy** | **Read pairs [Mio]** | **Genome coverage per strand** |
| --- | --- | --- | --- |
| Animal 34 | HISEQX Paired-end 150 bp | 382.6 | 16.4x |
| Animal 35 | HISEQX Paired-end 150 bp | 399.7 | 17.1x |
| Madagascar 1 | HISEQX Paired-end 150 bp | 408.7 | 17.5x |
| Moosweiher | HISEQX Paired-end 150 bp | 420.5 | 18.0x |
