# Supplementary material for: Time-resolved, integrated analysis of clonally evolving genomes
Source: PLoS Genet. 2023 Dec 14;19(12):e1011085. doi: 10.1371/journal.pgen.1011085 (PMC10754456; doi:10.1371/journal.pgen.1011085)
Supplement: Attachment — Submitted filename: PGENETICS-D-23-00320R1_Response-to-reviewers.pdf [file pgen.1011085.s009.pdf]

Reviewer #1

In this manuscript, Legrand and colleagues develop a framework to study mutation accumulation in asexual organisms, in which they look at the relationship between mutation accumulation and variant allele frequency as a proxy for mutational age. This allows disentangling the links between mutation rate, tissue growth and survival of de novo mutations. Authors apply their framework in both a seemingly young parthenogenetic species (the marbled crayfish Procambarus virginalis) and clonally-evolving tissues (glioblastoma), analyzing previously published data and some newly sequenced crayfish sequences. My background is in empirical population genomics and I have some experience working with non-model parthenogenetic species. My expertise is very limited regarding cancer evolution. As a result, my comments below reflect my expertise (or lack thereof) on these different aspects. Overall, I enjoyed reading the paper, from what I can tell the approach is original and mostly sound and the analyses are rigorous (but see my first major comment below). I however believe that the authors could do a better job at demonstrating the importance of their findings and its broad interest to the PLOS Genetics audience (see major comment 2).
>> We thank the reviewer for highlighting the originality of our manuscript and have made numerous changes to address major points 1 and 2 (see details below).

Major comment 1 – The framework heavily relies on a precise estimation of the mutation rate, which is a complex endeavor for non-model organisms. Thankfully, authors can rely on a marbled crayfish line which was maintained in the lab over seven years (2012-2019), basically providing a mutation accumulation experiment. Authors used whole-genome sequencing of three individuals sampled in 2012, 2017 and 2018 to call SNPs using state-of-the-art methods.
1.1 – First, it is not possible from this manuscript alone to assess the quality of the resequencing data, and I believe a reference was omitted L294-295 ("These new samples were prepared and submitted for whole genome sequencing following the protocol already described"). From Table S3 in Gutekunst et al. 2018 (which data were partly re-analyzed for this study), the average sequencing depth for the 2012 sample ("Animal 1") seems to be around 72x, but no information is provided in the current manuscript regarding the number of reads per individual, the sequencing strategy (I guess 2x150 bp) or the average sequencing depth in the 2017 and 2018 samples, while deep sequencing is crucial for accurate SNP calling in the context of de novo mutation detection. The reader can just assume sequencing depth is high, since "variants with quality≥35, coverage≥50 and ≤200 were retained for the main estimate of the mutation rate" (L314). More details on the data would help readers assess their quality.
>> The missing reference was included. Also, the new sequencing data was compiled in Supplementary Table 4. The per-genome sequencing coverage usually ranged between 15X and 20X per strand, which represents a reasonable compromise, given the complexities (large size, high fragmentation, triploidy) of the marbled crayfish genome.

1.2 – My second and more important point stems from this sentence L314, as authors identified mutations at sites where depth was comprised between 50 and 200 – what would be defined as callable sites. After finding candidate de novo mutations in the two more recent individuals (how many per individual? Where they at heterozygous or homozygous states?), they divided their count by the total count of nucleotides in the triploid genome

(10.5 Gb, L320). Unless authors made a typo, this is not correct: the denominator should not be the total genome size, but the number of callable sites (i.e., the fraction of the genome that could actually be surveyed). For a correct estimation of the mutation rate, authors could for example look at Lopez-Cortegano et al. (doi:10.1093/molbev/msab140, see p. 3719). I would assume this error biases downwards the mutation rate estimated for marbled crayfish, which may have implications for the calibration of the tree inferred with BEAST (Fig. 1C) and the history of the parthenogenetic lineage as a whole (e.g., L76, L269-271). Note that a similar error might have occurred with the dN/dS ratio analysis, but it is unclear in the current version how the "average quotient" was estimated for the full genome (L366). I would kindly ask the authors to estimate the mutation rate for marbled crayfish with a correct method (callable sites can easily be identified from BAM files using mosdepth, doi: 10.1093/bioinformatics/btx699), and update their findings accordingly.
>> The number of callable sites was determined and we adapted the method after (López-Cortegano et al., 2021) We now provide a range on the mutation rate, with the lower bound corresponding to mutations divided by the number of sites in the whole genome, while the upper bound corresponds to the number of mutations divided by the number of callable sites. The substantial difference between the two values is likely related to the strong influence of P. virginalis genome triploidy on the number of callable sites (see Materials and Methods for details). The methods section, the results section and figure 1 were updated accordingly.
>> The tree and the time to the most recent common ancestor remained unaffected by the mutation rate, as the calibration of the tree was done using known mating dates for animals 1, 34 and 35.
>> The dNdS ratio in hg19 was recalculated as the average quotient estimated for the full genome. We edited the Material and Methods section accordingly. The analysis was updated, as well as figure 2. However, the figures look similar, because the difference with the previous dNdS expected value is extremely small.

Major comment 2 – While I usually enjoy short papers, I believe this manuscript would benefit from a more detailed writing in many places. As it combines theory and empirical data analysis in both a non-model organism and tumors, additional details would help convey the context, importance and broad interest of these findings. Below I provide some examples of parts where I would want to get more detailed information, trying to rank these by perceived importance.
2.1 – Introduction: I would expect some framing of the scientific question before getting into the specifics of the study systems. At the moment, it is unclear what is gained by developing "an integrated analysis of clonal genome evolution", but it should become apparent in the first few paragraphs of the introduction. Likewise, the marbled crayfish is a puzzling system, and more details should be provided in the section L68-77: genetic differentiation compared to what (L74)? What is the "particular mode of asexual reproduction" (L71)? What is the specific event mentioned L76? Is parthenogenesis obligatory (are we sure there are no unintentional crosses in the lab)? More information on the origin of this species would help understand its value for the current study.
>> The introduction has been revised and expanded to better frame our study.

2.2 – Discussion: After reading the manuscript several times, I am still not sure which are the "important insights" (L286) being delivered. What does the framework tell us about clonal evolution, and/or the evolution of glioblastoma? It would help if authors drew more

connections to relevant literature. To provide an example (but as stated above, I am not an expert in cancer genomics), are the pervasive signatures of selection detected in some samples (L206) in line with recent findings (e.g., Tilk et al. 2022, doi: https://doi.org/10.7554/eLife.67790)? What is the general impact of selection on the results? What is the (clinical? biological?) relevance of the four subtypes detected (L276)? Finally, which are the potential limits of this work and what could be added?
>> The discussion has been expanded and revised to better explain the significance and the limitations of our study.

2.3 – Results: The rationale explained in the S1 file should be integrated a bit more in the main text, as the approach is currently quite vague (L133-135).
I cannot interpret the comparison of clock-like and non-clock-like mutational signatures as it stands, and I don't get why human mutational signatures are used for crayfish genomes (I must be missing something). Are expansion parameters (L231) loosely linked to any other measure used in cancer genomics? The invasion history of the marbled crayfish should be more detailed on L120-127.
>> We integrated the rationale of the S1 file in the main text, see lines 176-185. Human signatures were used as they represent (to our knowledge) the only available mutational signatures. This is justified by the conservation of mutational mechanisms across the animal kingdom and has now been clarified in the text. Non clock-like signatures do not really affect the analysis. Their description, and the comparison of non-clock-like and clock-like signatures have therefore been removed from the text. We also clarified the legend of figure 2 and focused on clock-like signatures, to improve clarity.
>> Expansion parameters: The product $\omega\gamma N$, termed expansion parameters, corresponds to the growth rate (ω), modulated by the survival (γ), and scaled by the number of individual cells (N). As such, the product $\omega\gamma N$ reflects the effective expansion of the tumor, which is why we termed this product "expansion parameters". An alternative could have been to use the denomination "effective growth" or efficient growth", but these terms are sometimes present in published articles, and almost always relate to the effect, or the efficiency, of a treatment on a tumor. Because of this, we kept the term "expansion parameters", but we explained this term in more detail in the main text.
>> The invasion history of the marbled crayfish has now been explained in the introduction.

Below are some minor comments:
L57-58: in my understanding, the term "mutation" is used for two different things here, the process itself L58 (the apparition of a new variant in the pool), and its consequence L57 (the new variant itself, i.e., a substitution). Maybe consider editing.
>> The text was clarified.

L92: unclear why "time" here.
>> The text was clarified.

L96-100: see major comment 1, but please add more information here (number of individuals, average depth…).
>> The text was supplemented with the requested information.

L105: please provide references for known mutation rates in the main text on top of the legend of Fig. 1. Also it seems there is at least one mutation rate estimated for Crustaceans (https://doi.org/10.1186/s12862-021-01836-3).
>> The references were provided, and the mutation rate of Alpheus was added to fig.1.

L109: grey colors are not displayed on the Fig. 1.
>> Fig. 1 was corrected accordingly.

L130: is there any outgroup available to root the tree? If not, is it a problem?
>> A meaningful outgroup for the marbled crayfish should be close enough without being in the same line of descent. *Procambarus fallax* is the closest relative (Gutekunst et al., 2021), but is not appropriate because *P. virginalis* is a direct descendant. Furthermore, the nuclear genomes of *P. virginalis* and *P. fallax* are markedly different, including a different ploidy and a large genetic distance (Gutekunst et al., 2018). The selection of another suitable outgroup is complicated by the fact that the genetic distance would be even larger, and by the difference in ploidy.

L144: is the dN/dS ratio actually computed from fixed differences, or from polymorphisms (so pN/pS)?
>> The dNdS ratio is computed from substitutions. Since the marbled crayfish is clonal, a substitution in one animal would in principle remain stable in any offspring of this animal, in other words fixed in this specific lineage. The substitutions may or may not represent frequent variants, or polymorphisms, in the marbled crayfish populations, but a database of frequent polymorphisms is at the moment not available for this organism.

L150: red instead of blue.
>> The legend of fig. 2 was corrected accordingly.

L153: grey instead of red.
>> The legend of fig. 2 was corrected accordingly.

L167-168: from what I understood, the dating of the marbled crayfish lineage comes from the (incorrect) estimation of the mutation rate, not the framework, which seems at odds with the statement L91-93.
>> The dating of the marbled crayfish lineage was obtained from the coalescent tree, which was calibrated using known mating dates for animals 1, 34 and 35. Because of this, the estimate for the speciation of the marbled crayfish stays unchanged (see also answer to major comment 1.2).

L174: “better understanding of *its* evolutionary parameters”.
>> The text was edited accordingly.

L178-179: Fig. 3A not 4A.
>> The text was corrected accordingly.

L204-212: this heterogeneity is interesting and could be discussed further.
>> We have expanded the discussion, in particular to discuss the heterogeneity on selection.

L244: is this only for the tumor shown in Fig. 3, or for all of them? Likewise, what is the impact of the assumption made on the TMRCA L444-445? Please clarify.
>> The durations of 2 years and 7 years are taken for all the tumors. This is an assumption taken from the values estimated in a previously published study. We clarified the text accordingly.

L262-264: this statement seems at odds with L163-165 and L266-269. More generally, this makes me wonder if I really understand how the framework disentangles between variable mutation rate and growth. This could be made clearer in the main text.
>> The statements on lines 163-165 and 266-269 lacked clarity and were edited accordingly. The difference between temporal and per division mutation rates includes the modulation by the growth rate, but also by the survival rate and the number of clones. This is now reflected in the main text.

L295: please add references for the sequencing protocol etc…
>> The reference was added (Gutekunst et al., 2018).

L302-305: the reference genome was built from short reads and is quite fragmented, is this supposed to have an impact on mutation detection? Likewise, was anything done to remove sites close to indels?
>> The reference genome is indeed highly fragmented, but the quality is comparable to other published genomes for non-standard model organisms (Gutekunst et al., 2018). This can have an impact on mutation detection, which has now been explained in the text (Methods section).

L348: define “features of the subclone”.
>> The text was edited to clarify this.

L351: is this assumption of constant ploidy reasonable for tumors? Please elaborate.
>> Ploidy undergoes more or less pronounced variations depending on the tumor type and on the tumoral sample (Steele et al., 2022). However, as a compromise between model tractability and representativity, we have assumed a constant ploidy. We have now discussed this as a limitation of our study in the main text.

L371: maybe introduce a bit what are these mutation signatures.
>> The text was expanded accordingly.

L383: are these good indicators for time in the crayfish?
>> Human signatures were used as they represent (to our knowledge) the only available mutational signatures. This is justified by the conservation of mutational mechanisms across the animal kingdom and has now been clarified in the text

L477-479: as is, I don’t get the inherent value of this classification.
>> This classification was thoroughly revised. Curves clustering by k-means was performed, and the quality of clustering was assessed using Calinski-Harabatz criterion. The text was edited accordingly.

The data availability section only mentions already published sequencing data (both for the marbled crayfish and glioblastoma). There is no mention of samples re-sequenced for this study (L290: samples Madagascar 1 and Moosweiher, L292: animal 34 and animal 35). The numerical data that underlies figures is not provided. Finally, and while it is not stated in PLOS' data availability policy, scripts are also not provided, but I think these should be available somewhere, for reproducibility and if readers are interested in your framework's implementation.
>> The sequence data from samples sequenced for this study has been successfully submitted to PRJNA356499 and will be released upon publication. The numerical data underlying the figures have been provided.

Reviewer #2

Legrand et al. apply mutational timing methods previously introduced in the cancer genomics literature to infer evolutionary parameters from two types of clonally evolving populations: one cohort of clonally evolving marbled crayfish including both published and newly generated datasets, and one published dataset of glioblastoma patients. The authors should be commended for the integrative analysis of clonal genomes in both species and cancer evolution. That being said, it is important that for this paper to be accepted, the authors address some major technical concerns summarized below.
>> We thank the reviewer for highlighting the originality of our manuscript and have made numerous changes to address all points (see details below).

Major points:

1. The authors did not account for potential confounders of the background mutation rate of each gene in their dN/dS analyses. They should control for the sequence composition of the gene and mutational signatures, by e.g. using a method like dNdScv that avoids common mutation biases affecting dN/dS using trinucleotide context-dependent substitution matrices.
>> The bias-correcting method dNdScv was tested, but could not be applied on the relatively low number of mutations at hand. Also, dNdScv does not provide longitudinal estimates. The methods section was expanded accordingly.

2. The automated segmentation of inverse allele frequency spectra M(1/f) appears to overfit the data:
> Examples of this are statements like: "In the exemplary sample 1 in Fig 3A, the segmentation separates 5 phases significantly."
> The authors should reexamine the frequency spectrum using previously published methods to segment the spectra, by distinguishing mutations in the non-clonal tail from clonal and subclonal mutations (e.g. using SNV clustering methods like MOBSTER).
>> We tested for the presence of overfitting by performing a k-fold cross-validation, using k=10 folds. The resulting validation mean square error (MSE) was 2379.382 for 4 phases, and 1183.993 for 5 phases. The difference between test MSE and validation MSE was 291,004 for 4 phases, and 135,542 for 5 phases. This showed that the difference between test and validation sets MSEs was non-increasing when shifting from 4 phases to 5 phases, indicating

the absence of overfitting. The Results section and the Material and Methods section were edited accordingly.

>> When adressing clonal to subclonal inference using modelling, several angles can be exploited, including direct inference of the genotype of each subclone (Beerenwinkel et al., 2015; Ogundijo and Wang, 2019), analysis of the frequency spectrum (Caravagna et al., 2020), or theoretical modelling. More precisely, MOBSTER utilizes a power law (Pareto type I random distribution) to model the neutral component of mutation accumulation. Subclonal clusters are assumed to follow Beta random distributions, using the same approach as used by (Miller et al., 2014), who themselves refer to Bayesian inference studies for beta mixture models and Dirichlet mixture models (Fan et al., 2012; Ma and Leijon, 2011). The assumptions taken by MOBSTER include a large population, a deterministic and exponential growth, an homogeneous process (meaning that parameters such as the mutation rate, survival rate, growth rate, ploidy, stay constant in time), and neutral mutations, except in subclones. This means that MOBSTER and other published tools rely on a markedly different set of assumptions. As a consequence, the application of these frameworks is not directly appropriate.

3. In the primary-recurrent analysis, the classification of expansion profiles is extremely weak. Expansion profiles $\omega\gamma N(t)$ are classified visually and manually curated without further explanation. This entire analysis should be removed unless a well-grounded methodology can be used to define expansion profiles (e.g. using unsupervised clustering).
>> The classification of expansion profiles was revised using curve clustering by k-means, using R package kml. The partition into 4 clusters was retained, based on the Calinski-Harabatz criterion. Figure 3 was updated accordingly, as well as the Results section and the Material and Methods section.

- Could the authors expand on the impact of ploidy on their analysis? Particularly in the case of GBM, where the tumors often have a substantial number of copy number aberrations. They should ensure to limit their timing analysis to diploid regions. Could they also speculate in the Discussion about combining their approach with the timing of copy number gains using SNVs in amplified regions?
>> Ploidy may be altered in glioblastoma, where copy number alterations are frequent (Cancer Genome Atlas Research Network, 2008; Verhaak et al., 2010). However, ploidy is generally assumed constant in mathematical models, as a simplification to keep the model as realistic as reasonably achievable. In our model, we originally attempted to consider a possibly varying ploidy. However, ploidy directly affects the naive allele frequency, as well as the apparent allele frequency in expressions (2) and (3) (Suppl. File 1), leading to more complex and probably intractable formulations of subsequent expressions. Because of this, we have assumed a constant ploidy, similarly to other studies. The impact of non-constant ploidy might affect the estimates, which could be avoided by restricting the analysis to diploid data (for GBM).
Currently, we do not have copy number information which would have allowed to restrict the analysis to diploid regions. We have stated this as a limitation, in the main text. As a perspective, one could include regions with ploidy changes, using an extension of the current model. This would mean reexamining the changes of allele frequency and the mutation rate, for each case of gain or loss of a region. In this frame, integration of copy-number

information would enrich the new model, by giving the timing of mutations in duplicated regions. This is mentioned in the main text, in the discussion.

- Could the authors demonstrate that a lower coverage threshold >15 is justified for coalescent time analyses? Are noisy variants affecting the coalescence estimates?
>> The coverage threshold had to be adapted to the available coverage for individual samples. The noisy variants can conceivably affect the coalescent estimate and constitute a limitation. This is now also discussed in our new paragraph that addresses the limitations of our paper). However, it should also be noted that the calculated/estimated coalescent time analysis fits the known biological data very well (as mentioned in the text).

Minor points:

1. In line 183, could the authors explain why they expect that mutation frequencies in the high-frequency phase (phase 1) are expected to include artifacts? This should not really be an issue unless germline variants appear to be clonal in the GBM samples and have not been exhaustively filtered out.
>> SNV calling included a matched normal sample for removal of germline variants, and scoring was performed to keep only high-confidence variants (Körber et al., 2019). Also, care was taken to allow the inclusion of high confidence, low frequency mutations. Nevertheless, mutation frequencies in the high-frequency phase may include clonal variants corresponding to an early, slow growth phase, which would violate the assumptions of the framework (Körber et al., 2019). This has now been clarified in the text.

- In lines 178 and 179, the figure callouts should refer to Fig. 3 instead of Fig. 4.
>> The figure callouts were corrected as suggested.

References

Beerenwinkel, N., Schwarz, R.F., Gerstung, M., Markowetz, F., 2015. Cancer Evolution: Mathematical Models and Computational Inference. Syst. Biol. 64, e1–e25. https://doi.org/10.1093/sysbio/syu081

Cancer Genome Atlas Research Network, 2008. Comprehensive genomic characterization defines human glioblastoma genes and core pathways. Nature 455, 1061–1068. https://doi.org/10.1038/nature07385

Caravagna, G., Sanguinetti, G., Graham, T.A., Sottoriva, A., 2020. The MOBSTER R package for tumour subclonal deconvolution from bulk DNA whole-genome sequencing data. BMC Bioinformatics 21, 531. https://doi.org/10.1186/s12859-020-03863-1

Fan, W., Bouguila, N., Ziou, D., 2012. Variational learning for finite Dirichlet mixture models and applications. IEEE Trans. Neural Netw. Learn. Syst. 23, 762–774. https://doi.org/10.1109/TNNLS.2012.2190298

Gutekunst, J., Andriantsoa, R., Falckenhayn, C., Hanna, K., Stein, W., Rasamy, J., Lyko, F., 2018. Clonal genome evolution and rapid invasive spread of the marbled crayfish. Nat. Ecol. Evol. 2, 567–573. https://doi.org/10.1038/s41559-018-0467-9

Gutekunst, J., Maiakovska, O., Hanna, K., Provataris, P., Horn, H., Wolf, S., Skelton, C.E., Dorn, N.J., Lyko, F., 2021. Phylogeographic reconstruction of the marbled crayfish origin. Commun. Biol. 4, 1096. https://doi.org/10.1038/s42003-021-02609-w

Körber, V., Yang, J., Barah, P., Wu, Y., Stichel, D., Gu, Z., Fletcher, M.N.C., Jones, D., Hentschel, B., Lamszus, K., Tonn, J.C., Schackert, G., Sabel, M., Felsberg, J., Zacher, A., Kaulich, K., Hübschmann, D., Herold-Mende, C., Deimling, A. von, Weller, M., Radlwimmer, B., Schlesner, M., Reifenberger, G., Höfer, T., Lichter, P., 2019. Evolutionary Trajectories of IDHWT Glioblastomas Reveal a Common Path of Early Tumorigenesis Instigated Years ahead of Initial Diagnosis. Cancer Cell 35, 692-704.e12. https://doi.org/10.1016/j.ccell.2019.02.007

López-Cortegano, E., Craig, R.J., Chebib, J., Samuels, T., Morgan, A.D., Kraemer, S.A., Böndel, K.B., Ness, R.W., Colegrave, N., Keightley, P.D., 2021. De Novo Mutation Rate Variation and Its Determinants in Chlamydomonas. Mol. Biol. Evol. 38, 3709–3723. https://doi.org/10.1093/molbev/msab140

Ma, Z., Leijon, A., 2011. Bayesian estimation of beta mixture models with variational inference. IEEE Trans. Pattern Anal. Mach. Intell. 33, 2160–2173. https://doi.org/10.1109/TPAMI.2011.63

Miller, C.A., White, B.S., Dees, N.D., Griffith, M., Welch, J.S., Griffith, O.L., Vij, R., Tomasson, M.H., Graubert, T.A., Walter, M.J., Ellis, M.J., Schierding, W., DiPersio, J.F., Ley, T.J., Mardis, E.R., Wilson, R.K., Ding, L., 2014. SciClone: inferring clonal architecture and tracking the spatial and temporal patterns of tumor evolution. PLoS Comput. Biol. 10, e1003665. https://doi.org/10.1371/journal.pcbi.1003665

Ogundijo, O.E., Wang, X., 2019. SeqClone: sequential Monte Carlo based inference of tumor subclones. BMC Bioinformatics 20, 6. https://doi.org/10.1186/s12859-018-2562-y

Steele, C.D., Abbasi, A., Islam, S.M.A., Bowes, A.L., Khandekar, A., Haase, K., Hames-Fathi, S., Ajayi, D., Verfaillie, A., Dhami, P., McLatchie, A., Lechner, M., Light, N., Shlien, A., Malkin, D., Feber, A., Proszek, P., Lesluyes, T., Mertens, F., Flanagan, A.M., Tarabichi, M., Van Loo, P., Alexandrov, L.B., Pillay, N., 2022. Signatures of copy number alterations in human cancer. Nature 606, 984–991. https://doi.org/10.1038/s41586-022-04738-6

Verhaak, R.G.W., Hoadley, K.A., Purdom, E., Wang, V., Qi, Y., Wilkerson, M.D., Miller, C.R., Ding, L., Golub, T., Mesirov, J.P., Alexe, G., Lawrence, M., O'Kelly, M., Tamayo, P., Weir, B.A., Gabriel, S., Winckler, W., Gupta, S., Jakkula, L., Feiler, H.S., Hodgson, J.G., James, C.D., Sarkaria, J.N., Brennan, C., Kahn, A., Spellman, P.T., Wilson, R.K., Speed, T.P., Gray, J.W., Meyerson, M., Getz, G., Perou, C.M., Hayes, D.N., Cancer Genome Atlas Research Network, 2010. Integrated genomic analysis identifies clinically relevant subtypes of glioblastoma characterized by abnormalities in PDGFRA, IDH1, EGFR, and NF1. Cancer Cell 17, 98–110. https://doi.org/10.1016/j.ccr.2009.12.020
